# High-throughput RNAi screening of human kinases identifies predictors of clinical outcome in colorectal cancer patients treated with oxaliplatin

**DOI:** 10.18632/oncotarget.3736

**Published:** 2015-03-30

**Authors:** Moubin Lin, Yajie Zhang, Ajian Li, Erjiang Tang, Jian Peng, Wenxian Tang, Yong Zhang, Liang Lu, Yihua Xiao, Qing Wei, Lu Yin, Huaguang Li

**Affiliations:** ^1^ Center for Translational Medicine, Yangpu Hospital, Tongji University School of Medicine, Shanghai, China; ^2^ Department of General Surgery, RuiJin Hospital Affiliated to Shanghai Jiaotong University School of Medicine, Shanghai, China; ^3^ Department of General Surgery, Yangpu Hospital, Tongji University School of Medicine, Shanghai, China; ^4^ Department of Pathology, The Tenth People's Hospital Affiliated to Shanghai Tongji University School of Medicine, Shanghai, China

**Keywords:** colorectal cancer, RNAi screening, chemotherapy, recurrence, survival

## Abstract

The purpose of this study is to identify protein kinase genes that modulate oxaliplatin cytotoxicity *in vitro* and evaluate the roles of these genes in predicting clinical outcomes in CRC patients receiving oxaliplatin-based adjuvant chemotherapy. A high-throughput RNAi screening targeting 626 human kinase genes was performed to identify kinase genes whose inhibition potentiates oxaliplatin sensitivity in CRC cells. The associations between copy numbers of the candidate genes and recurrence-free survival and overall survival were analyzed in 142 stage III CRC patients receiving first-line oxaliplatin-based adjuvant chemotherapy who were enrolled from two independent hospitals. HT-RNAi screening identified 40 kinase genes whose inhibition potentiated oxaliplatin cytotoxicity in DLD1 cells. The relative copy number (RCN) of MAP4K1 and CDKL4 were associated with increased risks of both recurrence and death. Moreover, significant genes-based risk score and the ratios of RCN of different genes can further categorize patients into subgroups with distinctly differing outcomes. The estimated AUC for the prediction models including clinical variables plus kinase biomarkers was 0.77 for the recurrence and 0.82 for the survival models. The copy numbers of MAP4K1 and CDKL4 can predict clinical outcomes in CRC patients treated with oxaliplatin-based chemotherapy.

## INTRODUCTION

Colorectal cancer (CRC) is the third most common malignancies and the second leading cause of cancer death worldwide [1]. The clinical outcome of CRC has significantly improved over recent decades, which can be attributed to improved surgical techniques in early disease, adjuvant therapy for locally advanced disease, and multimodality therapy for metastatic disease [2–4]. Adjuvant chemotherapy is recommended in patients with high risk stage II and stage III patients after curative resection according to the National Comprehensive Cancer Network (NCCN) Guidelines. The results from MOSAIC and NSABP C-07 clinical trial led to the approval of oxaliplatin-based regimens as the first-line chemotherapy in the adjuvant setting [3, 5]. However, three-year disease-free survival (DFS) continues to be 65% in patients with stage III CRC receiving oxaliplatin-containing adjuvant chemotherapy [6]. To identify genes involved in modulating oxaliplatin response would be instrumental in improving the efficacy of adjuvant oxaliplatin–based chemotherapy and predicting which patients are most or least likely to benefit from such treatment. To date, no predictive markers of oxaliplatin sensitivity and resistance have been clinically validated.

With the advent of high-throughput RNA interference (HT-RNAi) screen-based on large-scale gene knockdown, it has become feasible to quickly screen and validate drug modulators whose suppression can overcome resistance to chemotherapeutic agents [7]. Such studies can not only provide important insight into the mechanism of drug resistance, but also identify novel drug targets and potential combination strategies for more effective chemotherapy, and identify promising candidates to serve as biomarkers for drug response and patient stratification [8]. A number of studies utilizing HT-RNAi screen have characterized key genes contributing to the resistance to chemotherapeutic agents in patients with breast, ovarian, and other cancers [9, 10]. However, there was only one published study using HT-RNAi screen-based approach to identify modulators of oxaliplatin resistance in CRC cell lines that revealed the involvement of TP53 in oxaliplatin response [11].

Protein kinases function as key regulators of cell proliferation, differentiation and survival through the process of phosphorylation. Protein kinases are believed to play an important role in chemoresistance based on the evidence that inhibition of kinases often enhances sensitivity of cancer cells to chemotherapeutic agents including cisplatin and doxorubicin [12]. Aberrant activation of kinases was frequently observed in tumors due to somatic genome aberrations including mutations and copy number variations (CNVs). Increased copy number of aurora kinase A was associated with prognosis and response to oxaliplatin–based chemotherapy in patients with metastatic CRC [13]. However, there has not been any study published using HT-RNAi screening of the kinome to identify modulators of oxaliplatin-based chemotherapy response in locally advanced CRC. We hypothesize that CNVs of kinases are potential predictors of oxaliplatin efficacy in the adjuvant setting. To test this hypothesis, we first employed HT-RNAi screening using a lentiviral short hairpin RNA (shRNA) library against human kinome to identify potentiators of oxaliplatin response in cultured CRC cell lines. Subsequently, we used two independent CRC patient cohorts to validate the association between CNVs of “hit” genes and clinical outcomes of stage III CRC receiving oxaliplatin-based chemotherapy.

## RESULTS

### High-throughput RNAi screening for kinase genes modulating oxaliplatin response

**Figure 1:**
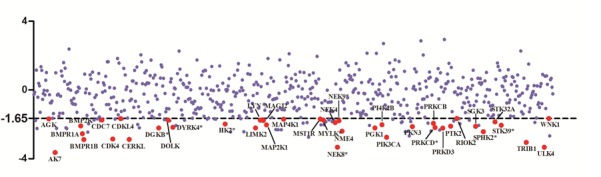
Scatter plot of z score from RNAi screen Oxaliplatin resistance screen with human kinome lenti-shRNA library was carried out. The calculated z score corresponding to each target gene was dotted and target genes with z score <-1.65 and SI>0.1 were selected as hits and showed in red solid circles.

We performed HT-RNAi screen using a lentiviral shRNA library that targets 626 human kinase genes. Both statistics and sensitivity parameters were used for selection of “hits” [9, 14]. Only lentiviral shRNA fulfilling the requirements of Z-score < −1.65 (equal to *P* < 0.05, the standard of statistical significance) and SI>0.15 (standard for sensitizing) were identified as hits. The preliminary screening, as shown in Table 1, Figure 1 and Supplementary Table 1, yielded 40 (6.4%) genes whose inhibition by shRNA potentiated DLD1 cell sensitivity to oxaliplatin, suggesting the involvement of these genes in oxaliplatin resistance.

**Table 1:** Functional categories of kinase hits

Gene Symbol	z-score	SI	Functional categories
*AK7*	−3.63	0.26	Metabolic regulation
*ULK4*	−3.33	0.25	MAPK signaling
NEK8*	−3.33	0.38	Cell cycle regulation
*TRIB1*	−3.05	0.17	MAPK signaling
*BMPR1B*	−2.88	0.3	TGF-β signaling
*CERKL*	−2.88	0.25	Lipid signaling
*CDK4*	−2.85	0.2	Cell cycle regulation
*PIK3CA*	−2.75	0.24	Lipid signaling
*BMPR1A*	−2.54	0.2	TGF-β signaling
SPHK2*	−2.42	0.2	Lipid signaling
*NME4*	−2.39	0.16	Metabolic regulation
*PRKD3*	−2.22	0.26	AGC kinase
DGKB*	−2.21	0.25	Metabolic regulation
*LIMK2*	−2.21	0.43	Tyrosine kinase signaling
*PGK1*	−2.21	0.15	Metabolic regulation
PRKCD*	−2.19	0.18	AGC kinase
DYRK4*	−2.17	0.18	Tyrosine kinase signaling
*PKN3*	−2.13	0.2	PI3K-AKT signaling
*SGK3*	−2.11	0.29	PI3K-AKT signaling
*PTK2*	−2.11	0.34	Tyrosine kinase signaling
*BMP2K*	−2.09	0.17	Serine/threonine kinse signaling
STK39*	−2.05	0.22	MAPK signaling
*MAP2K1*	−2.03	0.16	MAPK signaling
*PI4K2B*	−2.02	0.18	Lipid signaling
HK2*	−1.98	0.31	Metabolic regulation
*PRKCB*	−1.94	0.18	AGC kinase
*NEK4*	−1.89	0.46	Cell cycle regulation
*STK32A*	−1.86	0.19	Serine/threonine-protein kinase
NEK9*	−1.79	0.17	Cell cycle regulation
*MAGI2*	−1.76	0.28	PI3K-AKT signaling
*LYN*	−1.75	0.28	MAPK signaling
*MYLK*	−1.75	0.36	Calcium signaling
*DOLK*	−1.75	0.24	Metabolic regulation
*CDC7*	−1.74	0.17	Cell cycle regulation
*MAP4K1*	−1.7	0.27	MAPK signaling
*MST1R*	−1.69	0.19	MAPK signaling
*AGK*	−1.68	0.18	Lipid signaling
*CDKL4*	−1.67	0.16	Cell cycle regulation
*WNK1*	−1.66	0.23	Serine/threonine kinse signaling
*RIOK2*	−1.66	0.33	Serine/threonine-protein kinase

* Note. Genes also found in other RNAi screens to be resistant to chemotherapy.

To further explore the underlying mechanism of oxaliplatin resistance, we performed a pathway analysis of the 40 hits through the DAVID (http://david.abcc.ncifcrf.gov/) analysis tool. Several cancer-related pathways were identified. Under a stringent criterion of *P* < 0.01, the *VEGF* and *ERBB* signaling pathways were enriched with four key genes comprising *PTK2*, *MAP2K1*, *PIK3CA* and *PRKCB*.

### Validation of kinase genes identified from HT-RNAi screening in CRC patients

We then used two independent cohorts of CRC patients receiving adjuvant oxalilplatin-based chemotherapy to evaluate whether the *in vitro* HT-RNAi screen-identified kinase genes are involved in oxaliplatin response in patients. Supplementary Table 2 shows the clinical characteristics of the training and validation patient cohorts. Of the 76 patients in the training set, the median age was 60 years (range, 24–79 years). The median follow-up time was 57 months. There were 19 recurrences (25%) and 28 deaths (36.8%), but the median RFS and OS had not been reached during the follow-up time. Among the 66 patients in the replication set, 19 patients (28.8%) had recurrence and 15 (22.7%) were dead. There was no significant difference between the training and validation sets with regard to age, gender, histological grade and stage.

We assessed the association of the RCN of each hit gene with recurrence and survival using a multivariate Cox model, adjusting for age, gender, tumor site and histologic grade. In the training set, a significantly increased risk of recurrence was observed for those with higher RCN of *MAP4K1* (HR 1.15, 95%CI, 1.05-1.26; P=0.002) and *CDKL4* (HR, 1.20, 95%CI, 1.02-1.40; P=0.02) (Supplementary Table 3 and Table 2). Higher RCN of three genes exhibited significantly increased risk of death: *MAP4K1* (HR 1.11; 95%CI, 1.03-1.19; P=0.01), *DOLK* (HR 1.19; 95%CI, 1.00-1.41; P=0.04) and *CDKL4* (HR 1.14; 95%CI, 1.00-1.29; P=0.05) (Supplementary Table 4 and Table 2).

**Table 2:** Copy number of individual genes associated with clinical outcomes of stage III CRC patients

Gene	Training set		Replication set		Pooled analysis	
	HR(95% CI)^a^	P	HR(95% CI)^a^	P	HR(95% CI)^a^	P
**Recurrence**						
*MAP4K1*	1.15(1.05-1.26)	**0.002**	1.16(1.01-1.32)	**0.04**	1.14(1.06-1.22)	**0.0002**
*CDKL4*	1.20(1.02-1.40)	**0.02**	1.22(0.84-1.76)	0.29	1.17(1.02-1.35)	**0.03**
**Survival**						
*MAP4K1*	1.11(1.03-1.19)	**0.01**	1.20(1.04-1.38)	**0.01**	1.11(1.05-1.18)	**0.0005**
*DOLK*	1.19(1.00-1.41)	**0.04**	0.76(0.26-2.21)	0.61	1.07(0.92-1.24)	0.36
*CDKL4*	1.14(1.00-1.29)	**0.05**	1.53(1.06-2.19)	**0.02**	1.15(1.03-1.28)	**0.01**

a Note. Adjuster for age, sex, stage and histological grade.

The significant associations between *MAP4K1* and the risks of recurrence and death were confirmed in the replication set and pooled analysis (Supplementary Table 3 and 4). Patients with higher RCN of *MAP4K1* showed increased risks of recurrence (HR 1.16; 95% CI, 1.01-1.32; P=0.04) and death (HR 1.20; 95% CI, 1.04-1.38; P=0.01) in the replication set. The combined HR was 1.14 (95% CI, 1.06 to1.22; P=0.0002) for recurrence and 1.11 (95%CI, 1.05-1.18; P=0.0005) for death. Although *CDKL4* exhibited similar risk estimate with recurrence in the validation set, the association did not reach statistical significance. However, the increased risk of death associated with higher RCN of *CDKL4* was confirmed in the replication set (HR 1.53; 95%CI, 1.06-2.19, P=0.02) and combined dataset (HR 1.15; 95% CI, 1.03-1.28, P=0.01).

### The association between RCN-based risk score and overall survival

**Figure 2:**
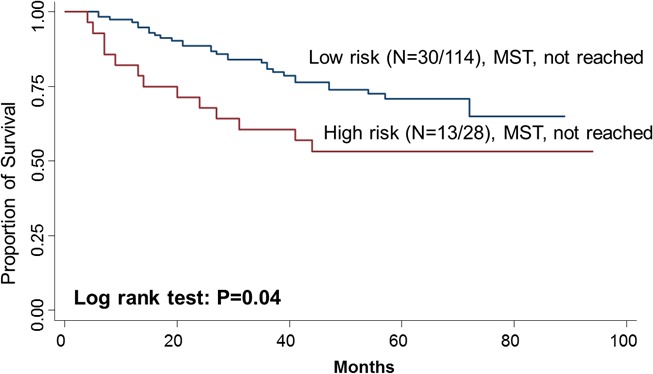
Kaplan–Meier OS curves of CRC in all patients based on the risk score of *MAP4K1* and *CDKL4*

To further evaluate the predictive value of *MAP4K1* and *CDKL4* in survival, we performed a risk score analysis. Joint analysis of *MAP4K1* and *CDKL4* dichotomized the 142 patients into low-risk and high-risk group for death using the cutoff (0.45) of the risk score threshold value. The high-risk group had a 2.70-fold (95% CI, 1.37-5.33) increased risk and had a significantly shorter median survival time compared with the low-risk group (log-rank P=0.04) (Figure 2).

### The association between copy number ratios of hit genes and clinical outcomes

**Figure 3:**
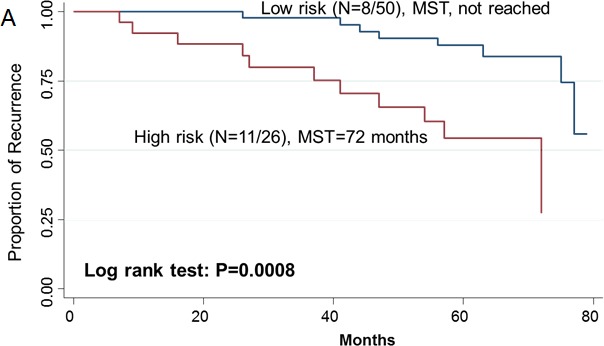

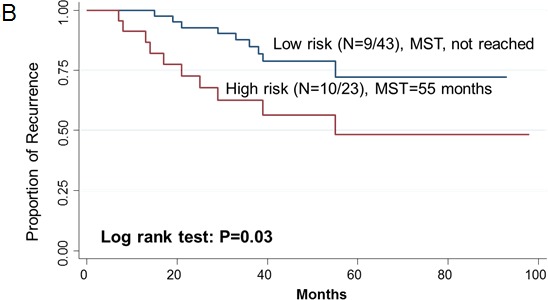

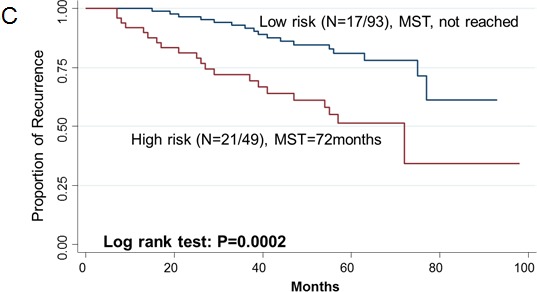

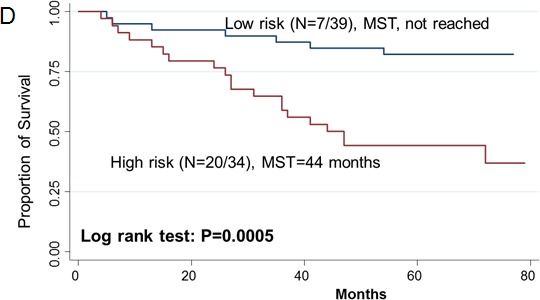

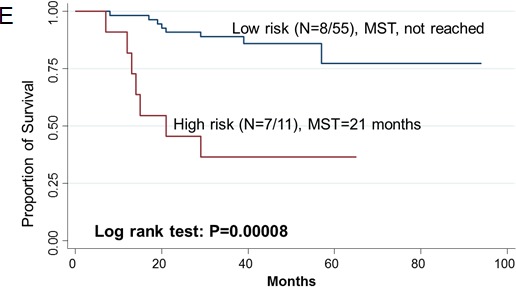

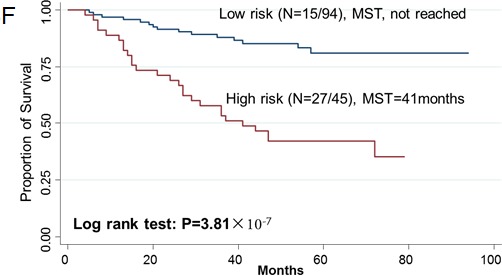
Kaplan-Meier RFS and OS curves of CRC patients Kaplan–Meier RFS curves of patients based on joint ratios of *PTK2/MAGI2* and *CDKL4/PTK2* in training set (**A**), replication set (**B**), and combined set (**C**). Kaplan–Meier OS curves of patients based on the joint ratios of *PRKCD/BMP2K, MST1R/WNK1, STK39/AGK, DYRK4/SGK3* and *CDKL4/CERKL* in training set (**D**), replication set (**E**), and combined set (**F**). MST, median event-free survival times.

Since two-gene ratios have been used to evaluate cancer prognosis and provided improved risk prediction in breast cancer [15], we performed similar analyses. The RCN of each hit gene was compared to the RCN of the other 39 hit genes, which produced 780 ratios with intragroup variance. To reduce false positives, the ratios with intragroup variability below 10 (288 ratios) were used for the analyses of clinical outcomes (Supplementary Table 5). There were 29 ratios and 33 ratios significantly associated with the risk of recurrence and death, respectively (Supplementary Table 6, 7). Individual and joint analysis of the ratios of *PTK2*/*MAGI2* and *CDKL4*/*PTK2* showed a consistent association with the risk of recurrence in the training set, replication set and combined analysis (Supplementary Table 8). Joint analysis of these two ratios could categorize patients into two subgroups. Compared with the low-risk group, the high-risk group had a 2.91-fold (95% CI, 1.49-5.69) increased risk of recurrence. The median RFS time for the low-risk and high-risk group was >94 months and 72 months (log-rank P=0.0002), respectively (Figure 3). The ratios of *PRKCD*/*BMP2K*, *MST1R*/*WNK1*, *STK39*/*AGK*, *DYRK4*/*SGK3* and *CDKL4*/*CERKL* were significantly associated with the risk of death in the training set, replication set and combined analysis (Supplementary Table 8). The joint analysis of these 5 ratios showed that the high-risk group was at a 5.29-fold (95% CI 2.61–10.72) increased risk of death. The median overall survival time for the low-risk and high-risk group was >94 months and 41 months (log-rank P=3.81×10^−7^), respectively (Figure 3).

### ROC model for evaluating the prediction ability of clinical outcome

**Figure 4:**
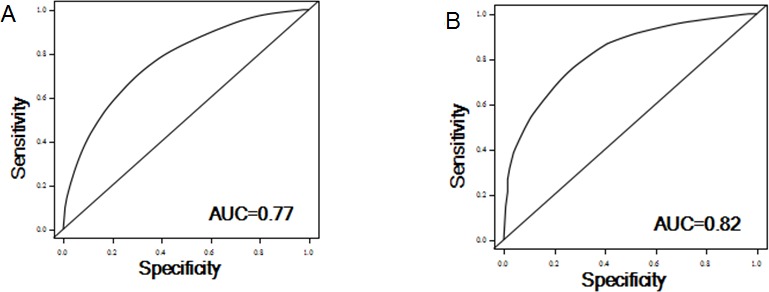
ROC curve analysis for the risk of recurrence (A) and survival (B) based on the prediction model

To evaluate the discriminatory ability, we constructed ROC and computed the AUC for risk prediction models of recurrence and death. For the prediction ability of recurrence, when we incorporated clinical variables (age, sex, stage and histological grade), effect of *MAP4K1* and joint ratios of *PTK2*/*MAGI2* and *CDKL4*/*PTK2*, the AUC for all patients (n =142) was 0.77 (0.62-0.74). The sensitivity and specificity was 78% and 65%, respectively. For survival prediction, the AUC reached 0.82 (0.71-0.85), when we included clinical variables, combined risk score of *MAP4K1* and *CDKL4*, and combined ratios of *PRKCD*/*BMP2K*, *MST1R*/*WNK1*, *STK39*/*AGK*, *DYRK4*/*SGK3* and *CDKL4*/*CERKL* in the model. The sensitivity and specificity was 85% and 69%, respectively (Figure 4).

## DISCUSSION

To our knowledge, this is the first study to identify modulators of oxaliplatin response by a combination of HT-RNAi functional screen of human kinases and copy number analysis of human patient samples. In this study, we found 40 kinase genes responsible for oxaliplatin response through the kinome HT-RNAi screen, 9 of which (22.5%) have been reported to be involved in chemoresistance in other RNAi screens [7, 14]. Further, we found that high copy number of *MAP4K1* showed consistent association with both recurrence and survival for patients with stage III CRC patients receiving oxaliplatin-based chemotherapy, highlighting the important role of *MAP4K1* in modulating oxaliplatin response and its potential as a biomarker for the efficacy of oxaliplatin-based chemotherapy.

*MAP4K1* exerts its biological function upon extracellular stimulation by activating *MAP3* kinases which in turn phosphorylate *JNK*, leading to the activation of *MAPK* pathway [16]. *JNK* has been reported in colon and other cancer cells to sustain multidrug resistance (MDR), and inhibition of *JNK* enhanced the apoptosis of colon cancer cells treated with chemotherapy agents such as nocodazole, camptothecin and 5-FU [17–19]. Enforced *MAP4K1* expression was also shown to stimulate *NFκB* pathway which impeded apoptosis, resulting in resistance to cytotoxic drugs in cancer cell lines [20, 21]. Therefore, the function of *MAP4K1* contributing to MDR and anti-apoptosis would account for the observation that CRC patients with high copy number of *MAP4K1* had a worse clinical outcome. This finding was also supported by recent studies on another *MAP4* kinase-*MAP4K4*, whose high expression was found to be associated with poor prognosis in hepatocellular carcinoma and pancreatic ductal adenocarcinoma [22, 23].

Our study also showed that a significant association of *CDKL4* with both recurrence and death. *CDKL4* belongs to cyclin-dependent protein kinase (CDK) family responsible for cell cycle progression. In colon cancer cells, CDK inhibitors were demonstrated to inhibit cell proliferation and induce cell cycle arrest and apoptosis [24]. Furthermore, inhibition of CDKs enhanced cell death in response to chemotherapy through downregulation of anti-apoptotic proteins including *XIAP*, *BCL2*, *MCL-1* and suppression of survivin phposphorylation [25]. In a phase I study of CDK inhibitor (Flavopiridol) administered with oxaliplatin-based chemotherapy in platinum-refractory germ cell tumors, 33% of patients showed a partial response [26]. Consistent with this, our HT-RNAi screen results showed increased colon cancer cell death after targeting *CDKL4* in the presence of oxaliplatin.

The present study also showed that the risk score of *MAP4K1* and *CDKL4* could affect CRC overall survival. Activated *JNK* driven by *MAP4* kinases is able to phosphorylate *c-Jun*, which subsequently promote gene transcription responsible for G1/S and G2/M transition [27, 28]. Therefore, *MAP4K1* possibly coordinated with *CDKL4* to achieve cumulative effect on cell cycle progression. We also evaluated the effect of copy number ratios of hit genes on clinical outcomes. Among them, *MST1R*/*WNK1*, *STK39*/*AGK*, *DYRK4*/*SGK3* and *CDKL4*/*PTK2* have cumulative effect on overall survival. Interestingly, all these hit genes have been implicated in *MAPK* and *PI3K* signaling pathways. *In vitro* experiments have demonstrated the complex interactions between components of the *PI3K* and *MAPK* pathway, providing a rationale for the combined inhibition of both pathways to confer enhanced sensitivity to chemotherapeutics agents [29].

When we incorporated all the potential predictive markers revealed in this study into a multivariate model, we obtained excellent prediction efficacy with an AUC of 0.77 for recurrence and 0.82 for survival, highlighting the importance of considering biomarkers in prediction of clinical outcomes. The risk models to predict clinical efficacy may allow clinicians to identify patients at high risk of recurrence and survival before the start of therapy and make better-informed clinical decisions and follow up surveillance for patients.

Furthermore, a pathway analysis revealed that the *VEGF* and *ERBB* signaling pathway genes were enriched in modulating oxaliplatin response, suggesting drugs targeting these two pathways may enhance the sensitivity of CRC cells to oxaliplatin. Indeed, targeted therapies by Avastin and Cetuximab have now been used in combination with oxaliplatin-based chemotherapy to treat metastatic CRC [30, 31]. Finally, our study uncovered several potential drug targets that may overcome oxaliplatin resistance and improve therapy. This is particularly significant as acquired resistance to Avastin and Cetuximab ultimately takes place after a period of usage.

In conclusion, using HT-RNAi screen followed by patient cohort validation, we have identified several plausible kinase genes that are associated with oxaliplatin resistance in CRC cell lines. These genes are potential drug targets to overcome oxiliplatin resistance and their copy numbers may serve as biomarkers for clinical outcomes in CRC patients receiving oxaliplatin-based chemotherapy.

## MATERIALS AND METHODS

### Cell lines

DLD1 was purchased from American Type Culture Collection (ATCC, United States). 293FT was obtained from life technologies (United States). DLD1 was cultured in RPMI-1640 medium supplemented with 10% FBS. 293FT cells were maintained in DMEM supplemented with 10% FBS, 0.1mmol/L MEM non-essential amino acids, 6 mmol L-glutamine, 1mmol/L sodium pyruvate and 1% penicillin/streptomycin, 500μg/mL geneticin.

### Lentivial shRNA library construction

The shRNA sequences targeting 626 human kinase genes (Supplementary Table 1) were generated using the design tool from the Broad Institute RNAi Consortium (http://www.broadinstitute.org/rnai/public/). Four pairs of shRNA oligonucleotides for each gene were selected, chemically-synthesized and then constructed into pLvUCTP lentiviral vector which was modified from FuGW as we previously described [32]. The library construction method was modified from the protocol described elsewhere [33]. Briefly, four lentiviral shRNA vectors for each gene were prepared and mixed equally. Transfections were carried out in 24-well plates by adding the mixture of shRNA vectors, packaging plasmids and FUGENE (Roche, Switzerland). Lentiviral supernatants were harvested at 24, 48 and 60 h post-transfection and purified by passaging into 0.45 μm filter (Millipore, Germany). Collected lentiviruses were stored at −80°C for long storage.

### HT-RNAi screen and hits selection and validation

For HT-RNAi screen, we used DLD1 cell line because our previous study has shown that DLD1 was the most resistant to oxaliplatin among 17 CRC cell lines [32]. DLD1 cells were seeded in 96-well plates at the density of 2×10^3^ cells per well. The next day, medium was removed and replaced with fresh medium supplemented with 8 μg/ml polybrene (Sigma, United States), and then lentiviral shRNAs were added to cells at a multiplicity of infection (MOI) of 5 in triplicates. Equal amount of lentiviral scramble shRNA was placed in each plate as a control [32]. After 24 h, cells were treated with equal volume of vehicle control (water) and 10 μM oxaliplatin (Sigma, United States) solution; this concentration resulted in an average of 40% cell growth inhibition and was close to the plasma concentration in CRC in our previous study [32]. At 48 h post-infection, cells were subjected to MTT assay. Z-score and sensitivity index (SI) value were utilized as the criteria for hits selection. The calculation of Z score and SI were performed according to the protocols described previously [9, 14]. The Z score and SI of lentiviral shRNA-treated cells were normalized to those of the scramble shRNA-treated cells.

### CRC patient cohort and data collection

142 histologically confirmed stage III colorectal adenocarcinoma patients were recruited between June 2006 and December 2011, with follow-up to January 2014. Among them, 76 patients were recruited from RuiJin Hospital affiliated to Shanghai Jiaotong University and used as the training set, and 66 patients were enrolled from The Tenth People's Hospital affiliated to Shanghai Tongji University and used as the validation set. All the patients were newly diagnosed cases (diagnosed within 1 year of enrollment) and none had undergone chemotherapy or radiotherapy prior to study enrollment. All the patients underwent radical resection and first-line oxaliplatin-based adjuvant chemotherapy for at least of six cycles. Patients were staged according to the American Joint Committee on Cancer-tumor-node metastasis staging system (TNM). We reviewed patients' medical records to collect clinical information including date of diagnosis, clinical stage, tumor location, histological grade, pathological stage and treatment. The information of recurrence/death was obtained from the medical records or telephone follow-up. Paraffin-embedded (FFPE) specimens were provided by pathological departments. The study was approved by the Human Research Ethics Committees of Shanghai Jiaotong University and TongJi University.

### DNA extraction from FFPE samples

Manual macrodissection of tumor samples were performed according to the protocol described elsewhere [34, 35]. 50-100 tissue sections (2 μm thick per each) were sliced from each FFPE sample. Hematoxylin and eosin (H&E)-stained tissue sections were reviewed by three pathologists. Manual macrodissection was performed only when three pathologists reached consensus. The tumor samples were collected by macrodissection of area with over 70% neoplastic cells and subjected to DNA extraction using DNA FFPE kit (Qiagen, German).

### Quantitative PCR and copy number analysis

20 ng of genomic DNA from each sample was subjected to Quantitative PCR using the SYBR Green kit (Qiagen, Germany). The PCR program was 15 s of denaturation at 95°C, 20 s of primer annealing at 58°C and 20 s of extension at 72°C. GAPDH was used as the reference gene. Each assay was run in duplicates. Data points that generated duplicated Ct values with over one cycle variance were excluded from analysis. The Ct value obtained from each sample was normalized to the mean Ct of all samples and then subjected to analysis with the 2^−ΔΔCt^ method [36]. Briefly, the mean Ct for each gene in all samples was firstly calculated and ΔCt was computed by subtracting individual Ct from mean Ct. ΔΔCt was then computed by subtracting ΔCt of a target gene from ΔCt of the reference gene (*GAPDH*). Finally, 2^−ΔΔCt^ was calculated and defined as relative copy number (RCN) of each target gene [36].

### Statistical analysis

Statistical analyses were conducted using Intercooled STATA software, version 10 (College Station). Chi-square (x2) analysis was used to evaluate differences in patient characteristics of categorical variables and Student's t test was used to assess the difference of continuous variables. The study endpoints were recurrence-free survival (RFS) and overall survival (OS). RFS was defined as the time from pathologic diagnosis until first recurrence. OS was calculated from pathologic diagnosis to death. Due to the challenge in the normalization of gene copy number, we followed the procedure described in Boeri *et al* [37] to compute the ratio of RCN between high copy number genes and restricted the analysis to 278 ratios with minimal individual variation with intra-group variance ≤10 to reduce the potential false positive findings. To assess the association of RCN with the recurrence and survival of CRC, multivariate Cox proportional hazards model was used to estimate HRs and their 95% confidence intervals (CI) in the training set, the replication set and pool set, adjusting for age, gender, stage and histological grade. Kaplan–Meier method was used to plot RFS and OS curves with the log-rank test to compare curves. We also evaluated the combined effects of the significant genes based on their RCN. For the ratios exhibiting significant association with recurrence or survival in both the training and validation data, we constructed the risk scores from all the possible combinations of the ratios. The risk score of combined genes for each patient was derived by linear combination of the product of reference-normalized copy number of each gene by its Cox regression corresponding coefficient. We also dichotomized patients into two groups using a cut off value of the ratio generated by the spline regression method. We constructed receiver operating characteristic (ROC) curves and calculated the area under the curve (AUC) to evaluate the specificity and sensitivity of predicting recurrence and survival. All P values were 2-sided and a *P* ≤ 0.05 was considered statistically significant.

## SUPPLEMENTARY MATERIAL TABLES


