# Supplementary material for: High-throughput RNAi screening of human kinases identifies predictors of clinical outcome in colorectal cancer patients treated with oxaliplatin
Source: Oncotarget. 2015 Mar 30;6(18):16774–85. doi: 10.18632/oncotarget.3736 (PMC4599307; doi:10.18632/oncotarget.3736)
Supplement: Supplementary file 1 [file oncotarget-06-16774-s001.pdf]

High-throughput RNAi screening of human kinases identifies predictors of clinical outcome in colorectal cancer patients treated with oxaliplatin

Supplementary Material

Table S1. Primary analysis of kinome RNAi screen

Gene symbol	z score	SI
NEK4	-1.89	0.46
LIMK2	-2.21	0.43
NEK8	-3.33	0.38
EPHB2	-1.45	0.38
MYLK	-1.75	0.36
PTK2	-2.11	0.34
RIOK2	-1.66	0.33
EPHB1	-1.16	0.32
HK2	-1.98	0.31
BMPR1B	-2.88	0.30
SGK3	-2.11	0.29
TNNI3K	-1.39	0.29
MAGI2	-1.76	0.28
LYN	-1.75	0.28
MAP4K1	-1.70	0.27
PRKD3	-2.22	0.26
AK7	-3.63	0.26
PACSIN1	-0.01	0.25
DGKB	-2.21	0.25
CERKL	-2.88	0.25
MAPKAPK2	-1.09	0.25
ULK4	-3.33	0.25
PIK3CA	-2.75	0.24
DOLK	-1.75	0.24

TYK2	-1.63	0.24
WNK1	-1.66	0.23
PRKCSH	-0.52	0.23
DAK	-1.53	0.22
STK39	-2.05	0.22
TNK1	-1.26	0.22
LCK	-0.33	0.22
PDK2	-1.45	0.21
MAPK8	-1.01	0.21
PFKFB2	-0.80	0.21
MARK3	-0.51	0.21
SRC	-0.69	0.21
IL1A	-1.55	0.20
BMPR1A	-2.54	0.20
RPS6KA3	-1.32	0.20
SPHK2	-2.42	0.20
PKN3	-2.13	0.20
CDK4	-2.85	0.20
STK32A	-1.86	0.19
MST1R	-1.69	0.19
PTK6	-1.26	0.18
AGK	-1.68	0.18
PI4K2B	-2.02	0.18
TEC	0.49	0.18
FGGY	-0.65	0.18
IGF1R	-1.24	0.18
DYRK4	-2.17	0.18
PRKCB	-1.94	0.18
EEF2K	-0.26	0.18

VRK2	-1.08	0.18
UCKL1	-1.02	0.18
CDC42BPB	-1.57	0.18
PRKCD	-2.19	0.18
NEK9	-1.79	0.17
CDC7	-1.74	0.17
CAMK1	-1.14	0.17
BMP2K	-2.09	0.17
TRIB1	-3.05	0.17
PFKL	-1.09	0.17
CINP	-1.00	0.16
JAKMIP1	-1.30	0.16
CDKL4	-1.67	0.16
TAOK1	-0.58	0.16
NME4	-2.39	0.16
MAP2K1	-2.03	0.16
IKBKB	-0.86	0.16
GK2	-0.27	0.16
NME7	-1.11	0.16
MAP3K1	-1.61	0.16
CDK2	-0.84	0.15
PGK1	-2.21	0.15
STK36	-1.48	0.15
CDK7	-1.18	0.15
PAK6	-0.78	0.15
GALK1	-0.23	0.15
ULK2	-0.80	0.15
BRD3	-1.47	0.15
MAP3K8	-2.31	0.15

CERK	-1.63	0.15
MTOR	-2.03	0.15
BRD7	-1.97	0.14
HK1	-0.71	0.14
PIP5K1B	-1.90	0.14
CSNK1A1	-0.24	0.14
PASK	-1.35	0.14
PIK3R4	-0.68	0.14
PBK	-1.23	0.14
DCK	-1.63	0.14
PIK3CB	-1.08	0.14
MELK	-0.20	0.14
SHPK	-0.65	0.14
CDK6	-1.69	0.14
PDK1	-1.42	0.14
MAK	-1.18	0.14
CDKN2A	-1.18	0.14
PI4KA	-0.34	0.13
GIT2	0.09	0.13
PKIG	-0.89	0.13
FUK	-1.09	0.13
PLAU	0.09	0.13
PIP5K1A	-0.88	0.13
CKM	0.39	0.12
NYAP2	-1.36	0.12
MAP2K5	-0.31	0.12
CALM1	1.04	0.12
ITPKB	-0.03	0.12
UCK1	-0.85	0.12

AURKA	-0.49	0.12
ULK3	0.90	0.12
TSSK3	-0.30	0.12
PRKAA2	-1.01	0.12
MARK1	-1.92	0.12
NME6	-0.72	0.11
RIPK3	-1.96	0.11
TPK1	-0.92	0.11
ACVRL1	-1.30	0.11
STK10	0.03	0.11
TRRAP	-1.07	0.11
ACVR1	1.16	0.11
MAPK8IP3	-1.20	0.11
PIK3C2B	-0.87	0.11
TRIB2	0.06	0.11
CDK2AP2	-1.10	0.11
GAK	0.86	0.11
PKD2	-0.55	0.10
MAP3K3	-2.31	0.10
ITK	-1.67	0.10
PRKAR2A	0.74	0.10
ACVR1C	-0.42	0.10
PLK3	-1.07	0.10
CCDC88A	-1.33	0.10
PNCK	-1.33	0.10
DGKI	0.23	0.10
CDKN2B	0.87	0.10
MYO3A	-1.17	0.10
GRK6	-0.04	0.10

CDK8	-0.73	0.10
TSSK2	-0.45	0.10
EPHX1	-1.75	0.10
BORA	-0.16	0.10
RBKS	-0.13	0.10
AK1	-0.28	0.10
FPGT	-1.03	0.10
ABL2	-0.13	0.10
DGKA	-0.54	0.09
SIK2	-0.28	0.09
GCK	-1.53	0.09
PHKG1	0.01	0.09
PRKCE	0.65	0.09
AKAP3	0.82	0.09
CDK5R2	0.11	0.09
MAPK13	2.10	0.09
SRPK1	-0.36	0.09
MVK	-0.75	0.09
AURKC	-0.84	0.09
HIPK2	-0.86	0.09
MLKL	-0.99	0.09
MADD	0.20	0.09
AMHR2	-0.78	0.09
GRK5	-0.65	0.09
PRKDC	-0.11	0.09
ERN1	-0.66	0.09
AK2	-0.75	0.09
AK2	-0.75	0.09
RPS6KA2	-0.77	0.09

ANKK1	-0.81	0.09
BAZ1B	-1.30	0.09
KIAA1804	-0.63	0.09
NUAK1	0.85	0.09
AK5	-1.36	0.09
AURKB	-0.83	0.09
PRKRA	0.37	0.08
DCLK2	-0.33	0.08
ADK	-0.28	0.08
RIOK1	-0.10	0.08
CCNO	-1.69	0.08
ADCK4	-0.21	0.08
CHD4	-0.62	0.08
MAPK4	-0.67	0.08
RPS6KA1	-0.74	0.08
STK17B	-0.93	0.08
HKDC1	-0.06	0.08
PLAUR	0.21	0.08
CIT	1.14	0.08
CHUK	0.52	0.08
ALPK2	-0.01	0.08
IKBKE	-1.23	0.08
SCYL2	0.06	0.08
ABL1	-0.26	0.08
PRKACA	-0.52	0.08
NEK10	-1.83	0.08
RIOK3	-0.27	0.08
DCLK3	0.32	0.08
BRAF	0.06	0.08

EIF2AK3	-0.69	0.08
SRM	0.53	0.07
MAP2K7	0.71	0.07
MOB3A	0.51	0.07
STK35	1.37	0.07
YES1	0.16	0.07
GK5	-1.63	0.07
PANK2	-1.34	0.07
SKAP1	-2.06	0.07
LATS1	-0.78	0.07
ADPGK	0.57	0.07
TGFBR2	-0.36	0.07
MOB3B	0.40	0.07
EIF2AK4	0.95	0.07
EGFR	0.11	0.07
HIPK3	-1.16	0.07
LRRK2	-1.05	0.07
TBCK	0.77	0.07
TRIO	0.23	0.07
DGKH	1.35	0.07
BCKDK	-0.08	0.07
COL4A3BP	1.64	0.07
PIK3C2A	0.52	0.07
BRDT	-0.21	0.07
PDPK1	0.47	0.07
EPHB2	0.38	0.07
GUCY2D	-0.73	0.07
SIK3	0.68	0.07
PCK2	-0.21	0.07

XYLB	0.68	0.06
KSR1	-1.06	0.06
TNIK	2.27	0.06
GKAP1	0.77	0.06
TGFBR1	0.88	0.06
PRKG1	2.93	0.06
AK3	-0.64	0.06
CDK5	-0.41	0.06
MAP4K2	-1.16	0.06
CHKA	-0.54	0.06
STRADA	0.72	0.06
CCL2	-0.29	0.06
GCKR	1.15	0.06
TBK1	0.73	0.06
CDC42BPG	-1.77	0.06
AK4	-1.64	0.06
STK4	0.23	0.06
NRBP1	1.58	0.06
VRK3	0.82	0.06
BRD4	-2.45	0.06
STK17A	0.01	0.06
IPMK	-0.19	0.06
ALPK3	-0.61	0.06
PRKD2	0.17	0.06
LIMK1	0.31	0.06
CDK17	0.80	0.06
ADRBK2	-0.05	0.06
CSNK1G3	0.39	0.06
AKAP12	-0.24	0.05

MAP3K15	-0.49	0.05
NLK	1.16	0.05
MASTL	0.87	0.05
CPNE3	0.36	0.05
IRAK1	1.16	0.05
GLYCTK	-0.53	0.05
PIK3CG	-0.27	0.05
CHEK1	-0.06	0.05
SCYL3	0.62	0.05
ROR1	0.74	0.05
GRK1	-0.01	0.05
TAB3	-0.29	0.05
OSR1	-0.13	0.05
PRKG2	0.07	0.05
STK19	-1.16	0.05
CDH1	-0.13	0.05
BMPR2	0.92	0.05
GRK4	1.37	0.05
TAOK3	0.32	0.05
AK8	1.00	0.05
PRKCH	1.24	0.05
TYRO3	0.24	0.05
ADCK5	-0.02	0.05
UHMK1	0.21	0.05
CDC42BPA	0.96	0.05
BAZ1A	-0.14	0.05
STK38	1.15	0.05
STK33	1.57	0.04
PRKCA	-0.30	0.04

MATK	0.06	0.04
GTF2F1	-1.69	0.04
DGUOK	-1.27	0.04
EPHA10	-0.48	0.04
CDKN1C	1.26	0.04
AKAP2	1.11	0.04
PSKH1	0.49	0.04
PRKCG	0.59	0.04
RPS6KB1	0.16	0.04
MYLK3	-0.63	0.04
CKMT2	-0.97	0.04
PIP4K2B	1.66	0.04
GUCY2F	0.23	0.04
MAP2K4	-0.39	0.04
TESK2	-0.06	0.04
AKAP17A	-0.36	0.04
MARK4	0.51	0.04
NME3	0.05	0.04
SBK1	-1.76	0.04
BUB1B	-0.86	0.04
TAF1	1.18	0.04
PRKD1	1.00	0.04
TP53RK	0.69	0.04
PI4KB	2.70	0.04
ACVR2A	0.39	0.04
CCR2	0.45	0.04
MKNK2	1.00	0.04
AKT3	2.38	0.04
ADCK1	1.36	0.04

CDK11B	0.29	0.03
EPHA7	0.58	0.03
BLVRA	0.11	0.03
PKDCC	1.08	0.03
CNKSR2	2.04	0.03
PCK1	-0.19	0.03
CDK10	0.42	0.03
BTK	0.88	0.03
MKNK1	0.15	0.03
AKD1	0.32	0.03
PNKP	0.36	0.03
CDK3	-0.45	0.03
HIPK1	-0.99	0.03
DCLK1	0.86	0.03
MAP3K11	0.51	0.03
CSNK1A1L	1.00	0.03
LRRK1	0.30	0.03
CDK12	-0.06	0.03
PINK1	0.36	0.03
DYRK3	0.65	0.03
NUCKS1	0.78	0.03
PI4K2A	-0.20	0.03
NME5	-0.63	0.03
AKAP7	1.26	0.03
FASTKD3	0.01	0.03
GALK2	1.09	0.03
RNASEL	-0.21	0.03
CDKL3	1.57	0.03
WEE1	0.32	0.03

PHKG2	0.34	0.03
CSNK2B	1.74	0.02
SETD5	0.26	0.02
PLK2	1.06	0.02
TRPM6	1.51	0.02
CKB	-0.65	0.02
OBSCN	0.59	0.02
PIK3IP1	1.02	0.02
MAP2K3	1.83	0.02
MAP3K12	-0.51	0.02
RPS6KA6	-0.45	0.02
ZAP70	-0.22	0.02
GUK1	-0.83	0.02
PPIP5K1	0.06	0.02
PHKA1	2.13	0.02
ALPK1	0.41	0.02
CDKL1	1.83	0.02
ACVR2B	0.24	0.02
AKAP10	-0.29	0.02
MAPKAP1	0.22	0.02
TEX14	0.23	0.02
PFKP	-0.53	0.02
SNRK	0.33	0.02
PRKAR1B	1.05	0.02
PGK2	-0.13	0.02
PLK1	1.23	0.02
JAK3	-0.27	0.02
MAPK15	0.25	0.02
PTK2B	1.32	0.02

CSNK1G1	0.41	0.02
TXNDC3	-0.28	0.01
LMTK3	0.45	0.01
PRKRIR	0.38	0.01
ETNK1	0.85	0.01
HGS	1.40	0.01
MAPK6	0.15	0.01
PRKAG1	0.17	0.01
EPHA4	1.21	0.01
ROCK2	-1.02	0.01
DGKG	1.46	0.01
MAPK1	0.47	0.01
CDK19	1.65	0.01
CDK19	1.65	0.01
MOB3C	1.96	0.01
IRAK2	1.53	0.01
PIK3R5	-1.71	0.01
SGK1	1.15	0.01
MARK2	1.01	0.01
BUB1	0.64	0.01
PIK3R6	1.19	0.01
ERN2	0.16	0.01
PIP4K2A	-0.73	0.01
HUS1	0.23	0.01
SYK	0.79	0.01
IPPK	-0.17	0.01
IP6K2	0.86	0.01
MAPKAPK3	1.05	0.01
SPHK1	0.41	0.01

PHKA2	-0.27	0.01
MAPK9	-0.42	0.01
CHKB	0.52	0.01
LRGUK	-0.20	0.01
SRPK3	1.02	0.01
PPIP5K2	2.86	0.01
TTK	-0.08	0.01
KNDC1	0.91	0.01
CDKN2D	0.17	0.00
MAGI1	0.95	0.00
PXK	0.53	0.00
PIM3	0.41	0.00
MAP3K7	-1.06	0.00
ADRBK1	1.02	0.00
PIK3R1	1.57	0.00
PAK1	1.54	0.00
HCK	-0.01	0.00
PANK1	1.18	0.00
ETNK2	0.16	0.00
DAPK2	0.70	0.00
ITPK1	0.14	0.00
NUAK2	0.63	0.00
PDK3	2.14	0.00
MAPK12	1.43	0.00
PIM2	0.78	0.00
DGKE	0.69	0.00
STK16	-0.73	0.00
PRKAR1A	0.68	0.00
MAPKBP1	-0.41	0.00

PRKAB2	2.04	0.00
FYN	-1.04	0.00
PRKACG	0.52	0.00
STRADB	-0.03	-0.01
PRKCZ	-2.33	-0.01
SMG1	0.66	-0.01
EPHA3	2.26	-0.01
RIPK1	0.12	-0.01
AKT1	0.07	-0.01
HSPB8	0.02	-0.01
WNK3	0.60	-0.01
BLK	0.16	-0.01
TK1	-0.75	-0.01
TTBK2	-0.17	-0.01
PACSIN2	0.88	-0.01
RPS6KL1	-0.71	-0.02
PIK3R2	0.02	-0.02
CSNK2A2	0.83	-0.02
CDKN2C	0.02	-0.02
ADCK2	1.14	-0.02
DGKK	0.68	-0.02
FGR	-1.14	-0.02
NRGN	-0.45	-0.02
MAP4K4	0.74	-0.02
CDK2AP1	0.15	-0.02
CDKN1B	-0.63	-0.02
CDKL5	-1.25	-0.02
MAPK1IP1L	0.67	-0.02
NAGK	-1.25	-0.02

STK32B	0.62	-0.02
MAPK8IP1	0.91	-0.02
TLK2	-0.31	-0.02
STK11	-1.47	-0.02
PFKFB1	0.61	-0.02
CAMKK1	-0.43	-0.02
TTN	0.26	-0.02
SGK2	-0.90	-0.03
CKMT1A	1.34	-0.03
CDK18	-0.85	-0.03
RNF2	0.08	-0.03
PRKCDBP	0.49	-0.04
MARCKS	0.07	-0.04
ITPKA	0.59	-0.04
MAST3	-0.39	-0.04
VRK1	0.80	-0.04
ABR	-1.76	-0.04
FN3K	1.74	-0.04
JAK2	-1.24	-0.04
IBTK	0.66	-0.04
NEK6	0.35	-0.04
PANK4	0.43	-0.04
MAPK8IP2	0.54	-0.05
GIT1	1.14	-0.05
MAST2	-1.90	-0.05
PSKH2	1.55	-0.05
EIF2AK1	0.07	-0.05
AKAP14	-1.35	-0.05
PRKX	0.93	-0.05

AKAP6	0.50	-0.05
STYK1	0.54	-0.05
MAP3K5	1.76	-0.05
TAOK2	-1.52	-0.05
NRK	1.41	-0.05
CSNK1E	1.12	-0.06
PFKM	0.36	-0.06
UCK2	-0.74	-0.06
MST1	0.64	-0.06
STK40	0.19	-0.06
HIPK4	0.40	-0.06
DYRK1B	0.47	-0.06
TXK	-1.89	-0.06
EPHB6	0.32	-0.06
RAF1	-1.80	-0.07
PAK7	-0.18	-0.07
HK3	0.02	-0.07
MAP2K2	0.37	-0.07
DMPK	-1.08	-0.07
CSNK1D	0.32	-0.07
STK24	-1.56	-0.07
CDKN1A	-1.69	-0.07
PKIB	0.07	-0.07
PIK3R3	0.76	-0.07
MAP3K10	-0.56	-0.07
GSK3A	-1.67	-0.07
SLK	0.41	-0.08
PRKAR2B	-2.39	-0.08
AKAP5	1.60	-0.08

FASTK	-0.54	-0.08
IRAK4	-0.06	-0.08
PLK1S1	0.01	-0.08
DAPK1	-1.22	-0.08
NPR2	0.55	-0.08
CSK	-1.60	-0.08
CAMK2A	-1.74	-0.08
PHKB	-0.29	-0.08
MYO3B	-0.01	-0.08
IRAK3	-1.51	-0.08
NRBP2	0.46	-0.08
MOS	-0.52	-0.09
MOB2	-0.47	-0.09
NADKD1	-0.70	-0.09
TBRG4	-1.54	-0.09
MAPK14	0.07	-0.09
MERTK	-0.52	-0.09
PIM1	1.74	-0.09
STK32C	-0.25	-0.09
CSNK1G2	-0.70	-0.09
MAP4K3	-0.08	-0.09
DGKQ	-1.72	-0.09
EIF2AK2	-2.07	-0.09
FXN	0.10	-0.10
ADCK3	-0.55	-0.10
PRKAG2	-0.39	-0.10
PIP5KL1	-1.85	-0.10
DYRK1A	-1.79	-0.10
PKD1	-1.56	-0.10

CKMT1B	-2.00	-0.10
FER	-1.65	-0.10
MAST4	-2.74	-0.10
KHK	0.95	-0.10
NEK3	-1.72	-0.10
SBK2	-0.91	-0.10
STK31	-1.45	-0.10
RPS6KA5	-0.02	-0.11
MAST1	-0.87	-0.11
ULK1	0.37	-0.11
IP6K1	-0.11	-0.11
PMVK	-0.84	-0.11
PKIA	-2.44	-0.11
TESK1	-0.56	-0.11
PRKAG3	-1.93	-0.11
CLK3	-1.28	-0.11
TAB2	-0.75	-0.11
PKN2	-1.83	-0.11
PAK4	-1.26	-0.11
AKAP9	-2.24	-0.12
PACSIN3	0.08	-0.12
FASTKD5	-0.71	-0.12
FASTKD1	0.41	-0.12
MYLK2	-2.02	-0.12
MYT1	-0.72	-0.12
MAPK7	-1.07	-0.12
MYLK4	-1.85	-0.12
FES	-0.79	-0.13
FASTKD2	-0.50	-0.13

TRPM7	-1.27	-0.13
NEK2	-1.25	-0.13
MAP3K9	-1.14	-0.13
KIDINS220	1.44	-0.14
DGKD	-1.43	-0.14
TAF1L	-0.73	-0.14
PDXK	-2.06	-0.14
PKM2	-2.39	-0.14
TNK2	-0.19	-0.14
RIPK2	-1.66	-0.14
AKAP13	-1.54	-0.14
NEK1	-0.69	-0.14
MAP3K13	-0.52	-0.14
AKAP8	-2.20	-0.14
PKN1	-2.67	-0.14
WNK2	-2.67	-0.14
GK	-1.69	-0.14
MAP3K4	-0.19	-0.15
CLK1	-2.20	-0.15
PKN1	-1.64	-0.15
IKBKAP	-2.44	-0.15
PDK4	-0.56	-0.15
DGKZ	-0.20	-0.15
MAGI3	-0.83	-0.16
DSTYK	-1.09	-0.16
KSR2	-1.23	-0.16
FGFRL1	-1.34	-0.16
FRK	-1.05	-0.17
MAPKAPK5	-1.60	-0.17

IP6K3	-0.37	-0.17
NADK	-1.74	-0.17
PRKAB1	-0.54	-0.18
ITPKC	-2.43	-0.18
FLT3LG	-1.38	-0.19
CDKL2	-1.45	-0.19
STK3	-0.89	-0.19
AKAP1	-2.23	-0.19
AKAP4	-1.35	-0.19
ACVR1B	-2.24	-0.20
PRKACB	-1.58	-0.20
MAP2K6	-1.26	-0.20
PIKFYVE	-1.41	-0.21
PKLR	-1.34	-0.22
MAP3K2	-1.18	-0.23
CSNK2A1	-1.33	-0.23
PANK3	-1.50	-0.24
MAPK11	-0.76	-0.24
KALRN	-1.59	-0.26
PIP4K2C	-1.43	-0.28
GSK3B	-1.03	-0.30
MAP3K14	-2.30	-0.31

Table S2. Clinical characteristics of stage III CRC patients from two cohorts

Characteristic	Training set (N=76)	Replication set (N=66)	P value
Age, median (range),years	60(24.0-79.0)	63(30.0-84.0)	0.06
Gender, n (%)			
male	44(57.9)	42(63.6)	
female	32(42.1)	24(36.4)	0.49
Stage (TNM), n (%)			
IIIa	9(11.8)	4(6.1)	
IIIb	57(75.0)	45(68.2)	
IIIc	10(13.2)	17(25.8)	0.11
Death, n (%)			
Yes	28(36.8)	15(22.7)	
No	48(63.2)	51(77.3)	0.07
Recurrence, n (%)			
Yes	19(25.0)	19(28.8)	
No	57(75.0)	47(71.2)	0.61
Median (range) follow-up time, months	57(4.0-79.0)	39(7.0-94.0)	0.002

Note: All the patients received 5FU together with oxaliplatin after surgical resection.

Table S3. Association analysis of relative copy number of 40 hit genes with recurrence

Variables	No recurrence mean(SD)	Recurrence mean(SD)	HR (95% CI)	P value	No recurrence mean(SD)	Recurrence mean(SD)	HR (95% CI)	P value	No recurrence mean(SD)	Recurrenc e mean(SD)	HR (95% CI)	P value
**Training set**					**Replication set**				**Pooled analysis**			
MAP4K1	1.84(1.83)	3.51(7.50)	1.15(1.05-1.26)	**0.002**	1.47(2.04)	2.46(4.18)	1.16(1.01-1.32)	**0.04**	1.67(1.92)	2.99(6.01)	1.14(1.06-1.22)	**0.0002**
CDKL4	1.08(0.54)	1.67(3.48)	1.20(1.02-1.40)	**0.02**	1.27(1.06)	1.47(1.39)	1.22(0.84-1.76)	0.29	1.17(0.82)	1.57(2.62)	1.17(1.02-1.35)	**0.03**
STK39	1.17(1.21)	1.26(1.26)	1.18(0.84-1.66)	0.33	1.35(1.75)	1.66(1.71)	1.10(0.88-1.38)	0.40	1.25(1.48)	1.46(1.49)	1.09(0.91-1.31)	0.33
PRKD3	3.54(7.48)	3.18(9.69)	0.99(0.92-1.06)	0.70	1.72(2.48)	1.62(2.22)	1.05(0.84-1.32)	0.67	2.72(5.83)	2.40(6.98)	1.00(0.93-1.06)	0.88
AGK	2.37(6.42)	1.51(2.34)	0.96(0.85-1.08)	0.49	1.75(4.03)	1.25(1.02)	0.94(0.76-1.16)	0.58	2.09(5.46)	1.38(1.79)	0.95(0.85-1.06)	0.34
PIK3CA	11.90(41.09)	3.05(5.96)	0.97(0.91-1.04)	0.38	2.26(5.92)	2.95(5.45)	1.01(0.95-1.07)	0.77	7.54(30.93)	3.00(5.63)	0.98(0.94-1.02)	0.36
PKN3	7.76(21.51)	2.12(4.43)	0.95(0.85-1.05)	0.30	4.66(18.86)	1.39(1.41)	0.96(0.81-1.13)	0.61	6.36(20.32)	1.76(3.26)	0.95(0.88-1.03)	0.23
HK2	1.54(1.77)	1.52(1.66)	1.01(0.77-1.33)	0.93	1.11(0.65)	1.02(0.34)	0.72(0.22-2.29)	0.58	1.34(1.40)	1.27(1.21)	0.98(0.75-1.29)	0.89
MST1R	1.36(0.90)	1.49(0.87)	1.53(0.89-2.64)	0.12	1.11(0.54)	1.05(0.51)	0.75(0.30-1.93)	0.56	1.25(0.77)	1.27(0.74)	1.21(0.78-1.88)	0.40
NEK8	2.30(3.11)	1.90(2.65)	0.88(0.71-1.09)	0.26	1.67(2.76)	1.10(0.74)	0.80(0.51-1.26)	0.34	2.01(2.96)	1.50(1.96)	0.89(0.74-1.06)	0.20
SPHK2	2.60(4.99)	1.19(1.08)	0.85(0.64-1.14)	0.28	1.20(0.90)	1.03(0.37)	0.67(0.31-1.46)	0.31	1.97(3.79)	1.11(0.80)	0.82(0.62-1.09)	0.18
DOLK	1.75(1.40)	2.08(3.45)	1.21(0.96-1.52)	0.11	1.34(1.95)	0.96(0.32)	0.52(0.16-1.66)	0.27	1.57(1.68)	1.52(2.48)	1.02(0.85-1.24)	0.80
BMPR1A	2.63(5.82)	1.06(0.87)	0.83(0.58-1.19)	0.31	1.09(0.42)	1.00(0.32)	0.54(0.15-1.95)	0.35	1.93(4.37)	1.03(0.65)	0.78(0.54-1.14)	0.21
NEK4	1.09(0.60)	0.97(0.46)	0.86(0.32-2.34)	0.77	1.09(0.62)	1.13(0.48)	0.87(0.43-1.73)	0.69	1.09(0.61)	1.05(0.47)	0.94(0.54-1.62)	0.81
PRKCD	2.13(3.27)	1.63(2.76)	0.95(0.79-1.13)	0.55	1.07(0.46)	1.00(0.17)	0.66(0.17-2.53)	0.54	1.65(2.49)	1.32(1.95)	0.94(0.78-1.13)	0.52
BMPR1B	2.22(5.21)	1.25(1.17)	0.89(0.68-1.18)	0.42	1.20(1.18)	0.96(0.21)	0.47(0.11-1.98)	0.31	1.76(3.95)	1.10(0.84)	0.86(0.62-1.18)	0.35
DYRK4	1.25(1.00)	1.63(2.66)	1.13(0.91-1.39)	0.26	1.16(0.58)	1.02(0.51)	0.58(0.22-1.52)	0.27	1.21(0.84)	1.32(1.91)	1.09(0.87-1.36)	0.44
LIMK2	2.12(3.65)	1.53(2.34)	0.94(0.77-1.16)	0.57	1.29(1.27)	0.99(0.45)	0.57(0.24-1.32)	0.19	1.74(2.85)	1.26(1.69)	0.89(0.71-1.12)	0.32
NME4	2.08(2.59)	1.61(1.95)	0.88(0.69-1.11)	0.28	8.83(47.25)	1.05(0.92)	0.78(0.48-1.25)	0.30	5.13(31.81)	1.33(1.53)	0.88(0.72-1.08)	0.22
LYN	2.25(4.45)	1.41(2.14)	0.92(0.77-1.10)	0.35	1.20(0.68)	1.05(0.47)	0.69(0.30-1.60)	0.39	1.77(3.36)	1.23(1.54)	0.92(0.76-1.11)	0.36
NEK9	2.98(4.17)	2.86(4.73)	0.97(0.86-1.08)	0.56	1.28(1.01)	1.28(1.42)	0.95(0.61-1.47)	0.81	2.21(3.26)	2.07(3.54)	0.97(0.87-1.08)	0.56
BMP2K	6.16(19.06)	1.89(2.73)	0.95(0.85-1.06)	0.33	1.21(0.80)	1.07(0.62)	0.67(0.33-1.38)	0.28	3.92(14.28)	1.48(1.99)	0.94(0.83-1.06)	0.30
AK7	1.42(1.02)	1.59(1.38)	1.07(0.75-1.54)	0.70	1.13(0.65)	1.23(0.88)	1.07(0.57-2.00)	0.84	1.29(0.88)	1.41(1.15)	1.12(0.82-1.52)	0.48
ULK4	1.37(0.92)	1.49(0.98)	1.15(0.75-1.78)	0.52	1.11(0.51)	1.07(0.41)	0.83(0.34-2.05)	0.69	1.25(0.77)	1.28(0.77)	1.10(0.74-1.64)	0.63
WNK1	1.31(1.39)	1.07(1.32)	0.76(0.42-1.38)	0.37	1.04(0.38)	1.12(0.37)	1.32(0.40-4.36)	0.65	1.19(1.06)	1.09(0.96)	0.89(0.59-1.34)	0.57
CDC7	1.75(2.98)	3.83(9.18)	1.02(0.96-1.08)	0.53	1.06(0.42)	1.22(0.69)	1.76(0.73-4.27)	0.21	1.44(2.24)	2.52(6.56)	1.03(0.97-1.08)	0.32
RIOK2	1.81(3.74)	6.69(22.33)	1.01(0.99-1.04)	0.21	1.24(1.36)	4.71(15.58)	1.03(0.99-1.06)	0.10	1.55(2.92)	5.70(19.02)	1.02(1.00-1.03)	0.07
DGKB	1.87(2.37)	1.67(1.92)	0.93(0.73-1.19)	0.57	2.18(6.05)	1.08(0.75)	0.78(0.47-1.28)	0.32	2.01(4.40)	1.38(1.47)	0.90(0.74-1.11)	0.34
PTK2	1.25(0.89)	1.43(2.40)	1.28(0.90-1.81)	0.17	1.13(0.58)	1.03(0.40)	0.75(0.27-2.12)	0.59	1.20(0.76)	1.23(1.71)	1.15(0.80-1.65)	0.45
TRIB1	2.15(5.76)	1.32(1.35)	0.95(0.81-1.12)	0.55	1.20(0.66)	1.07(0.66)	0.74(0.31-1.74)	0.49	1.71(4.26)	1.20(1.06)	0.95(0.80-1.13)	0.56
PGK1	1.78(1.84)	1.42(1.07)	0.85(0.54-1.33)	0.47	1.40(2.38)	1.10(0.51)	0.90(0.58-1.39)	0.64	1.61(2.10)	1.26(0.84)	0.88(0.62-1.24)	0.47
STK32A	1.96(1.76)	2.77(4.48)	1.21(1.03-1.43)	0.02	1.95(3.26)	1.29(0.85)	0.84(0.57-1.24)	0.38	1.95(2.54)	2.03(3.27)	1.03(0.92-1.15)	0.60
PRKCB	1.29(1.00)	1.47(1.01)	1.13(0.77-1.66)	0.52	1.56(2.88)	1.03(0.60)	0.72(0.36-1.42)	0.34	1.41(2.07)	1.25(0.85)	0.94(0.72-1.22)	0.63
MYLK	2.62(4.44)	3.32(5.77)	1.03(0.94-1.13)	0.53	1.18(0.68)	0.95(0.36)	0.45(0.14-1.45)	0.18	1.97(3.38)	2.13(4.20)	1.02(0.93-1.12)	0.68
PI4K2B	66.04(326.65)	11.04(22.40)	1.00(0.99-1.01)	0.62	1.14(0.54)	1.03(0.47)	0.77(0.26-2.23)	0.63	36.71(243.03)	6.04(16.43)	1.00(0.99-1.01)	0.61
MAP2K1	1.86(3.27)	1.85(2.92)	1.03(0.86-1.24)	0.71	1.73(3.61)	1.04(0.57)	0.73(0.41-1.30)	0.28	1.80(3.41)	1.44(2.11)	0.95(0.80-1.13)	0.56
SGK3	3.85(11.72)	2.45(5.27)	0.98(0.93-1.04)	0.56	1.11(0.62)	1.06(0.36)	0.79(0.30-2.07)	0.63	2.61(8.76)	1.75(3.75)	0.98(0.93-1.05)	0.60
MAGI2	1.94(3.23)	0.90(0.78)	0.70(0.44-1.11)	0.13	7.45(35.23)	0.85(0.31)	0.53(0.14-2.02)	0.36	4.43(23.82)	0.87(0.58)	0.69(0.43-1.10)	0.12
CERKL	1.20(0.62)	1.55(1.11)	1.32(0.87-2.00)	0.19	1.07(0.37)	1.00(0.29)	0.67(0.17-2.71)	0.58	1.14(0.53)	1.28(0.84)	1.27(0.83-1.95)	0.26
CDK4	1.83(2.61)	2.07(2.20)	1.04(0.87-1.23)	0.69	1.00(0.43)	9.72(38.33)	1.01(1.00-1.03)	0.09	1.46(1.99)	5.89(27.06)	1.01(1.00-1.03)	0.04

Table S4. Association analysis of relative copy number of 40 hit genes with survival

Gene symbol	Alive mean(SD)	Dead mean(SD)	HR (95% CI)	P value	Alive mean(SD)	Dead mean(SD)	HR (95% CI)	P value	Alive mean(SD)	Dead mean(SD)	HR (95% CI)	P value
**Training set**					**Replication set**				**Pooled analysis**			
MAP4K1	1.74(1.75)	3.15(6.27)	1.11(1.03-1.19)	**0.01**	1.44(1.96)	2.83(4.66)	1.20(1.04-1.38)	**0.01**	1.59(1.86)	3.04(5.70)	1.11(1.05-1.18)	**0.0005**
CDKL4	0.99(0.36)	1.63(2.90)	1.14(1.00-1.29)	**0.05**	1.19(1.04)	1.80(1.43)	1.53(1.06-2.19)	**0.02**	1.09(0.79)	1.69(2.47)	1.15(1.03-1.28)	**0.01**
STK39	1.11(1.17)	1.33(1.31)	1.16(0.89-1.52)	0.28	1.27(1.70)	2.01(1.79)	1.22(0.97-1.54)	0.09	1.19(1.46)	1.58(1.51)	1.17(0.99-1.37)	0.06
PRKD3	3.31(7.07)	3.68(9.57)	1.00(0.96-1.05)	0.95	1.60(2.39)	2.01(2.42)	1.21(0.95-1.54)	0.12	2.43(5.26)	3.10(7.84)	1.01(0.96-1.05)	0.76
AGK	2.65(6.96)	1.30(1.98)	0.93(0.79-1.09)	0.36	1.65(3.88)	1.47(1.09)	0.97(0.83-1.14)	0.71	2.13(5.58)	1.36(1.71)	0.95(0.85-1.06)	0.36
PIK3CA	8.61(25.67)	11.52(49.13)	1.00(0.99-1.01)	0.69	2.13(5.70)	3.57(6.01)	1.02(0.96-1.08)	0.59	5.27(18.53)	8.75(39.73)	1.00(0.99-1.01)	0.76
PKN3	8.29(23.30)	3.03(5.09)	0.98(0.93-1.02)	0.29	4.34(18.12)	1.62(1.52)	0.97(0.88-1.08)	0.61	6.25(20.78)	2.54(4.23)	0.98(0.94-1.02)	0.23
HK2	1.59(1.80)	1.44(1.64)	0.94(0.74-1.20)	0.62	1.10(0.64)	1.01(0.28)	0.74(0.19-2.83)	0.65	1.34(1.35)	1.29(1.34)	0.93(0.73-1.19)	0.56
MST1R	1.30(0.74)	1.54(1.09)	1.50(0.97-2.30)	0.07	1.06(0.54)	1.21(0.51)	1.34(0.58-3.09)	0.50	1.18(0.65)	1.42(0.94)	1.39(0.97-2.00)	0.07
NEK8	2.39(2.92)	1.88(3.12)	0.94(0.80-1.09)	0.41	1.62(2.66)	1.12(0.80)	0.83(0.50-1.36)	0.46	1.99(2.80)	1.62(2.57)	0.92(0.8-1.06)	0.26
SPHK2	2.63(5.30)	1.60(1.96)	0.94(0.81-1.08)	0.35	1.16(0.87)	1.12(0.40)	0.92(0.45-1.90)	0.83	1.87(3.79)	1.43(1.61)	0.93(0.81-1.07)	0.31
DOLK	1.66(1.30)	2.14(2.99)	1.19(1.00-1.41)	**0.04**	1.30(1.88)	1.00(0.36)	0.76(0.26-2.21)	0.61	1.47(1.63)	1.74(2.47)	1.07(0.92-1.24)	0.36
BMPR1A	2.78(6.30)	1.30(1.22)	0.89(0.72-1.10)	0.30	1.11(0.41)	0.93(0.27)	0.38(0.08-1.92)	0.24	1.92(4.46)	1.17(1.01)	0.86(0.68-1.10)	0.23
NEK4	1.05(0.50)	1.07(0.67)	1.21(0.61-2.39)	0.58	1.07(0.60)	1.20(0.51)	1.03(0.51-2.05)	0.94	1.06(0.56)	1.12(0.62)	1.18(0.73-1.89)	0.50
PRKCD	2.18(3.54)	1.69(2.33)	0.95(0.82-1.10)	0.46	1.06(0.44)	1.00(0.17)	0.71(0.15-3.47)	0.68	1.61(2.53)	1.45(1.90)	0.95(0.82-1.10)	0.46
BMPR1B	2.40(5.66)	1.24(1.03)	0.89(0.69-1.14)	0.34	1.20(1.13)	0.90(0.22)	0.33(0.06-1.90)	0.22	1.78(4.05)	1.12(0.85)	0.85(0.64-1.14)	0.28
DYRK4	1.20(0.91)	1.58(2.31)	1.06(0.89-1.27)	0.50	1.08(0.55)	1.25(0.62)	1.36(0.58-3.21)	0.48	1.14(0.74)	1.47(1.90)	1.10(0.93-1.31)	0.25
LIMK2	2.14(3.85)	1.69(2.36)	0.96(0.82-1.11)	0.55	1.23(1.23)	1.12(0.48)	0.85(0.42-1.72)	0.65	1.67(2.85)	1.49(1.93)	0.95(0.82-1.10)	0.48
NME4	2.06(2.56)	1.79(2.26)	0.96(0.81-1.14)	0.64	8.22(45.37)	1.05(1.01)	0.80(0.47-1.37)	0.43	5.23(32.60)	1.53(1.93)	0.94(0.81-1.09)	0.39
LYN	2.43(4.80)	1.36(1.91)	0.91(0.77-1.07)	0.23	1.19(0.62)	1.03(0.65)	0.73(0.28-1.90)	0.52	1.79(3.41)	1.24(1.58)	0.90(0.76-1.08)	0.26
NEK9	3.22(4.48)	2.49(3.97)	0.96(0.86-1.06)	0.38	1.24(0.97)	1.41(1.60)	1.09(0.71-1.67)	0.69	2.20(3.33)	2.12(3.36)	0.96(0.87-1.06)	0.37
BMP2K	6.67(20.55)	2.37(4.58)	0.96(0.90-1.03)	0.31	1.20(0.79)	1.07(0.63)	0.75(0.34-1.67)	0.48	3.86(14.50)	1.92(3.74)	0.96(0.90-1.03)	0.30
AK7	1.57(1.19)	1.28(0.96)	0.85(0.58-1.23)	0.38	1.07(0.62)	1.46(0.94)	1.58(0.89-2.82)	0.12	1.31(0.97)	1.34(0.95)	0.99(0.73-1.34)	0.94
ULK4	1.50(0.99)	1.22(0.80)	0.79(0.50-1.25)	0.32	1.11(0.47)	1.04(0.52)	0.81(0.28-2.36)	0.70	1.30(0.79)	1.16(0.71)	0.77(0.50-1.19)	0.24
WNK1	1.37(1.49)	1.04(1.12)	0.78(0.49-1.24)	0.30	1.04(0.36)	1.12(0.43)	1.61(0.41-6.34)	0.49	1.20(1.08)	1.07(0.93)	0.85(0.57-1.24)	0.39
CDC7	2.72(6.38)	1.51(2.25)	0.93(0.80-1.08)	0.35	1.13(0.45)	1.01(0.69)	0.77(0.24-2.46)	0.66	1.90(4.50)	1.34(1.86)	0.92(0.78-1.09)	0.35
RIOK2	1.86(4.07)	5.04(18.40)	1.01(0.99-1.03)	0.39	1.21(1.31)	5.73(17.52)	1.03(0.99-1.06)	0.11	1.52(2.99)	5.28(17.89)	1.01(1.00-1.03)	0.16
DGKB	1.86(2.18)	1.76(2.42)	0.98(0.82-1.18)	0.85	2.16(5.80)	0.85(0.58)	0.51(0.22-1.18)	0.12	2.01(4.41)	1.44(2.02)	0.92(0.77-1.10)	0.34
PTK2	1.27(0.88)	1.35(2.04)	1.14(0.84-1.54)	0.41	1.09(0.56)	1.13(0.45)	1.17(0.44-3.08)	0.75	1.18(0.74)	1.27(1.66)	1.12(0.84-1.50)	0.45
TRIB1	2.45(6.21)	1.04(0.98)	0.87(0.65-1.17)	0.36	1.26(0.69)	0.81(0.40)	0.16(0.03-0.8)	0.02	1.83(4.34)	0.96(0.82)	0.74(0.47-1.17)	0.20
PGK1	1.62(1.23)	1.81(2.28)	1.18(0.92-1.53)	0.20	1.37(2.29)	1.12(0.50)	0.91(0.61-1.35)	0.63	1.49(1.85)	1.57(1.88)	1.04(0.88-1.24)	0.64
STK32A	2.10(1.97)	2.27(3.65)	1.08(0.93-1.27)	0.32	1.92(3.15)	1.22(0.68)	0.78(0.48-1.28)	0.34	2.01(2.64)	1.90(2.99)	1.00(0.88-1.14)	0.97
PRKCB	1.39(0.93)	1.25(1.12)	0.91(0.60-1.38)	0.65	1.50(2.77)	1.07(0.62)	0.82(0.42-1.58)	0.56	1.45(2.08)	1.19(0.97)	0.88(0.63-1.22)	0.44
MYLK	2.63(4.55)	3.07(5.21)	1.00(0.93-1.08)	0.97	1.13(0.67)	1.07(0.37)	0.79(0.31-2.02)	0.62	1.86(3.28)	2.37(4.29)	1.01(0.93-1.09)	0.90
PI4K2B	65.39(347.99)	29.83(107.70)	1.00(1.00-1.00)	0.58	1.19(0.54)	0.84(0.35)	0.18(0.03-1.01)	0.05	32.32(243.14)	19.72(87.48)	1.00(1.00-1.00)	0.61
MAP2K1	1.60(1.63)	2.30(4.79)	1.06(0.96-1.18)	0.23	1.69(3.47)	0.97(0.58)	0.65(0.32-1.31)	0.23	1.65(2.72)	1.84(3.91)	1.01(0.91-1.11)	0.90
SGK3	4.30(12.73)	2.13(4.40)	0.98(0.92-1.04)	0.42	1.13(0.61)	0.99(0.26)	0.55(0.16-1.93)	0.35	2.67(8.97)	1.73(3.57)	0.98(0.92-1.04)	0.45
MAGI2	2.01(3.29)	1.10(1.79)	0.87(0.68-1.10)	0.23	6.92(33.84)	0.91(0.34)	0.85(0.27-2.70)	0.79	4.54(24.40)	1.03(1.45)	0.87(0.69-1.10)	0.23
CERKL	1.38(0.85)	1.13(0.62)	0.74(0.43-1.27)	0.28	1.04(0.37)	1.09(0.26)	1.69(0.40-7.17)	0.47	1.20(0.67)	1.12(0.52)	0.81(0.49-1.35)	0.42
CDK4	1.96(2.43)	1.77(2.66)	0.98(0.83-1.15)	0.78	1.00(0.42)	12.05(43.14)	1.01(1.00-1.02)	0.12	1.46(1.78)	5.36(25.48)	1.01(1.00-1.03)	0.06

Table S5. Intra group variance of two-gene ratios

Gene N (numerator)	Gene D (denominator)	Detector	Intra-group-variability
MAP4K1	*CDKL4*	v_1_2	14.51
MAP4K1	*STK39*	v_1_3	3.83
MAP4K1	*PRKD3*	v_1_4	19.33
MAP4K1	*AGK*	v_1_5	14.27
MAP4K1	*PIK3CA*	v_1_6	105.40
MAP4K1	*PKN3*	v_1_7	45.90
MAP4K1	*HK2*	v_1_8	41.84
MAP4K1	*MST1R*	v_1_9	10.53
MAP4K1	*NEK8*	v_1_10	77.21
MAP4K1	*SPHK2*	v_1_11	13.10
MAP4K1	*DOLK*	v_1_12	5.80
MAP4K1	*BMPR1A*	v_1_13	21.24
MAP4K1	*NEK4*	v_1_14	5.07
MAP4K1	*PRKCD*	v_1_15	35.03
MAP4K1	*BMPR1B*	v_1_16	14.55
MAP4K1	*DYRK4*	v_1_17	13.47
MAP4K1	*LIMK2*	v_1_18	18.24
MAP4K1	*NME4*	v_1_19	90.92
MAP4K1	*LYN*	v_1_20	141.91
MAP4K1	*NEK9*	v_1_21	21.52
MAP4K1	*BMP2K*	v_1_22	171.54
MAP4K1	*AK7*	v_1_23	11.08
MAP4K1	*ULK4*	v_1_24	73.86
MAP4K1	*WNK1*	v_1_25	19.16
MAP4K1	*CDC7*	v_1_26	60.04

MAP4K1	*RIOK2*	v_1_27	7.16
MAP4K1	*DGKB*	v_1_28	62.33
MAP4K1	*PTK2*	v_1_29	7.10
MAP4K1	*TRIB1*	v_1_30	44.72
MAP4K1	*PGK1*	v_1_31	14.50
MAP4K1	*STK32A*	v_1_32	13.91
MAP4K1	*PRKCB*	v_1_33	36.82
MAP4K1	*MYLK*	v_1_34	66.90
MAP4K1	*PI4K2B*	v_1_35	224.16
MAP4K1	*MAP2K1*	v_1_36	74.53
MAP4K1	*SGK3*	v_1_37	17.34
MAP4K1	*MAGI2*	v_1_38	13.95
MAP4K1	*CERKL*	v_1_39	22.41
MAP4K1	*CDK4*	v_1_40	27.10
CDKL4	*STK39*	v_2_3	0.16
CDKL4	*PRKD3*	v_2_4	1.59
CDKL4	*AGK*	v_2_5	0.77
CDKL4	*PIK3CA*	v_2_6	9.00
CDKL4	*PKN3*	v_2_7	8.36
CDKL4	*HK2*	v_2_8	31.45
CDKL4	*MST1R*	v_2_9	3.95
CDKL4	*NEK8*	v_2_10	9.33
CDKL4	*SPHK2*	v_2_11	3.52
CDKL4	*DOLK*	v_2_12	206.48
CDKL4	*BMPR1A*	v_2_13	5.26
CDKL4	*NEK4*	v_2_14	1.78
CDKL4	*PRKCD*	v_2_15	33.95
CDKL4	*BMPR1B*	v_2_16	5.96
CDKL4	*DYRK4*	v_2_17	1.32

CDKL4	*LIMK2*	v_2_18	7.74
CDKL4	*NME4*	v_2_19	10.60
CDKL4	*LYN*	v_2_20	31.60
CDKL4	*NEK9*	v_2_21	92.38
CDKL4	*BMP2K*	v_2_22	31.15
CDKL4	*AK7*	v_2_23	3.84
CDKL4	*ULK4*	v_2_24	35.28
CDKL4	*WNK1*	v_2_25	1.20
CDKL4	*CDC7*	v_2_26	5.47
CDKL4	*RIOK2*	v_2_27	2184.88
CDKL4	*DGKB*	v_2_28	10.99
CDKL4	*PTK2*	v_2_29	1.30
CDKL4	*TRIB1*	v_2_30	5.42
CDKL4	*PGK1*	v_2_31	1004.04
CDKL4	*STK32A*	v_2_32	185.24
CDKL4	*PRKCB*	v_2_33	12.16
CDKL4	*MYLK*	v_2_34	7.67
CDKL4	*PI4K2B*	v_2_35	105.84
CDKL4	*MAP2K1*	v_2_36	7.85
CDKL4	*SGK3*	v_2_37	1.09
CDKL4	*MAGI2*	v_2_38	1.78
CDKL4	*CERKL*	v_2_39	2.48
CDKL4	*CDK4*	v_2_40	15.89
STK39	*PRKD3*	v_3_4	1.00
STK39	*AGK*	v_3_5	0.66
STK39	*PIK3CA*	v_3_6	19.14
STK39	*PKN3*	v_3_7	6.22
STK39	*HK2*	v_3_8	15.17
STK39	*MST1R*	v_3_9	2.59

STK39	*NEK8*	v_3_10	6.24
STK39	*SPHK2*	v_3_11	4.30
STK39	*DOLK*	v_3_12	25.48
STK39	*BMPR1A*	v_3_13	7.43
STK39	*NEK4*	v_3_14	1.15
STK39	*PRKCD*	v_3_15	18.09
STK39	*BMPR1B*	v_3_16	4.01
STK39	*DYRK4*	v_3_17	2.02
STK39	*LIMK2*	v_3_18	4.31
STK39	*NME4*	v_3_19	15.30
STK39	*LYN*	v_3_20	15.30
STK39	*NEK9*	v_3_21	91.10
STK39	*BMP2K*	v_3_22	21.35
STK39	*AK7*	v_3_23	2.57
STK39	*ULK4*	v_3_24	19.16
STK39	*WNK1*	v_3_25	2.35
STK39	*CDC7*	v_3_26	8.92
STK39	*RIOK2*	v_3_27	10.38
STK39	*DGKB*	v_3_28	13.62
STK39	*PTK2*	v_3_29	1.13
STK39	*TRIB1*	v_3_30	9.35
STK39	*PGK1*	v_3_31	117.18
STK39	*STK32A*	v_3_32	119.89
STK39	*PRKCB*	v_3_33	10.56
STK39	*MYLK*	v_3_34	7.59
STK39	*PI4K2B*	v_3_35	73.43
STK39	*MAP2K1*	v_3_36	8.71
STK39	*SGK3*	v_3_37	1.31
STK39	*MAGI2*	v_3_38	3.13

STK39	*CERKL*	v_3_39	2.56
STK39	*CDK4*	v_3_40	7.50
PRKD3	*AGK*	v_4_5	5.32
PRKD3	*PIK3CA*	v_4_6	210.57
PRKD3	*PKN3*	v_4_7	26.86
PRKD3	*HK2*	v_4_8	106.94
PRKD3	*MST1R*	v_4_9	154.22
PRKD3	*NEK8*	v_4_10	45.74
PRKD3	*SPHK2*	v_4_11	31.49
PRKD3	*DOLK*	v_4_12	114485.69
PRKD3	*BMPR1A*	v_4_13	49.13
PRKD3	*NEK4*	v_4_14	16.06
PRKD3	*PRKCD*	v_4_15	46.16
PRKD3	*BMPR1B*	v_4_16	49.46
PRKD3	*DYRK4*	v_4_17	11.41
PRKD3	*LIMK2*	v_4_18	64.40
PRKD3	*NME4*	v_4_19	48.16
PRKD3	*LYN*	v_4_20	19.60
PRKD3	*NEK9*	v_4_21	6900.71
PRKD3	*BMP2K*	v_4_22	178.21
PRKD3	*AK7*	v_4_23	48.79
PRKD3	*ULK4*	v_4_24	2010.65
PRKD3	*WNK1*	v_4_25	8.87
PRKD3	*CDC7*	v_4_26	35.86
PRKD3	*RIOK2*	v_4_27	1238890.10
PRKD3	*DGKB*	v_4_28	397.03
PRKD3	*PTK2*	v_4_29	15.82
PRKD3	*TRIB1*	v_4_30	34.40
PRKD3	*PGK1*	v_4_31	564335.01

PRKD3	*STK32A*	v_4_32	96913.47
PRKD3	*PRKCB*	v_4_33	425.17
PRKD3	*MYLK*	v_4_34	50.56
PRKD3	*PI4K2B*	v_4_35	888.24
PRKD3	*MAP2K1*	v_4_36	37.80
PRKD3	*SGK3*	v_4_37	3.81
PRKD3	*MAGI2*	v_4_38	24.40
PRKD3	*CERKL*	v_4_39	62.00
PRKD3	*CDK4*	v_4_40	161.20
AGK	*PIK3CA*	v_5_6	14.64
AGK	*PKN3*	v_5_7	19.17
AGK	*HK2*	v_5_8	47.94
AGK	*MST1R*	v_5_9	33.53
AGK	*NEK8*	v_5_10	19.01
AGK	*SPHK2*	v_5_11	17.70
AGK	*DOLK*	v_5_12	147040.31
AGK	*BMPR1A*	v_5_13	26.01
AGK	*NEK4*	v_5_14	16.50
AGK	*PRKCD*	v_5_15	57.06
AGK	*BMPR1B*	v_5_16	31.17
AGK	*DYRK4*	v_5_17	5.95
AGK	*LIMK2*	v_5_18	6.94
AGK	*NME4*	v_5_19	37.41
AGK	*LYN*	v_5_20	25.94
AGK	*NEK9*	v_5_21	8890.00
AGK	*BMP2K*	v_5_22	46.37
AGK	*AK7*	v_5_23	31.56
AGK	*ULK4*	v_5_24	72.14
AGK	*WNK1*	v_5_25	18.09

AGK	*CDC7*	v_5_26	77.58
AGK	*RIOK2*	v_5_27	1591365.60
AGK	*DGKB*	v_5_28	46.38
AGK	*PTK2*	v_5_29	5.93
AGK	*TRIB1*	v_5_30	32.71
AGK	*PGK1*	v_5_31	725021.93
AGK	*STK32A*	v_5_32	124628.64
AGK	*PRKCB*	v_5_33	16.80
AGK	*MYLK*	v_5_34	8.45
AGK	*PI4K2B*	v_5_35	148.00
AGK	*MAP2K1*	v_5_36	11.50
AGK	*SGK3*	v_5_37	8.45
AGK	*MAGI2*	v_5_38	8.21
AGK	*CERKL*	v_5_39	27.26
AGK	*CDK4*	v_5_40	21.19
PIK3CA	*PKN3*	v_6_7	43221.06
PIK3CA	*HK2*	v_6_8	366.20
PIK3CA	*MST1R*	v_6_9	126.41
PIK3CA	*NEK8*	v_6_10	37.56
PIK3CA	*SPHK2*	v_6_11	153.68
PIK3CA	*DOLK*	v_6_12	1253.85
PIK3CA	*BMPR1A*	v_6_13	294.22
PIK3CA	*NEK4*	v_6_14	1035.46
PIK3CA	*PRKCD*	v_6_15	282.92
PIK3CA	*BMPR1B*	v_6_16	1067.63
PIK3CA	*DYRK4*	v_6_17	1865.08
PIK3CA	*LIMK2*	v_6_18	159.26
PIK3CA	*NME4*	v_6_19	203.75
PIK3CA	*LYN*	v_6_20	1558.32

PIK3CA	*NEK9*	v_6_21	6429.37
PIK3CA	*BMP2K*	v_6_22	726.39
PIK3CA	*AK7*	v_6_23	322.79
PIK3CA	*ULK4*	v_6_24	371.79
PIK3CA	*WNK1*	v_6_25	545.55
PIK3CA	*CDC7*	v_6_26	353.40
PIK3CA	*RIOK2*	v_6_27	9657.58
PIK3CA	*DGKB*	v_6_28	153.93
PIK3CA	*PTK2*	v_6_29	713.86
PIK3CA	*TRIB1*	v_6_30	448.96
PIK3CA	*PGK1*	v_6_31	4673.00
PIK3CA	*STK32A*	v_6_32	1528.01
PIK3CA	*PRKCB*	v_6_33	185.46
PIK3CA	*MYLK*	v_6_34	245.21
PIK3CA	*PI4K2B*	v_6_35	520.56
PIK3CA	*MAP2K1*	v_6_36	67.72
PIK3CA	*SGK3*	v_6_37	802.17
PIK3CA	*MAGI2*	v_6_38	4084.96
PIK3CA	*CERKL*	v_6_39	845.26
PIK3CA	*CDK4*	v_6_40	2413.90
PKN3	*HK2*	v_7_8	844.90
PKN3	*MST1R*	v_7_9	753.61
PKN3	*NEK8*	v_7_10	302.68
PKN3	*SPHK2*	v_7_11	377.26
PKN3	*DOLK*	v_7_12	2988.50
PKN3	*BMPR1A*	v_7_13	560.85
PKN3	*NEK4*	v_7_14	574.69
PKN3	*PRKCD*	v_7_15	498.20
PKN3	*BMPR1B*	v_7_16	399.40

PKN3	*DYRK4*	v_7_17	696.88
PKN3	*LIMK2*	v_7_18	79.61
PKN3	*NME4*	v_7_19	900.41
PKN3	*LYN*	v_7_20	443.71
PKN3	*NEK9*	v_7_21	7537.77
PKN3	*BMP2K*	v_7_22	622.00
PKN3	*AK7*	v_7_23	451.87
PKN3	*ULK4*	v_7_24	2506.35
PKN3	*WNK1*	v_7_25	646.00
PKN3	*CDC7*	v_7_26	1507.57
PKN3	*RIOK2*	v_7_27	6161.32
PKN3	*DGKB*	v_7_28	950.38
PKN3	*PTK2*	v_7_29	195.01
PKN3	*TRIB1*	v_7_30	1234.33
PKN3	*PGK1*	v_7_31	2705.66
PKN3	*STK32A*	v_7_32	2169.21
PKN3	*PRKCB*	v_7_33	354.75
PKN3	*MYLK*	v_7_34	191.95
PKN3	*PI4K2B*	v_7_35	851.08
PKN3	*MAP2K1*	v_7_36	695.11
PKN3	*SGK3*	v_7_37	1358.05
PKN3	*MAGI2*	v_7_38	330.38
PKN3	*CERKL*	v_7_39	822.51
PKN3	*CDK4*	v_7_40	629.08
HK2	*MST1R*	v_8_9	10.73
HK2	*NEK8*	v_8_10	20.58
HK2	*SPHK2*	v_8_11	0.86
HK2	*DOLK*	v_8_12	1132.36
HK2	*BMPR1A*	v_8_13	0.46

HK2	*NEK4*	v_8_14	4.93
HK2	*PRKCD*	v_8_15	0.31
HK2	*BMPR1B*	v_8_16	0.76
HK2	*DYRK4*	v_8_17	3.29
HK2	*LIMK2*	v_8_18	0.54
HK2	*NME4*	v_8_19	11.43
HK2	*LYN*	v_8_20	35.70
HK2	*NEK9*	v_8_21	102.46
HK2	*BMP2K*	v_8_22	0.51
HK2	*AK7*	v_8_23	12.67
HK2	*ULK4*	v_8_24	46.91
HK2	*WNK1*	v_8_25	4.06
HK2	*CDC7*	v_8_26	8.82
HK2	*RIOK2*	v_8_27	12221.83
HK2	*DGKB*	v_8_28	19.95
HK2	*PTK2*	v_8_29	3.36
HK2	*TRIB1*	v_8_30	6.40
HK2	*PGK1*	v_8_31	5622.38
HK2	*STK32A*	v_8_32	991.34
HK2	*PRKCB*	v_8_33	61.18
HK2	*MYLK*	v_8_34	1.15
HK2	*PI4K2B*	v_8_35	1.76
HK2	*MAP2K1*	v_8_36	21.64
HK2	*SGK3*	v_8_37	6.61
HK2	*MAGI2*	v_8_38	9.54
HK2	*CERKL*	v_8_39	29.89
HK2	*CDK4*	v_8_40	35.59
MST1R	*NEK8*	v_9_10	13.92
MST1R	*SPHK2*	v_9_11	1.78

MST1R	*DOLK*	v_9_12	63.30
MST1R	*BMPR1A*	v_9_13	1.85
MST1R	*NEK4*	v_9_14	0.89
MST1R	*PRKCD*	v_9_15	110.83
MST1R	*BMPR1B*	v_9_16	4.45
MST1R	*DYRK4*	v_9_17	0.98
MST1R	*LIMK2*	v_9_18	4.15
MST1R	*NME4*	v_9_19	3.02
MST1R	*LYN*	v_9_20	32.76
MST1R	*NEK9*	v_9_21	399.56
MST1R	*BMP2K*	v_9_22	54.82
MST1R	*AK7*	v_9_23	0.28
MST1R	*ULK4*	v_9_24	0.66
MST1R	*WNK1*	v_9_25	0.87
MST1R	*CDC7*	v_9_26	0.95
MST1R	*RIOK2*	v_9_27	612.22
MST1R	*DGKB*	v_9_28	3.01
MST1R	*PTK2*	v_9_29	0.94
MST1R	*TRIB1*	v_9_30	1.82
MST1R	*PGK1*	v_9_31	350.13
MST1R	*STK32A*	v_9_32	105.12
MST1R	*PRKCB*	v_9_33	11.38
MST1R	*MYLK*	v_9_34	9.48
MST1R	*PI4K2B*	v_9_35	316.23
MST1R	*MAP2K1*	v_9_36	1.77
MST1R	*SGK3*	v_9_37	2.33
MST1R	*MAGI2*	v_9_38	2.51
MST1R	*CERKL*	v_9_39	0.68
MST1R	*CDK4*	v_9_40	0.95

NEK8	*SPHK2*	v_10_11	13.80
NEK8	*DOLK*	v_10_12	1206.07
NEK8	*BMPR1A*	v_10_13	13.41
NEK8	*NEK4*	v_10_14	7.03
NEK8	*PRKCD*	v_10_15	27.73
NEK8	*BMPR1B*	v_10_16	8.89
NEK8	*DYRK4*	v_10_17	10.81
NEK8	*LIMK2*	v_10_18	8.08
NEK8	*NME4*	v_10_19	5.77
NEK8	*LYN*	v_10_20	12.21
NEK8	*NEK9*	v_10_21	451.51
NEK8	*BMP2K*	v_10_22	29.51
NEK8	*AK7*	v_10_23	4.42
NEK8	*ULK4*	v_10_24	362.08
NEK8	*WNK1*	v_10_25	8.84
NEK8	*CDC7*	v_10_26	3.25
NEK8	*RIOK2*	v_10_27	12728.02
NEK8	*DGKB*	v_10_28	67.46
NEK8	*PTK2*	v_10_29	7.03
NEK8	*TRIB1*	v_10_30	18.23
NEK8	*PGK1*	v_10_31	5870.52
NEK8	*STK32A*	v_10_32	1104.38
NEK8	*PRKCB*	v_10_33	6.35
NEK8	*MYLK*	v_10_34	7.91
NEK8	*PI4K2B*	v_10_35	103.54
NEK8	*MAP2K1*	v_10_36	3.57
NEK8	*SGK3*	v_10_37	18.62
NEK8	*MAGI2*	v_10_38	13.42
NEK8	*CERKL*	v_10_39	8.25

NEK8	*CDK4*	v_10_40	12.61
SPHK2	*DOLK*	v_11_12	7729.28
SPHK2	*BMPR1A*	v_11_13	0.42
SPHK2	*NEK4*	v_11_14	4.64
SPHK2	*PRKCD*	v_11_15	32.00
SPHK2	*BMPR1B*	v_11_16	3.18
SPHK2	*DYRK4*	v_11_17	6.74
SPHK2	*LIMK2*	v_11_18	1.52
SPHK2	*NME4*	v_11_19	29.18
SPHK2	*LYN*	v_11_20	7.89
SPHK2	*NEK9*	v_11_21	516.33
SPHK2	*BMP2K*	v_11_22	32.23
SPHK2	*AK7*	v_11_23	39.12
SPHK2	*ULK4*	v_11_24	85.39
SPHK2	*WNK1*	v_11_25	6.76
SPHK2	*CDC7*	v_11_26	17.79
SPHK2	*RIOK2*	v_11_27	83082.08
SPHK2	*DGKB*	v_11_28	46.08
SPHK2	*PTK2*	v_11_29	4.18
SPHK2	*TRIB1*	v_11_30	27.08
SPHK2	*PGK1*	v_11_31	37871.19
SPHK2	*STK32A*	v_11_32	6518.07
SPHK2	*PRKCB*	v_11_33	174.98
SPHK2	*MYLK*	v_11_34	16.40
SPHK2	*PI4K2B*	v_11_35	92.69
SPHK2	*MAP2K1*	v_11_36	66.55
SPHK2	*SGK3*	v_11_37	5.24
SPHK2	*MAGI2*	v_11_38	12.75
SPHK2	*CERKL*	v_11_39	110.37

SPHK2	*CDK4*	v_11_40	112.63
DOLK	*BMPR1A*	v_12_13	4.31
DOLK	*NEK4*	v_12_14	1.69
DOLK	*PRKCD*	v_12_15	106.04
DOLK	*BMPR1B*	v_12_16	6.32
DOLK	*DYRK4*	v_12_17	1.61
DOLK	*LIMK2*	v_12_18	6.46
DOLK	*NME4*	v_12_19	6.95
DOLK	*LYN*	v_12_20	195.46
DOLK	*NEK9*	v_12_21	16.94
DOLK	*BMP2K*	v_12_22	80.69
DOLK	*AK7*	v_12_23	2.66
DOLK	*ULK4*	v_12_24	20.73
DOLK	*WNK1*	v_12_25	4.07
DOLK	*CDC7*	v_12_26	10.33
DOLK	*RIOK2*	v_12_27	1.02
DOLK	*DGKB*	v_12_28	10.74
DOLK	*PTK2*	v_12_29	2.20
DOLK	*TRIB1*	v_12_30	4.68
DOLK	*PGK1*	v_12_31	1.68
DOLK	*STK32A*	v_12_32	3.47
DOLK	*PRKCB*	v_12_33	21.33
DOLK	*MYLK*	v_12_34	21.05
DOLK	*PI4K2B*	v_12_35	330.62
DOLK	*MAP2K1*	v_12_36	19.67
DOLK	*SGK3*	v_12_37	3.27
DOLK	*MAGI2*	v_12_38	2.89
DOLK	*CERKL*	v_12_39	7.20
DOLK	*CDK4*	v_12_40	6.92

BMPR1A	*NEK4*	v_13_14	5.52
BMPR1A	*PRKCD*	v_13_15	7.59
BMPR1A	*BMPR1B*	v_13_16	2.67
BMPR1A	*DYRK4*	v_13_17	9.13
BMPR1A	*LIMK2*	v_13_18	2.20
BMPR1A	*NME4*	v_13_19	15.57
BMPR1A	*LYN*	v_13_20	22.57
BMPR1A	*NEK9*	v_13_21	130.32
BMPR1A	*BMP2K*	v_13_22	23.23
BMPR1A	*AK7*	v_13_23	16.34
BMPR1A	*ULK4*	v_13_24	77.52
BMPR1A	*WNK1*	v_13_25	7.87
BMPR1A	*CDC7*	v_13_26	20.10
BMPR1A	*RIOK2*	v_13_27	15994.24
BMPR1A	*DGKB*	v_13_28	15.67
BMPR1A	*PTK2*	v_13_29	3.17
BMPR1A	*TRIB1*	v_13_30	43.36
BMPR1A	*PGK1*	v_13_31	7301.21
BMPR1A	*STK32A*	v_13_32	1266.13
BMPR1A	*PRKCB*	v_13_33	58.19
BMPR1A	*MYLK*	v_13_34	31.45
BMPR1A	*PI4K2B*	v_13_35	29.50
BMPR1A	*MAP2K1*	v_13_36	29.67
BMPR1A	*SGK3*	v_13_37	4.31
BMPR1A	*MAGI2*	v_13_38	8.98
BMPR1A	*CERKL*	v_13_39	95.66
BMPR1A	*CDK4*	v_13_40	48.75
NEK4	*PRKCD*	v_14_15	17.23
NEK4	*BMPR1B*	v_14_16	0.98

NEK4	*DYRK4*	v_14_17	0.28
NEK4	*LIMK2*	v_14_18	1.06
NEK4	*NME4*	v_14_19	2.26
NEK4	*LYN*	v_14_20	19.54
NEK4	*NEK9*	v_14_21	154.41
NEK4	*BMP2K*	v_14_22	10.59
NEK4	*AK7*	v_14_23	0.87
NEK4	*ULK4*	v_14_24	16.16
NEK4	*WNK1*	v_14_25	0.31
NEK4	*CDC7*	v_14_26	1.82
NEK4	*RIOK2*	v_14_27	13508.73
NEK4	*DGKB*	v_14_28	4.27
NEK4	*PTK2*	v_14_29	0.26
NEK4	*TRIB1*	v_14_30	1.59
NEK4	*PGK1*	v_14_31	6152.36
NEK4	*STK32A*	v_14_32	1065.39
NEK4	*PRKCB*	v_14_33	4.97
NEK4	*MYLK*	v_14_34	2.30
NEK4	*PI4K2B*	v_14_35	53.62
NEK4	*MAP2K1*	v_14_36	4.37
NEK4	*SGK3*	v_14_37	0.66
NEK4	*MAGI2*	v_14_38	0.50
NEK4	*CERKL*	v_14_39	1.70
NEK4	*CDK4*	v_14_40	2.12
PRKCD	*BMPR1B*	v_15_16	3.16
PRKCD	*DYRK4*	v_15_17	10.14
PRKCD	*LIMK2*	v_15_18	0.50
PRKCD	*NME4*	v_15_19	17.89
PRKCD	*LYN*	v_15_20	14.17

PRKCD	*NEK9*	v_15_21	4193.36
PRKCD	*BMP2K*	v_15_22	1.08
PRKCD	*AK7*	v_15_23	38.77
PRKCD	*ULK4*	v_15_24	236.20
PRKCD	*WNK1*	v_15_25	5.26
PRKCD	*CDC7*	v_15_26	68.12
PRKCD	*RIOK2*	v_15_27	752870.07
PRKCD	*DGKB*	v_15_28	47.41
PRKCD	*PTK2*	v_15_29	5.78
PRKCD	*TRIB1*	v_15_30	29.68
PRKCD	*PGK1*	v_15_31	342958.78
PRKCD	*STK32A*	v_15_32	58902.60
PRKCD	*PRKCB*	v_15_33	106.22
PRKCD	*MYLK*	v_15_34	1.07
PRKCD	*PI4K2B*	v_15_35	2.90
PRKCD	*MAP2K1*	v_15_36	26.30
PRKCD	*SGK3*	v_15_37	8.11
PRKCD	*MAGI2*	v_15_38	8.97
PRKCD	*CERKL*	v_15_39	100.10
PRKCD	*CDK4*	v_15_40	91.80
BMPR1B	*DYRK4*	v_16_17	43.77
BMPR1B	*LIMK2*	v_16_18	2.57
BMPR1B	*NME4*	v_16_19	13.58
BMPR1B	*LYN*	v_16_20	21.60
BMPR1B	*NEK9*	v_16_21	133.68
BMPR1B	*BMP2K*	v_16_22	3.58
BMPR1B	*AK7*	v_16_23	16.52
BMPR1B	*ULK4*	v_16_24	109.15
BMPR1B	*WNK1*	v_16_25	22.86

BMPR1B	*CDC7*	v_16_26	5.61
BMPR1B	*RIOK2*	v_16_27	1985.40
BMPR1B	*DGKB*	v_16_28	10.94
BMPR1B	*PTK2*	v_16_29	7.82
BMPR1B	*TRIB1*	v_16_30	31.30
BMPR1B	*PGK1*	v_16_31	903.84
BMPR1B	*STK32A*	v_16_32	250.42
BMPR1B	*PRKCB*	v_16_33	18.84
BMPR1B	*MYLK*	v_16_34	2.32
BMPR1B	*PI4K2B*	v_16_35	6.41
BMPR1B	*MAP2K1*	v_16_36	36.55
BMPR1B	*SGK3*	v_16_37	79.05
BMPR1B	*MAGI2*	v_16_38	14.22
BMPR1B	*CERKL*	v_16_39	34.87
BMPR1B	*CDK4*	v_16_40	18.95
DYRK4	*LIMK2*	v_17_18	3.24
DYRK4	*NME4*	v_17_19	1.40
DYRK4	*LYN*	v_17_20	23.93
DYRK4	*NEK9*	v_17_21	200.95
DYRK4	*BMP2K*	v_17_22	33.30
DYRK4	*AK7*	v_17_23	3.13
DYRK4	*ULK4*	v_17_24	17.90
DYRK4	*WNK1*	v_17_25	0.67
DYRK4	*CDC7*	v_17_26	1.76
DYRK4	*RIOK2*	v_17_27	10419.51
DYRK4	*DGKB*	v_17_28	5.31
DYRK4	*PTK2*	v_17_29	2.07
DYRK4	*TRIB1*	v_17_30	1.52
DYRK4	*PGK1*	v_17_31	7165.56

DYRK4	*STK32A*	v_17_32	1956.92
DYRK4	*PRKCB*	v_17_33	8.29
DYRK4	*MYLK*	v_17_34	6.47
DYRK4	*PI4K2B*	v_17_35	184.88
DYRK4	*MAP2K1*	v_17_36	4.42
DYRK4	*SGK3*	v_17_37	0.80
DYRK4	*MAGI2*	v_17_38	1.90
DYRK4	*CERKL*	v_17_39	2.20
DYRK4	*CDK4*	v_17_40	2.39
LIMK2	*NME4*	v_18_19	38.20
LIMK2	*LYN*	v_18_20	23.71
LIMK2	*NEK9*	v_18_21	688.79
LIMK2	*BMP2K*	v_18_22	1.17
LIMK2	*AK7*	v_18_23	36.79
LIMK2	*ULK4*	v_18_24	91.10
LIMK2	*WNK1*	v_18_25	15.79
LIMK2	*CDC7*	v_18_26	62.27
LIMK2	*RIOK2*	v_18_27	109592.43
LIMK2	*DGKB*	v_18_28	55.54
LIMK2	*PTK2*	v_18_29	29.19
LIMK2	*TRIB1*	v_18_30	29.61
LIMK2	*PGK1*	v_18_31	49925.16
LIMK2	*STK32A*	v_18_32	8575.29
LIMK2	*PRKCB*	v_18_33	130.95
LIMK2	*MYLK*	v_18_34	51.67
LIMK2	*PI4K2B*	v_18_35	5.25
LIMK2	*MAP2K1*	v_18_36	43.54
LIMK2	*SGK3*	v_18_37	59.78
LIMK2	*MAGI2*	v_18_38	21.99

LIMK2	*CERKL*	v_18_39	92.94
LIMK2	*CDK4*	v_18_40	103.87
NME4	*LYN*	v_19_20	450.12
NME4	*NEK9*	v_19_21	1770.38
NME4	*BMP2K*	v_19_22	212.34
NME4	*AK7*	v_19_23	1459.93
NME4	*ULK4*	v_19_24	1980.44
NME4	*WNK1*	v_19_25	426.27
NME4	*CDC7*	v_19_26	1065.44
NME4	*RIOK2*	v_19_27	69676.32
NME4	*DGKB*	v_19_28	2221.19
NME4	*PTK2*	v_19_29	1790.42
NME4	*TRIB1*	v_19_30	1301.45
NME4	*PGK1*	v_19_31	31514.40
NME4	*STK32A*	v_19_32	6094.03
NME4	*PRKCB*	v_19_33	2271.75
NME4	*MYLK*	v_19_34	1049.12
NME4	*PI4K2B*	v_19_35	1741.32
NME4	*MAP2K1*	v_19_36	2776.49
NME4	*SGK3*	v_19_37	1984.95
NME4	*MAGI2*	v_19_38	1817.24
NME4	*CERKL*	v_19_39	1436.12
NME4	*CDK4*	v_19_40	1222.18
LYN	*NEK9*	v_20_21	4188.97
LYN	*BMP2K*	v_20_22	26.58
LYN	*AK7*	v_20_23	11.20
LYN	*ULK4*	v_20_24	33.22
LYN	*WNK1*	v_20_25	10.86
LYN	*CDC7*	v_20_26	8.13

LYN	*RIOK2*	v_20_27	730884.51
LYN	*DGKB*	v_20_28	6.31
LYN	*PTK2*	v_20_29	2.31
LYN	*TRIB1*	v_20_30	1.85
LYN	*PGK1*	v_20_31	334154.34
LYN	*STK32A*	v_20_32	57775.74
LYN	*PRKCB*	v_20_33	9.95
LYN	*MYLK*	v_20_34	3.67
LYN	*PI4K2B*	v_20_35	60.76
LYN	*MAP2K1*	v_20_36	6.15
LYN	*SGK3*	v_20_37	0.58
LYN	*MAGI2*	v_20_38	1.46
LYN	*CERKL*	v_20_39	12.82
LYN	*CDK4*	v_20_40	8.73
NEK9	*BMP2K*	v_21_22	297.79
NEK9	*AK7*	v_21_23	28.23
NEK9	*ULK4*	v_21_24	999.34
NEK9	*WNK1*	v_21_25	20.61
NEK9	*CDC7*	v_21_26	45.12
NEK9	*RIOK2*	v_21_27	13.06
NEK9	*DGKB*	v_21_28	182.85
NEK9	*PTK2*	v_21_29	20.22
NEK9	*TRIB1*	v_21_30	64.92
NEK9	*PGK1*	v_21_31	7.48
NEK9	*STK32A*	v_21_32	38.63
NEK9	*PRKCB*	v_21_33	78.10
NEK9	*MYLK*	v_21_34	49.08
NEK9	*PI4K2B*	v_21_35	1703.13
NEK9	*MAP2K1*	v_21_36	331.36

NEK9	*SGK3*	v_21_37	81.03
NEK9	*MAGI2*	v_21_38	68.79
NEK9	*CERKL*	v_21_39	24.46
NEK9	*CDK4*	v_21_40	30.61
BMP2K	*AK7*	v_22_23	250.51
BMP2K	*ULK4*	v_22_24	422.56
BMP2K	*WNK1*	v_22_25	103.39
BMP2K	*CDC7*	v_22_26	94.98
BMP2K	*RIOK2*	v_22_27	122283.86
BMP2K	*DGKB*	v_22_28	282.49
BMP2K	*PTK2*	v_22_29	118.02
BMP2K	*TRIB1*	v_22_30	307.71
BMP2K	*PGK1*	v_22_31	55739.32
BMP2K	*STK32A*	v_22_32	9636.57
BMP2K	*PRKCB*	v_22_33	1069.23
BMP2K	*MYLK*	v_22_34	16.86
BMP2K	*PI4K2B*	v_22_35	4.68
BMP2K	*MAP2K1*	v_22_36	410.26
BMP2K	*SGK3*	v_22_37	155.57
BMP2K	*MAGI2*	v_22_38	442.53
BMP2K	*CERKL*	v_22_39	547.69
BMP2K	*CDK4*	v_22_40	665.66
AK7	*ULK4*	v_23_24	7.10
AK7	*WNK1*	v_23_25	1.41
AK7	*CDC7*	v_23_26	1.22
AK7	*RIOK2*	v_23_27	1042.23
AK7	*DGKB*	v_23_28	3.57
AK7	*PTK2*	v_23_29	1.17
AK7	*TRIB1*	v_23_30	3.90

AK7	*PGK1*	v_23_31	517.88
AK7	*STK32A*	v_23_32	148.57
AK7	*PRKCB*	v_23_33	6.41
AK7	*MYLK*	v_23_34	9.10
AK7	*PI4K2B*	v_23_35	265.30
AK7	*MAP2K1*	v_23_36	1.76
AK7	*SGK3*	v_23_37	2.67
AK7	*MAGI2*	v_23_38	2.32
AK7	*CERKL*	v_23_39	0.63
AK7	*CDK4*	v_23_40	0.70
ULK4	*WNK1*	v_24_25	1.78
ULK4	*CDC7*	v_24_26	0.53
ULK4	*RIOK2*	v_24_27	1494.65
ULK4	*DGKB*	v_24_28	1.77
ULK4	*PTK2*	v_24_29	1.78
ULK4	*TRIB1*	v_24_30	2.45
ULK4	*PGK1*	v_24_31	717.04
ULK4	*STK32A*	v_24_32	162.77
ULK4	*PRKCB*	v_24_33	2.05
ULK4	*MYLK*	v_24_34	7.49
ULK4	*PI4K2B*	v_24_35	225.89
ULK4	*MAP2K1*	v_24_36	2.33
ULK4	*SGK3*	v_24_37	4.48
ULK4	*MAGI2*	v_24_38	4.94
ULK4	*CERKL*	v_24_39	0.25
ULK4	*CDK4*	v_24_40	0.64
WNK1	*CDC7*	v_25_26	1.71
WNK1	*RIOK2*	v_25_27	103637.58
WNK1	*DGKB*	v_25_28	8.93

WNK1	*PTK2*	v_25_29	0.38
WNK1	*TRIB1*	v_25_30	1.54
WNK1	*PGK1*	v_25_31	47229.74
WNK1	*STK32A*	v_25_32	8117.91
WNK1	*PRKCB*	v_25_33	3.65
WNK1	*MYLK*	v_25_34	6.27
WNK1	*PI4K2B*	v_25_35	200.56
WNK1	*MAP2K1*	v_25_36	4.04
WNK1	*SGK3*	v_25_37	0.52
WNK1	*MAGI2*	v_25_38	0.51
WNK1	*CERKL*	v_25_39	3.70
WNK1	*CDK4*	v_25_40	9.63
CDC7	*RIOK2*	v_26_27	1533.12
CDC7	*DGKB*	v_26_28	44.48
CDC7	*PTK2*	v_26_29	31.05
CDC7	*TRIB1*	v_26_30	28.15
CDC7	*PGK1*	v_26_31	1153.93
CDC7	*STK32A*	v_26_32	1813.34
CDC7	*PRKCB*	v_26_33	9.13
CDC7	*MYLK*	v_26_34	59.75
CDC7	*PI4K2B*	v_26_35	636.16
CDC7	*MAP2K1*	v_26_36	18.55
CDC7	*SGK3*	v_26_37	33.74
CDC7	*MAGI2*	v_26_38	45.02
CDC7	*CERKL*	v_26_39	3.62
CDC7	*CDK4*	v_26_40	1.96
RIOK2	*DGKB*	v_27_28	123.99
RIOK2	*PTK2*	v_27_29	146.60
RIOK2	*TRIB1*	v_27_30	99.34

RIOK2	*PGK1*	v_27_31	79.06
RIOK2	*STK32A*	v_27_32	38.52
RIOK2	*PRKCB*	v_27_33	269.39
RIOK2	*MYLK*	v_27_34	153.86
RIOK2	*PI4K2B*	v_27_35	597.89
RIOK2	*MAP2K1*	v_27_36	663.11
RIOK2	*SGK3*	v_27_37	669.34
RIOK2	*MAGI2*	v_27_38	1535.23
RIOK2	*CERKL*	v_27_39	49.78
RIOK2	*CDK4*	v_27_40	263.02
DGKB	*PTK2*	v_28_29	4.00
DGKB	*TRIB1*	v_28_30	9.13
DGKB	*PGK1*	v_28_31	11596.87
DGKB	*STK32A*	v_28_32	2087.02
DGKB	*PRKCB*	v_28_33	10.49
DGKB	*MYLK*	v_28_34	6.51
DGKB	*PI4K2B*	v_28_35	62.91
DGKB	*MAP2K1*	v_28_36	1.09
DGKB	*SGK3*	v_28_37	8.07
DGKB	*MAGI2*	v_28_38	9.22
DGKB	*CERKL*	v_28_39	11.43
DGKB	*CDK4*	v_28_40	10.26
PTK2	*TRIB1*	v_29_30	1.41
PTK2	*PGK1*	v_29_31	7891.05
PTK2	*STK32A*	v_29_32	1366.96
PTK2	*PRKCB*	v_29_33	2.17
PTK2	*MYLK*	v_29_34	8.21
PTK2	*PI4K2B*	v_29_35	80.74
PTK2	*MAP2K1*	v_29_36	6.34

PTK2	*SGK3*	v_29_37	0.53
PTK2	*MAGI2*	v_29_38	0.62
PTK2	*CERKL*	v_29_39	2.44
PTK2	*CDK4*	v_29_40	2.32
TRIB1	*PGK1*	v_30_31	218.25
TRIB1	*STK32A*	v_30_32	1566.90
TRIB1	*PRKCB*	v_30_33	11.30
TRIB1	*MYLK*	v_30_34	111.71
TRIB1	*PI4K2B*	v_30_35	421.94
TRIB1	*MAP2K1*	v_30_36	100.72
TRIB1	*SGK3*	v_30_37	0.88
TRIB1	*MAGI2*	v_30_38	2.66
TRIB1	*CERKL*	v_30_39	12.37
TRIB1	*CDK4*	v_30_40	38.66
PGK1	*STK32A*	v_31_32	3.05
PGK1	*PRKCB*	v_31_33	19.13
PGK1	*MYLK*	v_31_34	10.83
PGK1	*PI4K2B*	v_31_35	184.94
PGK1	*MAP2K1*	v_31_36	7.27
PGK1	*SGK3*	v_31_37	7.18
PGK1	*MAGI2*	v_31_38	7.75
PGK1	*CERKL*	v_31_39	7.33
PGK1	*CDK4*	v_31_40	11.03
STK32A	*PRKCB*	v_32_33	9.05
STK32A	*MYLK*	v_32_34	37.83
STK32A	*PI4K2B*	v_32_35	317.48
STK32A	*MAP2K1*	v_32_36	33.75
STK32A	*SGK3*	v_32_37	11.03
STK32A	*MAGI2*	v_32_38	7.61

STK32A	*CERKL*	v_32_39	6.86
STK32A	*CDK4*	v_32_40	8.95
PRKCB	*MYLK*	v_33_34	4.45
PRKCB	*PI4K2B*	v_33_35	100.99
PRKCB	*MAP2K1*	v_33_36	1.30
PRKCB	*SGK3*	v_33_37	1.77
PRKCB	*MAGI2*	v_33_38	2.37
PRKCB	*CERKL*	v_33_39	2.12
PRKCB	*CDK4*	v_33_40	1.25
MYLK	*PI4K2B*	v_34_35	3.31
MYLK	*MAP2K1*	v_34_36	94.91
MYLK	*SGK3*	v_34_37	55.59
MYLK	*MAGI2*	v_34_38	93.99
MYLK	*CERKL*	v_34_39	141.81
MYLK	*CDK4*	v_34_40	166.24
PI4K2B	*MAP2K1*	v_35_36	241268.08
PI4K2B	*SGK3*	v_35_37	10551.15
PI4K2B	*MAGI2*	v_35_38	35288.93
PI4K2B	*CERKL*	v_35_39	313089.97
PI4K2B	*CDK4*	v_35_40	375524.59
MAP2K1	*SGK3*	v_36_37	8.63
MAP2K1	*MAGI2*	v_36_38	17.72
MAP2K1	*CERKL*	v_36_39	13.52
MAP2K1	*CDK4*	v_36_40	3.65
SGK3	*MAGI2*	v_37_38	2.20
SGK3	*CERKL*	v_37_39	70.28
SGK3	*CDK4*	v_37_40	91.63
MAGI2	*CERKL*	v_38_39	314.97
MAGI2	*CDK4*	v_38_40	433.02

CERKL	*CDK4*	v_39_40	0.36

Table S6. Association analysis of gene ratios with recurrence

Gene N (numerator)	Gene D (denominator)	Variables	No recurrence mean(SD)	Recurrence mean(SD)	HR (95%CI)	P value
			**Training set**			
MAP4K1	*STK39*	v_1_3	2.22(2.26)	2.77(3.37)	1.07(0.91-1.26)	0.44
MAP4K1	*DOLK*	v_1_12	1.09(0.72)	1.85(2.66)	1.17(0.96-1.42)	0.12
MAP4K1	*NEK4*	v_1_14	1.94(1.83)	2.75(3.44)	1.19(0.99-1.44)	0.06
MAP4K1	*RIOK2*	v_1_27	1.56(1.47)	1.64(1.62)	1.00(0.73-1.38)	0.98
MAP4K1	*PTK2*	v_1_29	2.19(2.83)	2.53(2.82)	1.02(0.89-1.18)	0.74
CDKL4	*STK39*	v_2_3	1.18(0.42)	1.21(0.65)	1.49(0.50-4.45)	0.48
CDKL4	*PRKD3*	v_2_4	1.92(1.51)	2.31(1.67)	1.35(0.98-1.85)	0.07
CDKL4	*AGK*	v_2_5	1.21(0.76)	1.41(0.70)	1.59(0.81-3.09)	0.18
CDKL4	*PIK3CA*	v_2_6	3.69(4.35)	1.48(1.28)	0.85(0.70-1.04)	0.11
CDKL4	*PKN3*	v_2_7	2.68(3.93)	2.68(3.51)	1.05(0.94-1.17)	0.38
CDKL4	*MST1R*	v_2_9	1.45(2.35)	1.62(3.33)	0.99(0.85-1.16)	0.93
CDKL4	*NEK8*	v_2_10	2.53(4.52)	1.70(2.29)	0.98(0.86-1.12)	0.78
CDKL4	*SPHK2*	v_2_11	1.66(1.45)	2.35(4.01)	1.17(1.02-1.35)	0.02
CDKL4	*BMPR1A*	v_2_13	1.52(1.42)	2.48(5.14)	1.12(1.01-1.25)	0.04
CDKL4	*NEK4*	v_2_14	1.06(0.32)	1.78(3.16)	1.24(1.05-1.45)	0.01
CDKL4	*BMPR1B*	v_2_16	1.51(1.79)	2.36(5.54)	1.11(1.00-1.22)	0.06
CDKL4	*DYRK4*	v_2_17	1.08(0.44)	1.26(1.01)	1.46(0.76-2.81)	0.26
CDKL4	*LIMK2*	v_2_18	1.61(1.87)	2.73(6.60)	1.09(1.00-1.19)	0.04
CDKL4	*AK7*	v_2_23	1.26(1.21)	2.03(4.59)	1.14(1.01-1.29)	0.04
CDKL4	*WNK1*	v_2_25	1.10(0.61)	1.31(0.55)	8.03(2.87-22.47)	<0.001
CDKL4	*CDC7*	v_2_26	1.36(1.44)	1.86(3.32)	1.20(1.00-1.43)	0.05
CDKL4	*PTK2*	v_2_29	1.08(0.53)	1.59(2.25)	1.34(1.07-1.69)	0.01
CDKL4	*TRIB1*	v_2_30	1.33(0.93)	1.39(1.09)	1.00(0.63-1.58)	0.99
CDKL4	*MYLK*	v_2_34	2.05(2.61)	2.81(5.68)	1.09(0.99-1.20)	0.08

CDKL4	*MAP2K1*	v_2_36	1.87(2.79)	2.50(5.29)	1.07(0.96-1.18)	0.21
CDKL4	*SGK3*	v_2_37	1.41(0.95)	1.68(1.07)	1.57(0.93-2.62)	0.09
CDKL4	*MAGI2*	v_2_38	1.34(0.86)	1.79(1.22)	1.96(1.29-2.97)	0.002
CDKL4	*CERKL*	v_2_39	1.43(1.43)	1.72(3.19)	1.15(0.93-1.43)	0.20
STK39	*PRKD3*	v_3_4	1.64(1.09)	2.00(1.20)	1.43(0.96-2.14)	0.08
STK39	*AGK*	v_3_5	1.12(0.69)	1.31(0.83)	1.92(0.89-4.13)	0.10
STK39	*PKN3*	v_3_7	2.29(3.28)	2.50(3.23)	1.05(0.93-1.19)	0.43
STK39	*MST1R*	v_3_9	1.43(2.12)	1.16(1.34)	0.91(0.69-1.21)	0.53
STK39	*NEK8*	v_3_10	2.35(3.53)	1.66(2.23)	0.98(0.84-1.16)	0.85
STK39	*SPHK2*	v_3_11	1.77(2.72)	1.71(1.54)	1.03(0.86-1.22)	0.77
STK39	*BMPR1A*	v_3_13	1.81(3.51)	1.78(1.79)	1.01(0.88-1.17)	0.88
STK39	*NEK4*	v_3_14	1.04(0.61)	1.35(1.21)	1.48(0.96-2.29)	0.08
STK39	*BMPR1B*	v_3_16	1.58(2.39)	1.64(1.89)	1.06(0.88-1.28)	0.55
STK39	*DYRK4*	v_3_17	0.99(0.39)	1.04(0.46)	1.06(0.30-3.73)	0.92
STK39	*LIMK2*	v_3_18	1.76(2.67)	1.75(2.18)	1.04(0.87-1.25)	0.63
STK39	*AK7*	v_3_23	1.30(1.75)	1.41(1.76)	1.11(0.86-1.42)	0.42
STK39	*WNK1*	v_3_25	1.07(0.74)	1.28(0.74)	2.59(1.38-4.87)	0.003
STK39	*CDC7*	v_3_26	1.58(2.48)	1.54(1.96)	1.05(0.86-1.28)	0.61
STK39	*PTK2*	v_3_29	1.03(0.66)	1.26(0.95)	1.65(0.92-2.96)	0.09
STK39	*TRIB1*	v_3_30	1.31(1.07)	1.28(1.11)	0.94(0.59-1.49)	0.78
STK39	*MYLK*	v_3_34	2.26(3.75)	2.12(2.57)	1.03(0.92-1.16)	0.63
STK39	*MAP2K1*	v_3_36	2.08(4.02)	1.87(2.19)	1.01(0.89-1.15)	0.85
STK39	*SGK3*	v_3_37	1.23(0.70)	1.45(0.87)	1.61(0.87-3.01)	0.13
STK39	*MAGI2*	v_3_38	1.24(1.03)	1.53(0.74)	1.53(1.05-2.22)	0.03
STK39	*CERKL*	v_3_39	1.59(1.99)	1.38(1.78)	1.02(0.77-1.35)	0.88
STK39	*CDK4*	v_3_40	1.64(2.69)	1.85(3.58)	1.05(0.90-1.23)	0.51
PRKD3	*AGK*	v_4_5	1.90(3.34)	1.53(2.09)	0.90(0.75-1.09)	0.28
PRKD3	*WNK1*	v_4_25	1.97(3.63)	1.45(1.81)	0.91(0.72-1.15)	0.43
PRKD3	*SGK3*	v_4_37	1.55(2.29)	1.26(1.36)	0.84(0.64-1.11)	0.22

AGK	*DYRK4*	v_5_17	1.54(2.27)	0.96(0.62)	0.58(0.24-1.45)	0.25
AGK	*LIMK2*	v_5_18	1.84(2.61)	2.09(4.03)	1.05(0.90-1.22)	0.54
AGK	*PTK2*	v_5_29	1.42(2.01)	1.54(1.99)	0.99(0.80-1.23)	0.93
AGK	*MYLK*	v_5_34	2.26(3.13)	2.19(3.49)	1.02(0.89-1.18)	0.78
AGK	*SGK3*	v_5_37	1.21(0.65)	1.35(0.76)	1.58(0.76-3.27)	0.22
AGK	*MAGI2*	v_5_38	1.48(1.25)	1.60(1.35)	1.12(0.81-1.55)	0.49
HK2	*SPHK2*	v_8_11	1.51(1.18)	1.75(1.24)	1.28(0.86-1.92)	0.23
HK2	*BMPR1A*	v_8_13	1.26(0.89)	1.51(0.64)	1.16(0.70-1.90)	0.57
HK2	*NEK4*	v_8_14	1.78(2.39)	2.60(4.34)	1.10(0.97-1.25)	0.15
HK2	*PRKCD*	v_8_15	1.11(0.65)	1.40(0.67)	1.75(0.84-3.63)	0.13
HK2	*BMPR1B*	v_8_16	1.47(1.20)	1.43(0.94)	1.05(0.70-1.59)	0.80
HK2	*DYRK4*	v_8_17	1.84(2.33)	2.00(2.76)	1.05(0.86-1.27)	0.65
HK2	*LIMK2*	v_8_18	1.17(0.70)	1.34(0.62)	1.39(0.72-2.70)	0.33
HK2	*BMP2K*	v_8_22	1.29(0.87)	1.41(0.71)	1.13(0.71-1.79)	0.60
HK2	*WNK1*	v_8_25	1.80(2.51)	2.35(3.19)	1.11(0.95-1.28)	0.18
HK2	*CDC7*	v_8_26	1.88(2.91)	2.47(6.39)	1.05(0.95-1.15)	0.36
HK2	*PTK2*	v_8_29	1.69(2.16)	2.28(3.22)	1.12(0.95-1.32)	0.18
HK2	*TRIB1*	v_8_30	1.90(2.75)	2.97(4.87)	1.08(0.97-1.21)	0.18
HK2	*MYLK*	v_8_34	1.50(1.47)	1.38(1.10)	1.04(0.74-1.45)	0.82
HK2	*PI4K2B*	v_8_35	1.74(1.45)	2.46(2.33)	1.15(0.91-1.45)	0.25
HK2	*SGK3*	v_8_37	2.20(2.90)	3.31(4.76)	1.11(0.99-1.24)	0.08
HK2	*MAGI2*	v_8_38	2.36(3.68)	3.26(5.52)	1.07(0.98-1.18)	0.14
MST1R	*SPHK2*	v_9_11	1.97(1.84)	2.30(1.71)	1.21(0.95-1.54)	0.11
MST1R	*BMPR1A*	v_9_13	1.71(1.65)	2.39(2.12)	1.23(0.97-1.56)	0.09
MST1R	*NEK4*	v_9_14	1.48(1.01)	1.83(1.74)	1.30(0.97-1.74)	0.08
MST1R	*BMPR1B*	v_9_16	1.98(3.18)	1.98(1.66)	1.02(0.89-1.17)	0.72
MST1R	*DYRK4*	v_9_17	1.52(1.23)	1.80(1.45)	1.18(0.84-1.65)	0.33
MST1R	*LIMK2*	v_9_18	1.95(3.04)	2.13(1.57)	1.04(0.91-1.18)	0.56
MST1R	*NME4*	v_9_19	1.90(1.90)	2.07(2.82)	1.19(0.96-1.47)	0.12

MST1R	*AK7*	v_9_23	1.14(0.55)	1.18(0.45)	1.70(0.73-3.97)	0.22
MST1R	*ULK4*	v_9_24	1.24(0.79)	1.42(1.16)	1.26(0.77-2.05)	0.36
MST1R	*WNK1*	v_9_25	1.48(1.00)	2.09(1.55)	1.87(1.30-2.71)	0.001
MST1R	*CDC7*	v_9_26	1.47(1.02)	1.47(1.45)	1.18(0.76-1.83)	0.47
MST1R	*DGKB*	v_9_28	1.75(2.37)	1.26(0.89)	0.94(0.60-1.47)	0.78
MST1R	*PTK2*	v_9_29	1.47(1.08)	1.88(1.62)	1.34(0.95-1.87)	0.09
MST1R	*TRIB1*	v_9_30	1.61(1.29)	1.91(1.59)	1.18(0.87-1.62)	0.29
MST1R	*MYLK*	v_9_34	2.58(4.51)	2.84(3.08)	1.03(0.95-1.12)	0.44
MST1R	*MAP2K1*	v_9_36	1.72(1.45)	2.13(2.15)	1.37(1.04-1.81)	0.02
MST1R	*SGK3*	v_9_37	1.99(1.67)	2.85(2.79)	1.37(1.09-1.72)	0.01
MST1R	*MAGI2*	v_9_38	2.01(1.88)	2.80(2.66)	1.24(1.03-1.49)	0.03
MST1R	*CERKL*	v_9_39	1.35(1.13)	1.14(0.56)	0.94(0.56-1.57)	0.82
MST1R	*CDK4*	v_9_40	1.48(1.30)	1.29(0.91)	0.97(0.66-1.41)	0.86
NEK8	*NEK4*	v_10_14	2.42(3.46)	2.36(2.92)	0.94(0.79-1.11)	0.44
NEK8	*BMPR1B*	v_10_16	2.33(4.05)	2.47(3.67)	0.98(0.86-1.13)	0.80
NEK8	*LIMK2*	v_10_18	2.27(3.87)	2.35(3.29)	0.96(0.82-1.12)	0.60
NEK8	*NME4*	v_10_19	1.68(3.16)	1.40(0.98)	0.92(0.75-1.13)	0.44
NEK8	*AK7*	v_10_23	2.02(2.98)	1.41(1.31)	0.83(0.65-1.06)	0.14
NEK8	*WNK1*	v_10_25	2.10(3.10)	2.86(4.70)	1.03(0.91-1.17)	0.61
NEK8	*CDC7*	v_10_26	1.57(1.94)	1.25(0.98)	0.80(0.57-1.13)	0.21
NEK8	*PTK2*	v_10_29	2.20(3.38)	2.63(3.71)	0.97(0.84-1.13)	0.73
NEK8	*PRKCB*	v_10_33	2.26(3.70)	1.52(1.51)	0.85(0.67-1.07)	0.17
NEK8	*MYLK*	v_10_34	2.29(2.93)	3.14(5.41)	1.04(0.92-1.18)	0.51
NEK8	*MAP2K1*	v_10_36	1.88(2.77)	1.56(1.05)	0.85(0.64-1.14)	0.28
NEK8	*CERKL*	v_10_39	2.61(3.95)	1.54(2.03)	0.83(0.67-1.03)	0.09
SPHK2	*BMPR1A*	v_11_13	1.16(0.73)	1.15(0.50)	0.73(0.34-1.57)	0.42
SPHK2	*NEK4*	v_11_14	2.05(3.13)	1.62(2.12)	0.95(0.77-1.18)	0.67
SPHK2	*BMPR1B*	v_11_16	1.85(2.74)	1.11(0.68)	0.84(0.59-1.19)	0.32
SPHK2	*DYRK4*	v_11_17	2.49(3.90)	1.50(1.88)	0.88(0.70-1.11)	0.29

SPHK2	*LIMK2*	v_11_18	1.47(1.83)	1.08(0.65)	0.78(0.45-1.35)	0.37
SPHK2	*LYN*	v_11_20	2.23(4.22)	1.25(1.15)	0.91(0.69-1.20)	0.51
SPHK2	*WNK1*	v_11_25	2.30(3.91)	1.63(1.69)	0.95(0.79-1.15)	0.60
SPHK2	*PTK2*	v_11_29	1.95(3.01)	1.50(1.76)	0.92(0.72-1.19)	0.53
SPHK2	*SGK3*	v_11_37	2.14(3.30)	2.01(2.39)	1.00(0.84-1.18)	0.99
DOLK	*BMPR1A*	v_12_13	2.08(2.04)	2.97(4.01)	1.23(1.01-1.49)	0.03
DOLK	*NEK4*	v_12_14	1.90(1.39)	2.02(1.82)	1.18(0.83-1.68)	0.37
DOLK	*BMPR1B*	v_12_16	2.38(3.47)	2.38(3.37)	1.04(0.92-1.17)	0.51
DOLK	*DYRK4*	v_12_17	1.80(1.25)	2.23(2.38)	1.24(0.90-1.70)	0.18
DOLK	*LIMK2*	v_12_18	2.45(3.26)	2.90(4.01)	1.06(0.95-1.18)	0.32
DOLK	*NME4*	v_12_19	2.64(2.72)	3.72(5.29)	1.18(1.03-1.35)	0.02
DOLK	*AK7*	v_12_23	1.71(1.68)	2.07(3.18)	1.08(0.91-1.29)	0.38
DOLK	*WNK1*	v_12_25	1.95(1.53)	2.75(4.01)	1.32(1.11-1.58)	0.002
DOLK	*RIOK2*	v_12_27	1.57(1.39)	1.06(0.80)	0.66(0.37-1.18)	0.16
DOLK	*PTK2*	v_12_29	2.03(2.09)	1.81(1.50)	0.98(0.73-1.31)	0.88
DOLK	*TRIB1*	v_12_30	2.30(2.57)	2.74(3.47)	1.03(0.90-1.18)	0.68
DOLK	*PGK1*	v_12_31	1.54(1.81)	1.65(1.31)	1.10(0.87-1.39)	0.42
DOLK	*STK32A*	v_12_32	1.37(1.15)	1.34(0.96)	0.98(0.59-1.62)	0.92
DOLK	*SGK3*	v_12_37	2.40(2.06)	3.02(3.28)	1.12(0.93-1.36)	0.23
DOLK	*MAGI2*	v_12_38	2.44(2.06)	2.95(2.83)	1.18(0.97-1.42)	0.10
DOLK	*CERKL*	v_12_39	2.12(2.46)	2.69(5.63)	1.06(0.95-1.18)	0.33
DOLK	*CDK4*	v_12_40	2.18(2.91)	2.40(4.95)	1.02(0.90-1.15)	0.80
BMPR1A	*NEK4*	v_13_14	2.26(3.44)	1.59(2.27)	0.96(0.79-1.17)	0.69
BMPR1A	*PRKCD*	v_13_15	1.86(4.30)	1.07(0.68)	0.89(0.59-1.33)	0.56
BMPR1A	*BMPR1B*	v_13_16	1.89(2.51)	0.98(0.42)	0.76(0.48-1.22)	0.26
BMPR1A	*DYRK4*	v_13_17	2.72(4.59)	1.37(1.69)	0.89(0.71-1.12)	0.33
BMPR1A	*LIMK2*	v_13_18	1.61(2.17)	0.97(0.39)	0.74(0.38-1.43)	0.36
BMPR1A	*WNK1*	v_13_25	2.51(4.27)	1.54(1.67)	0.95(0.78-1.14)	0.55
BMPR1A	*PTK2*	v_13_29	1.97(2.53)	1.44(1.81)	0.94(0.73-1.22)	0.63

BMPR1A	*SGK3*	v_13_37	2.27(2.88)	2.04(2.47)	1.02(0.85-1.23)	0.84
BMPR1A	*MAGI2*	v_13_38	2.79(4.36)	2.00(2.95)	0.99(0.85-1.14)	0.86
NEK4	*BMPR1B*	v_14_16	1.39(1.41)	1.27(0.85)	1.02(0.71-1.46)	0.92
NEK4	*DYRK4*	v_14_17	1.09(0.53)	1.09(0.57)	0.79(0.31-2.02)	0.62
NEK4	*LIMK2*	v_14_18	1.46(1.40)	1.39(0.89)	1.04(0.72-1.50)	0.82
NEK4	*NME4*	v_14_19	1.61(1.71)	1.36(1.53)	1.00(0.75-1.35)	0.98
NEK4	*AK7*	v_14_23	1.25(1.25)	1.05(1.10)	0.96(0.61-1.51)	0.86
NEK4	*WNK1*	v_14_25	1.10(0.63)	1.27(0.66)	2.52(1.16-5.48)	0.02
NEK4	*CDC7*	v_14_26	1.46(1.75)	1.34(1.68)	1.05(0.77-1.43)	0.77
NEK4	*DGKB*	v_14_28	1.96(2.98)	1.01(0.91)	0.81(0.54-1.20)	0.29
NEK4	*PTK2*	v_14_29	1.03(0.45)	1.05(0.43)	1.04(0.33-3.24)	0.95
NEK4	*TRIB1*	v_14_30	1.40(1.14)	1.22(0.97)	0.80(0.49-1.32)	0.38
NEK4	*PRKCB*	v_14_33	1.89(3.33)	1.19(1.66)	0.92(0.68-1.25)	0.60
NEK4	*MYLK*	v_14_34	1.90(2.09)	1.89(1.79)	1.09(0.89-1.32)	0.41
NEK4	*MAP2K1*	v_14_36	1.92(3.13)	1.54(1.36)	0.99(0.83-1.18)	0.93
NEK4	*SGK3*	v_14_37	1.38(0.91)	1.61(1.12)	1.40(0.84-2.32)	0.20
NEK4	*MAGI2*	v_14_38	1.30(0.79)	1.49(0.78)	1.61(0.92-2.82)	0.09
NEK4	*CERKL*	v_14_39	1.52(1.74)	1.14(1.69)	0.90(0.62-1.32)	0.60
NEK4	*CDK4*	v_14_40	1.48(1.89)	1.35(1.99)	1.01(0.75-1.35)	0.97
PRKCD	*BMPR1B*	v_15_16	1.77(2.72)	1.25(1.06)	0.91(0.68-1.22)	0.53
PRKCD	*LIMK2*	v_15_18	1.26(0.93)	1.00(0.29)	0.61(0.24-1.53)	0.29
PRKCD	*BMP2K*	v_15_22	1.50(1.35)	1.38(1.29)	1.05(0.70-1.57)	0.81
PRKCD	*WNK1*	v_15_25	2.07(2.55)	2.48(4.46)	1.06(0.93-1.21)	0.37
PRKCD	*PTK2*	v_15_29	1.89(2.32)	2.53(5.23)	1.06(0.94-1.19)	0.35
PRKCD	*MYLK*	v_15_34	1.65(1.45)	1.18(0.90)	0.87(0.58-1.33)	0.53
PRKCD	*PI4K2B*	v_15_35	1.88(2.35)	2.07(2.03)	0.99(0.84-1.18)	0.95
PRKCD	*SGK3*	v_15_37	2.35(2.97)	3.22(5.85)	1.06(0.96-1.17)	0.27
PRKCD	*MAGI2*	v_15_38	2.59(3.88)	2.72(4.70)	1.04(0.93-1.16)	0.49
BMPR1B	*LIMK2*	v_16_18	1.61(2.09)	1.59(2.31)	0.94(0.76-1.17)	0.58

BMPR1B	*BMP2K*	v_16_22	1.89(2.84)	1.43(1.29)	0.95(0.74-1.23)	0.71
BMPR1B	*CDC7*	v_16_26	1.81(2.63)	2.15(4.47)	1.02(0.89-1.16)	0.79
BMPR1B	*PTK2*	v_16_29	1.98(4.15)	1.85(2.51)	0.96(0.84-1.10)	0.58
BMPR1B	*MYLK*	v_16_34	1.85(2.20)	1.45(1.36)	0.92(0.71-1.19)	0.52
BMPR1B	*PI4K2B*	v_16_35	2.82(3.66)	2.63(2.61)	0.95(0.82-1.11)	0.55
DYRK4	*LIMK2*	v_17_18	1.82(2.61)	1.84(1.83)	1.04(0.88-1.23)	0.67
DYRK4	*NME4*	v_17_19	1.62(1.46)	1.57(1.56)	1.07(0.80-1.42)	0.65
DYRK4	*AK7*	v_17_23	1.31(1.28)	2.01(4.22)	1.09(0.95-1.24)	0.21
DYRK4	*WNK1*	v_17_25	1.19(0.91)	1.52(1.33)	1.44(1.02-2.02)	0.04
DYRK4	*CDC7*	v_17_26	1.52(1.82)	1.37(1.30)	1.04(0.78-1.39)	0.81
DYRK4	*DGKB*	v_17_28	2.12(3.34)	1.20(1.26)	0.89(0.68-1.15)	0.36
DYRK4	*PTK2*	v_17_29	1.10(0.62)	1.90(3.62)	1.12(0.97-1.29)	0.13
DYRK4	*TRIB1*	v_17_30	1.32(0.92)	1.52(1.86)	1.09(0.79-1.50)	0.59
DYRK4	*PRKCB*	v_17_33	2.04(4.31)	1.58(2.12)	1.01(0.85-1.20)	0.89
DYRK4	*MYLK*	v_17_34	2.35(3.88)	1.78(1.46)	1.00(0.87-1.15)	0.98
DYRK4	*MAP2K1*	v_17_36	1.95(2.95)	1.85(2.01)	1.02(0.88-1.17)	0.83
DYRK4	*SGK3*	v_17_37	1.36(0.94)	1.72(1.01)	1.65(1.03-2.66)	0.04
DYRK4	*MAGI2*	v_17_38	1.37(1.08)	2.18(2.94)	1.17(1.01-1.37)	0.04
DYRK4	*CERKL*	v_17_39	1.55(1.63)	1.64(2.77)	1.06(0.85-1.33)	0.60
DYRK4	*CDK4*	v_17_40	1.49(1.95)	1.61(2.26)	1.07(0.86-1.33)	0.55
LIMK2	*BMP2K*	v_18_22	1.48(1.40)	1.42(1.15)	1.04(0.74-1.45)	0.83
LIMK2	*PI4K2B*	v_18_35	2.14(3.23)	2.23(2.26)	1.00(0.86-1.17)	0.95
LYN	*CDC7*	v_20_26	2.31(4.37)	1.36(1.54)	0.92(0.75-1.12)	0.38
LYN	*DGKB*	v_20_28	2.17(3.72)	1.12(1.14)	0.82(0.56-1.19)	0.29
LYN	*PTK2*	v_20_29	1.40(1.62)	1.66(2.94)	1.00(0.79-1.25)	0.98
LYN	*TRIB1*	v_20_30	1.57(1.77)	1.38(1.53)	0.86(0.61-1.23)	0.41
LYN	*PRKCB*	v_20_33	2.58(4.75)	1.51(1.96)	0.90(0.73-1.11)	0.33
LYN	*MYLK*	v_20_34	2.25(2.77)	1.64(1.43)	0.94(0.75-1.17)	0.57
LYN	*MAP2K1*	v_20_36	2.30(3.63)	1.61(1.63)	0.93(0.77-1.13)	0.46

LYN	*SGK3*	v_20_37	1.29(0.74)	1.52(0.85)	1.37(0.74-2.55)	0.32
LYN	*MAGI2*	v_20_38	1.41(1.06)	1.92(2.37)	1.17(0.94-1.46)	0.16
LYN	*CDK4*	v_20_40	2.19(4.39)	1.64(2.37)	0.95(0.79-1.14)	0.58
NEK9	*PGK1*	v_21_31	2.62(3.97)	2.19(2.45)	0.95(0.82-1.10)	0.49
BMP2K	*PI4K2B*	v_22_35	2.13(3.08)	1.95(2.15)	0.92(0.76-1.11)	0.39
AK7	*ULK4*	v_23_24	1.77(4.13)	1.27(0.85)	0.91(0.68-1.20)	0.49
AK7	*WNK1*	v_23_25	1.54(1.19)	2.31(2.29)	1.17(0.95-1.43)	0.14
AK7	*CDC7*	v_23_26	1.48(1.43)	1.37(1.30)	0.97(0.68-1.38)	0.87
AK7	*DGKB*	v_23_28	1.94(2.66)	1.30(1.08)	0.82(0.59-1.15)	0.25
AK7	*PTK2*	v_23_29	1.47(1.14)	1.94(1.95)	1.09(0.84-1.41)	0.52
AK7	*TRIB1*	v_23_30	1.87(2.08)	1.97(1.76)	0.97(0.78-1.21)	0.79
AK7	*PRKCB*	v_23_33	1.85(3.92)	1.18(0.73)	0.84(0.50-1.38)	0.49
AK7	*MYLK*	v_23_34	2.58(4.19)	3.23(3.83)	1.03(0.95-1.12)	0.49
AK7	*MAP2K1*	v_23_36	1.64(1.35)	2.26(2.47)	1.22(0.96-1.55)	0.10
AK7	*SGK3*	v_23_37	1.97(1.94)	2.77(2.45)	1.11(0.94-1.31)	0.24
AK7	*MAGI2*	v_23_38	1.93(1.82)	2.75(2.34)	1.16(0.95-1.40)	0.14
AK7	*CERKL*	v_23_39	1.36(1.05)	1.07(0.54)	0.62(0.29-1.32)	0.22
AK7	*CDK4*	v_23_40	1.34(1.00)	1.08(0.62)	0.71(0.39-1.29)	0.26
ULK4	*WNK1*	v_24_25	1.71(1.69)	2.25(2.07)	1.15(0.94-1.40)	0.18
ULK4	*CDC7*	v_24_26	1.46(0.98)	1.14(0.79)	0.80(0.48-1.33)	0.38
ULK4	*DGKB*	v_24_28	1.85(1.69)	1.27(0.90)	0.83(0.58-1.18)	0.30
ULK4	*PTK2*	v_24_29	1.65(1.56)	2.09(2.19)	1.14(0.91-1.42)	0.27
ULK4	*TRIB1*	v_24_30	1.71(2.19)	1.84(1.59)	1.01(0.83-1.21)	0.95
ULK4	*PRKCB*	v_24_33	1.56(2.06)	1.22(0.84)	0.93(0.67-1.30)	0.68
ULK4	*MYLK*	v_24_34	2.60(4.04)	2.45(2.45)	1.01(0.91-1.13)	0.81
ULK4	*MAP2K1*	v_24_36	1.96(1.95)	2.10(2.00)	1.07(0.87-1.30)	0.52
ULK4	*SGK3*	v_24_37	2.33(2.75)	3.13(3.00)	1.07(0.95-1.22)	0.25
ULK4	*MAGI2*	v_24_38	2.22(2.44)	3.42(4.11)	1.12(0.99-1.26)	0.08
ULK4	*CERKL*	v_24_39	1.17(0.51)	1.02(0.44)	0.72(0.27-1.90)	0.50

ULK4	*CDK4*	v_24_40	1.34(0.89)	1.22(0.99)	1.01(0.59-1.72)	0.98
WNK1	*CDC7*	v_25_26	1.50(1.81)	1.26(1.60)	0.90(0.67-1.22)	0.51
WNK1	*DGKB*	v_25_28	2.13(4.53)	0.92(0.77)	0.72(0.43-1.19)	0.20
WNK1	*PTK2*	v_25_29	1.13(0.58)	1.16(0.93)	0.72(0.33-1.57)	0.40
WNK1	*TRIB1*	v_25_30	1.57(1.68)	1.04(0.70)	0.63(0.39-1.02)	0.06
WNK1	*PRKCB*	v_25_33	1.88(2.49)	1.53(2.81)	0.94(0.75-1.17)	0.58
WNK1	*MYLK*	v_25_34	2.15(3.69)	1.66(2.23)	0.97(0.81-1.17)	0.77
WNK1	*MAP2K1*	v_25_36	1.89(2.85)	1.56(2.08)	0.97(0.80-1.17)	0.75
WNK1	*SGK3*	v_25_37	1.36(0.88)	1.30(0.69)	0.79(0.43-1.43)	0.43
WNK1	*MAGI2*	v_25_38	1.29(0.70)	1.48(1.00)	1.37(0.80-2.33)	0.25
WNK1	*CERKL*	v_25_39	1.86(2.84)	1.19(1.65)	0.83(0.60-1.15)	0.26
WNK1	*CDK4*	v_25_40	1.94(4.27)	1.92(4.10)	1.00(0.88-1.12)	0.96
CDC7	*PRKCB*	v_26_33	2.18(3.58)	2.53(5.41)	0.99(0.89-1.09)	0.76
CDC7	*CERKL*	v_26_39	1.85(2.73)	1.66(1.97)	0.92(0.76-1.12)	0.42
CDC7	*CDK4*	v_26_40	1.47(1.74)	1.72(1.99)	0.99(0.79-1.23)	0.90
DGKB	*PTK2*	v_28_29	1.66(2.19)	2.17(2.75)	1.05(0.87-1.28)	0.60
DGKB	*TRIB1*	v_28_30	2.31(3.93)	2.08(2.78)	0.95(0.82-1.10)	0.48
DGKB	*MYLK*	v_28_34	2.25(2.88)	2.73(3.97)	1.06(0.92-1.22)	0.40
DGKB	*MAP2K1*	v_28_36	1.52(1.15)	1.64(0.98)	1.05(0.71-1.56)	0.79
DGKB	*SGK3*	v_28_37	1.90(2.26)	3.46(6.18)	1.12(1.02-1.24)	0.02
DGKB	*MAGI2*	v_28_38	2.04(4.03)	3.00(3.65)	1.08(0.98-1.17)	0.11
PTK2	*TRIB1*	v_29_30	1.44(1.38)	1.33(1.27)	0.95(0.63-1.44)	0.82
PTK2	*PRKCB*	v_29_33	1.68(1.98)	1.23(1.25)	0.91(0.65-1.27)	0.58
PTK2	*MYLK*	v_29_34	2.06(2.56)	2.93(6.49)	1.12(1.01-1.25)	0.03
PTK2	*MAP2K1*	v_29_36	1.93(2.57)	2.46(5.15)	1.10(0.97-1.25)	0.14
PTK2	*SGK3*	v_29_37	1.35(0.83)	1.57(1.02)	1.43(0.86-2.40)	0.17
PTK2	*MAGI2*	v_29_38	1.35(0.81)	1.70(1.25)	1.57(1.07-2.31)	0.02
PTK2	*CERKL*	v_29_39	1.70(2.05)	1.45(2.24)	0.98(0.76-1.28)	0.90
PTK2	*CDK4*	v_29_40	1.58(2.11)	1.35(1.69)	0.98(0.75-1.28)	0.87

TRIB1	*SGK3*	v_30_37	1.40(0.95)	1.68(1.30)	1.31(0.85-2.00)	0.22
TRIB1	*MAGI2*	v_30_38	1.69(2.26)	1.88(1.55)	1.04(0.88-1.25)	0.63
PGK1	*STK32A*	v_31_32	1.41(1.20)	1.35(1.08)	0.82(0.52-1.31)	0.42
PGK1	*MAP2K1*	v_31_36	2.91(3.94)	2.52(2.37)	1.00(0.89-1.13)	0.94
PGK1	*SGK3*	v_31_37	2.51(2.29)	2.60(2.52)	1.00(0.81-1.23)	0.99
PGK1	*MAGI2*	v_31_38	2.20(1.82)	2.85(3.30)	1.08(0.91-1.27)	0.39
PGK1	*CERKL*	v_31_39	2.42(3.51)	1.62(2.15)	0.90(0.70-1.15)	0.40
STK32A	*PRKCB*	v_32_33	2.72(4.22)	2.60(3.49)	1.01(0.91-1.12)	0.90
STK32A	*MAGI2*	v_32_38	2.67(2.49)	4.47(5.63)	1.14(1.03-1.26)	0.01
STK32A	*CERKL*	v_32_39	2.12(2.45)	2.63(4.23)	1.07(0.94-1.23)	0.32
STK32A	*CDK4*	v_32_40	2.44(3.94)	2.38(3.21)	1.00(0.89-1.13)	0.94
PRKCB	*MYLK*	v_33_34	2.11(2.77)	2.65(2.91)	1.09(0.96-1.23)	0.19
PRKCB	*MAP2K1*	v_33_36	1.52(1.11)	2.01(2.20)	1.43(1.06-1.94)	0.02
PRKCB	*SGK3*	v_33_37	1.73(1.50)	2.47(2.03)	1.31(1.01-1.70)	0.04
PRKCB	*MAGI2*	v_33_38	1.69(1.78)	2.63(2.38)	1.24(1.04-1.49)	0.02
PRKCB	*CERKL*	v_33_39	1.21(1.05)	1.10(0.75)	0.87(0.49-1.52)	0.61
PRKCB	*CDK4*	v_33_40	1.23(0.96)	1.05(0.53)	0.80(0.44-1.46)	0.46
MYLK	*PI4K2B*	v_34_35	1.93(2.52)	1.89(2.00)	0.91(0.73-1.13)	0.40
MAP2K1	*SGK3*	v_36_37	1.78(2.07)	3.73(6.85)	1.11(1.02-1.20)	0.02
MAP2K1	*CDK4*	v_36_40	1.53(2.27)	1.46(1.59)	0.96(0.75-1.23)	0.73
SGK3	*MAGI2*	v_37_38	1.67(2.04)	1.71(1.78)	1.03(0.84-1.26)	0.77
CERKL	*CDK4*	v_39_40	1.15(0.60)	1.24(0.82)	1.45(0.67-3.16)	0.35
			Replication set			
MAP4K1	*STK39*	v_1_3	1.21(0.80)	1.27(0.95)	1.08(0.65-1.79)	0.78
MAP4K1	*DOLK*	v_1_12	1.32(1.23)	2.83(5.51)	1.25(1.07-1.47)	0.01
MAP4K1	*NEK4*	v_1_14	1.40(1.50)	2.13(3.23)	1.18(0.98-1.43)	0.08
MAP4K1	*RIOK2*	v_1_27	1.48(1.30)	3.07(6.33)	1.22(1.06-1.40)	0.005
MAP4K1	*PTK2*	v_1_29	1.39(1.48)	2.53(3.96)	1.19(1.03-1.38)	0.02
CDKL4	*STK39*	v_2_3	1.11(0.26)	0.94(0.29)	0.19(0.04-0.95)	0.04

CDKL4	*PRKD3*	v_2_4	1.23(0.87)	1.21(0.70)	0.91(0.50-1.65)	0.75
CDKL4	*AGK*	v_2_5	1.26(1.14)	1.22(0.59)	0.96(0.62-1.49)	0.85
CDKL4	*PIK3CA*	v_2_6	1.60(1.38)	1.16(0.84)	0.72(0.44-1.17)	0.18
CDKL4	*PKN3*	v_2_7	1.45(1.10)	1.53(1.23)	1.02(0.69-1.49)	0.94
CDKL4	*MST1R*	v_2_9	1.18(0.70)	1.44(1.06)	1.66(0.93-2.95)	0.09
CDKL4	*NEK8*	v_2_10	1.28(0.89)	1.46(1.02)	1.20(0.74-1.94)	0.46
CDKL4	*SPHK2*	v_2_11	1.25(0.85)	1.49(1.59)	1.37(0.89-2.10)	0.15
CDKL4	*BMPR1A*	v_2_13	1.39(1.47)	1.56(1.38)	1.09(0.81-1.45)	0.57
CDKL4	*NEK4*	v_2_14	1.28(0.86)	1.28(1.06)	1.08(0.64-1.82)	0.77
CDKL4	*BMPR1B*	v_2_16	1.25(0.91)	1.67(1.69)	1.41(0.97-2.05)	0.07
CDKL4	*DYRK4*	v_2_17	1.24(0.93)	1.84(2.47)	1.46(1.09-1.96)	0.01
CDKL4	*LIMK2*	v_2_18	1.22(0.80)	1.74(1.69)	1.66(1.13-2.46)	0.01
CDKL4	*AK7*	v_2_23	1.24(0.79)	1.39(1.48)	1.28(0.79-2.08)	0.31
CDKL4	*WNK1*	v_2_25	1.33(1.13)	1.61(2.13)	1.23(0.90-1.68)	0.19
CDKL4	*CDC7*	v_2_26	1.48(1.67)	2.29(4.15)	1.17(0.99-1.38)	0.07
CDKL4	*PTK2*	v_2_29	1.23(0.85)	1.61(1.41)	1.45(0.97-2.19)	0.07
CDKL4	*TRIB1*	v_2_30	1.51(1.77)	3.11(5.28)	1.24(1.08-1.43)	0.003
CDKL4	*MYLK*	v_2_34	1.20(0.73)	1.74(2.06)	1.69(1.16-2.46)	0.01
CDKL4	*MAP2K1*	v_2_36	1.30(0.83)	1.93(2.54)	1.52(1.12-2.07)	0.01
CDKL4	*SGK3*	v_2_37	1.23(0.80)	1.60(1.64)	1.47(1.00-2.17)	0.05
CDKL4	*MAGI2*	v_2_38	1.42(1.35)	2.03(2.21)	1.26(1.00-1.59)	0.05
CDKL4	*CERKL*	v_2_39	1.18(0.66)	1.43(1.22)	1.53(0.89-2.64)	0.13
STK39	*PRKD3*	v_3_4	1.18(0.90)	1.32(0.69)	1.09(0.66-1.78)	0.74
STK39	*AGK*	v_3_5	1.18(1.02)	1.29(0.51)	1.07(0.72-1.60)	0.74
STK39	*PKN3*	v_3_7	1.34(1.02)	1.70(1.54)	1.12(0.82-1.54)	0.47
STK39	*MST1R*	v_3_9	1.19(1.07)	1.56(1.27)	1.27(0.89-1.82)	0.18
STK39	*NEK8*	v_3_10	1.30(1.26)	1.60(1.18)	1.14(0.82-1.59)	0.42
STK39	*SPHK2*	v_3_11	1.28(1.31)	1.67(2.01)	1.19(0.90-1.57)	0.22
STK39	*BMPR1A*	v_3_13	1.54(2.37)	1.71(1.59)	1.02(0.85-1.24)	0.81

STK39	*NEK4*	v_3_14	1.30(1.34)	1.42(1.22)	1.06(0.76-1.47)	0.73
STK39	*BMPR1B*	v_3_16	1.30(1.46)	1.88(2.11)	1.19(0.93-1.52)	0.16
STK39	*DYRK4*	v_3_17	1.26(1.36)	2.10(3.08)	1.28(1.05-1.56)	0.02
STK39	*LIMK2*	v_3_18	1.19(1.06)	1.88(1.97)	1.43(1.08-1.90)	0.01
STK39	*AK7*	v_3_23	1.25(1.25)	1.60(1.84)	1.19(0.89-1.59)	0.24
STK39	*WNK1*	v_3_25	1.41(1.84)	1.84(2.63)	1.12(0.92-1.36)	0.27
STK39	*CDC7*	v_3_26	1.64(2.67)	2.67(5.20)	1.09(0.97-1.23)	0.15
STK39	*PTK2*	v_3_29	1.24(1.23)	1.71(1.52)	1.25(0.93-1.66)	0.13
STK39	*TRIB1*	v_3_30	1.67(2.72)	3.62(6.69)	1.14(1.04-1.25)	0.01
STK39	*MYLK*	v_3_34	1.19(1.04)	1.94(2.59)	1.41(1.10-1.81)	0.01
STK39	*MAP2K1*	v_3_36	1.28(1.14)	2.21(3.17)	1.36(1.09-1.69)	0.01
STK39	*SGK3*	v_3_37	1.23(1.21)	1.79(1.93)	1.29(0.98-1.69)	0.07
STK39	*MAGI2*	v_3_38	1.50(2.17)	2.28(2.75)	1.12(0.97-1.31)	0.13
STK39	*CERKL*	v_3_39	1.18(0.97)	1.60(1.44)	1.34(0.93-1.94)	0.12
STK39	*CDK4*	v_3_40	1.47(1.96)	2.32(3.53)	1.16(0.99-1.35)	0.07
PRKD3	*AGK*	v_4_5	1.28(0.89)	1.11(0.44)	0.89(0.43-1.83)	0.76
PRKD3	*WNK1*	v_4_25	1.65(2.14)	1.93(3.56)	1.14(0.94-1.39)	0.17
PRKD3	*SGK3*	v_4_37	1.50(1.62)	1.69(2.16)	1.21(0.90-1.62)	0.21
AGK	*DYRK4*	v_5_17	1.60(3.21)	1.55(1.82)	0.98(0.85-1.15)	0.84
AGK	*LIMK2*	v_5_18	1.50(2.42)	1.46(1.23)	0.98(0.80-1.20)	0.82
AGK	*PTK2*	v_5_29	1.63(3.35)	1.31(0.96)	0.95(0.77-1.17)	0.61
AGK	*MYLK*	v_5_34	1.55(2.85)	1.45(1.51)	0.98(0.82-1.17)	0.80
AGK	*SGK3*	v_5_37	1.93(4.93)	1.34(1.17)	0.95(0.81-1.13)	0.58
AGK	*MAGI2*	v_5_38	1.97(4.61)	1.69(1.64)	0.98(0.86-1.11)	0.70
HK2	*SPHK2*	v_8_11	1.09(0.46)	1.07(0.45)	0.93(0.36-2.40)	0.88
HK2	*BMPR1A*	v_8_13	1.07(0.47)	1.07(0.34)	1.01(0.35-2.89)	0.98
HK2	*NEK4*	v_8_14	1.17(0.59)	1.04(0.61)	0.77(0.33-1.78)	0.54
HK2	*PRKCD*	v_8_15	1.08(0.41)	1.04(0.34)	0.74(0.23-2.33)	0.61
HK2	*BMPR1B*	v_8_16	1.05(0.43)	1.09(0.32)	1.03(0.37-2.87)	0.95

HK2	*DYRK4*	v_8_17	1.08(0.52)	1.18(0.55)	1.31(0.59-2.92)	0.51
HK2	*LIMK2*	v_8_18	1.14(0.89)	1.16(0.46)	0.99(0.61-1.60)	0.96
HK2	*BMP2K*	v_8_22	1.09(0.55)	1.17(0.55)	1.22(0.63-2.36)	0.55
HK2	*WNK1*	v_8_25	1.17(0.70)	1.01(0.51)	0.73(0.30-1.76)	0.48
HK2	*CDC7*	v_8_26	1.15(0.57)	1.07(0.62)	0.76(0.33-1.76)	0.52
HK2	*PTK2*	v_8_29	1.10(0.53)	1.10(0.44)	0.94(0.38-2.31)	0.88
HK2	*TRIB1*	v_8_30	1.08(0.53)	1.34(0.98)	1.79(0.97-3.32)	0.06
HK2	*MYLK*	v_8_34	1.10(0.54)	1.17(0.48)	1.19(0.51-2.75)	0.69
HK2	*PI4K2B*	v_8_35	1.09(0.52)	1.23(0.80)	1.32(0.63-2.78)	0.46
HK2	*SGK3*	v_8_37	1.17(0.73)	1.08(0.53)	0.81(0.40-1.66)	0.57
HK2	*MAGI2*	v_8_38	1.19(0.54)	1.27(0.42)	1.17(0.52-2.66)	0.70
MST1R	*SPHK2*	v_9_11	1.05(0.25)	1.03(0.32)	0.97(0.18-5.40)	0.98
MST1R	*BMPR1A*	v_9_13	1.10(0.56)	1.09(0.48)	0.93(0.41-2.11)	0.87
MST1R	*NEK4*	v_9_14	1.11(0.43)	0.95(0.28)	0.46(0.13-1.69)	0.24
MST1R	*BMPR1B*	v_9_16	1.04(0.33)	1.12(0.47)	1.35(0.40-4.55)	0.63
MST1R	*DYRK4*	v_9_17	1.07(0.47)	1.12(0.45)	1.28(0.45-3.61)	0.65
MST1R	*LIMK2*	v_9_18	1.08(0.55)	1.12(0.38)	1.11(0.52-2.36)	0.78
MST1R	*NME4*	v_9_19	1.23(0.73)	1.63(1.69)	1.68(1.11-2.55)	0.01
MST1R	*AK7*	v_9_23	1.13(0.52)	1.05(0.58)	0.83(0.33-2.10)	0.69
MST1R	*ULK4*	v_9_24	1.17(0.65)	1.19(0.85)	1.01(0.52-1.95)	0.99
MST1R	*WNK1*	v_9_25	1.14(0.52)	1.02(0.52)	0.71(0.27-1.84)	0.48
MST1R	*CDC7*	v_9_26	1.18(0.63)	1.17(0.99)	0.95(0.47-1.91)	0.88
MST1R	*DGKB*	v_9_28	1.26(1.16)	1.50(1.24)	1.17(0.85-1.62)	0.34
MST1R	*PTK2*	v_9_29	1.11(0.54)	1.07(0.38)	0.77(0.31-1.93)	0.58
MST1R	*TRIB1*	v_9_30	1.14(0.69)	1.72(2.21)	1.47(1.11-1.94)	0.01
MST1R	*MYLK*	v_9_34	1.08(0.49)	1.14(0.41)	1.18(0.46-3.01)	0.73
MST1R	*MAP2K1*	v_9_36	1.22(0.91)	1.20(0.65)	1.00(0.61-1.67)	0.99
MST1R	*SGK3*	v_9_37	1.12(0.49)	1.11(0.72)	0.98(0.38-2.55)	0.96
MST1R	*MAGI2*	v_9_38	1.19(0.56)	1.25(0.40)	1.20(0.51-2.83)	0.68

MST1R	*CERKL*	v_9_39	1.12(0.57)	1.05(0.40)	0.73(0.29-1.81)	0.49
MST1R	*CDK4*	v_9_40	1.18(0.51)	1.23(0.80)	1.21(0.55-2.69)	0.64
NEK8	*NEK4*	v_10_14	1.53(1.84)	0.94(0.40)	0.63(0.31-1.30)	0.21
NEK8	*BMPR1B*	v_10_16	1.35(1.28)	1.16(0.71)	0.84(0.50-1.43)	0.53
NEK8	*LIMK2*	v_10_18	1.31(1.37)	1.20(0.75)	0.94(0.61-1.46)	0.79
NEK8	*NME4*	v_10_19	1.54(1.84)	1.80(2.04)	1.12(0.92-1.38)	0.26
NEK8	*AK7*	v_10_23	1.37(1.30)	0.99(0.50)	0.67(0.33-1.38)	0.28
NEK8	*WNK1*	v_10_25	1.66(2.46)	1.05(0.70)	0.76(0.44-1.31)	0.32
NEK8	*CDC7*	v_10_26	1.67(2.08)	1.18(1.14)	0.83(0.56-1.22)	0.34
NEK8	*PTK2*	v_10_29	1.41(1.35)	1.11(0.69)	0.78(0.46-1.32)	0.36
NEK8	*PRKCB*	v_10_33	1.28(1.22)	1.11(0.60)	0.89(0.52-1.52)	0.67
NEK8	*MYLK*	v_10_34	1.35(1.33)	1.16(0.62)	0.86(0.51-1.42)	0.55
NEK8	*MAP2K1*	v_10_36	1.27(0.94)	1.17(0.70)	0.91(0.52-1.57)	0.73
NEK8	*CERKL*	v_10_39	1.50(1.99)	1.06(0.56)	0.74(0.43-1.30)	0.30
SPHK2	*BMPR1A*	v_11_13	1.13(0.68)	1.10(0.41)	0.85(0.40-1.81)	0.67
SPHK2	*NEK4*	v_11_14	1.15(0.64)	0.96(0.27)	0.55(0.18-1.69)	0.30
SPHK2	*BMPR1B*	v_11_16	1.03(0.33)	1.10(0.35)	1.16(0.29-4.63)	0.83
SPHK2	*DYRK4*	v_11_17	1.08(0.49)	1.14(0.42)	1.24(0.45-3.45)	0.68
SPHK2	*LIMK2*	v_11_18	1.04(0.44)	1.17(0.58)	1.41(0.63-3.14)	0.40
SPHK2	*LYN*	v_11_20	1.22(1.14)	1.23(0.71)	0.97(0.64-1.49)	0.90
SPHK2	*WNK1*	v_11_25	1.18(0.78)	1.01(0.42)	0.64(0.25-1.67)	0.36
SPHK2	*PTK2*	v_11_29	1.11(0.57)	1.10(0.44)	0.79(0.33-1.90)	0.60
SPHK2	*SGK3*	v_11_37	1.13(0.58)	1.09(0.58)	0.78(0.34-1.80)	0.57
DOLK	*BMPR1A*	v_12_13	1.24(1.19)	1.02(0.37)	0.74(0.34-1.61)	0.45
DOLK	*NEK4*	v_12_14	1.25(1.18)	0.92(0.31)	0.42(0.11-1.67)	0.22
DOLK	*BMPR1B*	v_12_16	1.05(0.29)	1.01(0.28)	0.59(0.12-3.01)	0.53
DOLK	*DYRK4*	v_12_17	1.17(0.81)	1.08(0.40)	0.87(0.40-1.90)	0.72
DOLK	*LIMK2*	v_12_18	1.08(0.50)	1.08(0.47)	1.01(0.43-2.36)	0.99
DOLK	*NME4*	v_12_19	1.18(0.59)	1.46(1.09)	1.88(1.03-3.44)	0.04

DOLK	*AK7*	v_12_23	1.20(0.75)	1.00(0.56)	0.66(0.26-1.67)	0.39
DOLK	*WNK1*	v_12_25	1.33(1.72)	0.94(0.38)	0.51(0.16-1.66)	0.27
DOLK	*RIOK2*	v_12_27	1.21(0.48)	1.04(0.66)	0.65(0.25-1.69)	0.38
DOLK	*PTK2*	v_12_29	1.16(0.65)	1.02(0.35)	0.62(0.23-1.63)	0.33
DOLK	*TRIB1*	v_12_30	1.19(0.84)	1.39(1.40)	1.32(0.85-2.06)	0.22
DOLK	*PGK1*	v_12_31	1.20(0.60)	1.00(0.41)	0.53(0.20-1.36)	0.19
DOLK	*STK32A*	v_12_32	1.96(2.74)	1.43(1.58)	0.91(0.73-1.15)	0.45
DOLK	*SGK3*	v_12_37	1.15(0.56)	1.01(0.49)	0.59(0.20-1.68)	0.32
DOLK	*MAGI2*	v_12_38	1.21(0.63)	1.16(0.22)	0.87(0.36-2.12)	0.76
DOLK	*CERKL*	v_12_39	1.28(1.41)	0.99(0.29)	0.61(0.22-1.65)	0.33
DOLK	*CDK4*	v_12_40	1.28(1.01)	1.07(0.48)	0.71(0.30-1.65)	0.42
BMPR1A	*NEK4*	v_13_14	1.14(0.57)	0.97(0.38)	0.58(0.19-1.81)	0.35
BMPR1A	*PRKCD*	v_13_15	1.10(0.44)	0.99(0.23)	0.54(0.16-1.87)	0.33
BMPR1A	*BMPR1B*	v_13_16	1.05(0.33)	1.05(0.24)	0.88(0.22-3.56)	0.86
BMPR1A	*DYRK4*	v_13_17	1.10(0.56)	1.18(0.59)	1.32(0.57-3.05)	0.52
BMPR1A	*LIMK2*	v_13_18	1.19(0.81)	1.19(0.60)	0.99(0.57-1.70)	0.96
BMPR1A	*WNK1*	v_13_25	1.14(0.49)	1.00(0.49)	0.65(0.22-1.97)	0.45
BMPR1A	*PTK2*	v_13_29	1.15(0.67)	1.06(0.36)	0.75(0.32-1.76)	0.51
BMPR1A	*SGK3*	v_13_37	1.21(0.73)	1.01(0.33)	0.65(0.29-1.45)	0.29
BMPR1A	*MAGI2*	v_13_38	1.26(0.66)	1.29(0.55)	1.03(0.52-2.03)	0.94
NEK4	*BMPR1B*	v_14_16	1.03(0.45)	1.21(0.46)	1.31(0.58-2.95)	0.51
NEK4	*DYRK4*	v_14_17	1.06(0.51)	1.24(0.53)	1.60(0.65-3.93)	0.30
NEK4	*LIMK2*	v_14_18	1.09(0.60)	1.30(0.68)	1.41(0.73-2.74)	0.31
NEK4	*NME4*	v_14_19	1.23(0.86)	1.85(1.99)	1.60(1.17-2.18)	0.003
NEK4	*AK7*	v_14_23	1.07(0.45)	1.10(0.45)	1.10(0.38-3.21)	0.86
NEK4	*WNK1*	v_14_25	1.07(0.45)	1.08(0.49)	0.89(0.35-2.28)	0.80
NEK4	*CDC7*	v_14_26	1.11(0.55)	1.24(1.03)	1.15(0.55-2.43)	0.71
NEK4	*DGKB*	v_14_28	1.21(0.91)	1.69(1.42)	1.47(1.01-2.13)	0.05
NEK4	*PTK2*	v_14_29	1.09(0.61)	1.18(0.46)	1.00(0.48-2.06)	1.00

NEK4	*TRIB1*	v_14_30	1.08(0.66)	1.90(2.38)	1.48(1.16-1.89)	0.002
NEK4	*PRKCB*	v_14_33	1.13(0.66)	1.22(0.45)	1.23(0.59-2.59)	0.58
NEK4	*MYLK*	v_14_34	1.08(0.63)	1.26(0.46)	1.20(0.63-2.27)	0.57
NEK4	*MAP2K1*	v_14_36	1.16(0.74)	1.30(0.66)	1.23(0.70-2.15)	0.48
NEK4	*SGK3*	v_14_37	1.10(0.54)	1.18(0.72)	1.09(0.46-2.59)	0.84
NEK4	*MAGI2*	v_14_38	1.19(0.63)	1.40(0.54)	1.41(0.68-2.94)	0.35
NEK4	*CERKL*	v_14_39	1.06(0.53)	1.13(0.32)	0.92(0.40-2.08)	0.83
NEK4	*CDK4*	v_14_40	1.14(0.52)	1.34(0.88)	1.44(0.72-2.88)	0.31
PRKCD	*BMPR1B*	v_15_16	1.01(0.27)	1.08(0.22)	2.28(0.35-14.65)	0.39
PRKCD	*LIMK2*	v_15_18	1.09(0.54)	1.21(0.59)	1.36(0.65-2.85)	0.41
PRKCD	*BMP2K*	v_15_22	1.07(0.45)	1.23(0.76)	2.18(0.92-5.16)	0.08
PRKCD	*WNK1*	v_15_25	1.09(0.41)	0.99(0.34)	0.66(0.14-3.03)	0.60
PRKCD	*PTK2*	v_15_29	1.06(0.40)	1.11(0.41)	1.26(0.40-3.92)	0.69
PRKCD	*MYLK*	v_15_34	1.07(0.50)	1.16(0.33)	1.52(0.57-4.10)	0.40
PRKCD	*PI4K2B*	v_15_35	1.08(0.53)	1.24(0.75)	1.54(0.71-3.33)	0.27
PRKCD	*SGK3*	v_15_37	1.10(0.53)	1.04(0.33)	0.80(0.31-2.07)	0.64
PRKCD	*MAGI2*	v_15_38	1.15(0.47)	1.29(0.41)	1.68(0.64-4.43)	0.30
BMPR1B	*LIMK2*	v_16_18	1.09(0.51)	1.15(0.59)	1.21(0.55-2.66)	0.63
BMPR1B	*BMP2K*	v_16_22	1.09(0.45)	1.16(0.67)	1.73(0.68-4.41)	0.25
BMPR1B	*CDC7*	v_16_26	1.18(0.79)	1.01(0.60)	0.66(0.25-1.78)	0.42
BMPR1B	*PTK2*	v_16_29	1.12(0.55)	1.04(0.39)	0.74(0.27-2.00)	0.55
BMPR1B	*MYLK*	v_16_34	1.11(0.57)	1.10(0.33)	1.04(0.40-2.68)	0.94
BMPR1B	*PI4K2B*	v_16_35	1.12(0.61)	1.16(0.67)	1.05(0.48-2.30)	0.89
DYRK4	*LIMK2*	v_17_18	1.06(0.40)	1.11(0.49)	1.35(0.45-4.04)	0.60
DYRK4	*NME4*	v_17_19	1.20(0.66)	1.38(0.84)	1.75(0.94-3.26)	0.08
DYRK4	*AK7*	v_17_23	1.13(0.53)	0.98(0.51)	0.62(0.21-1.81)	0.38
DYRK4	*WNK1*	v_17_25	1.15(0.49)	0.93(0.36)	0.35(0.10-1.24)	0.10
DYRK4	*CDC7*	v_17_26	1.21(0.72)	1.01(0.58)	0.59(0.25-1.36)	0.21
DYRK4	*DGKB*	v_17_28	1.31(1.09)	1.44(1.17)	1.10(0.75-1.60)	0.64

DYRK4	*PTK2*	v_17_29	1.09(0.54)	1.11(0.74)	0.94(0.43-2.05)	0.88
DYRK4	*TRIB1*	v_17_30	1.14(0.69)	1.61(2.06)	1.40(1.03-1.90)	0.03
DYRK4	*PRKCB*	v_17_33	1.20(0.82)	1.09(0.56)	0.87(0.46-1.63)	0.65
DYRK4	*MYLK*	v_17_34	1.11(0.58)	1.12(0.54)	0.97(0.42-2.21)	0.94
DYRK4	*MAP2K1*	v_17_36	1.31(1.02)	1.16(0.76)	0.88(0.54-1.45)	0.62
DYRK4	*SGK3*	v_17_37	1.22(0.80)	1.11(0.81)	0.81(0.42-1.55)	0.52
DYRK4	*MAGI2*	v_17_38	1.27(0.76)	1.26(0.71)	0.95(0.51-1.75)	0.86
DYRK4	*CERKL*	v_17_39	1.15(0.61)	1.01(0.38)	0.57(0.21-1.54)	0.27
DYRK4	*CDK4*	v_17_40	1.24(0.68)	1.13(0.68)	0.80(0.38-1.65)	0.54
LIMK2	*BMP2K*	v_18_22	1.15(0.71)	1.11(0.60)	0.99(0.48-2.05)	0.97
LIMK2	*PI4K2B*	v_18_35	1.29(0.97)	1.23(0.94)	0.85(0.52-1.40)	0.53
LYN	*CDC7*	v_20_26	1.14(0.48)	0.95(0.34)	0.34(0.09-1.21)	0.10
LYN	*DGKB*	v_20_28	1.33(1.12)	1.30(0.77)	1.01(0.64-1.61)	0.95
LYN	*PTK2*	v_20_29	1.11(0.48)	1.17(0.72)	1.09(0.48-2.45)	0.84
LYN	*TRIB1*	v_20_30	1.09(0.51)	1.34(1.29)	1.62(0.97-2.69)	0.06
LYN	*PRKCB*	v_20_33	1.27(0.87)	1.19(0.73)	0.95(0.53-1.70)	0.86
LYN	*MYLK*	v_20_34	1.22(0.84)	1.24(0.74)	1.03(0.57-1.84)	0.93
LYN	*MAP2K1*	v_20_36	1.42(1.14)	1.22(0.75)	0.88(0.55-1.43)	0.61
LYN	*SGK3*	v_20_37	1.24(0.76)	1.14(0.74)	0.85(0.44-1.68)	0.65
LYN	*MAGI2*	v_20_38	1.31(0.81)	1.31(0.62)	1.02(0.55-1.88)	0.96
LYN	*CDK4*	v_20_40	1.29(0.78)	1.20(0.66)	0.91(0.46-1.80)	0.79
NEK9	*PGK1*	v_21_31	1.32(0.99)	1.11(0.71)	0.78(0.44-1.38)	0.39
BMP2K	*PI4K2B*	v_22_35	1.19(0.81)	1.17(0.81)	0.79(0.43-1.45)	0.44
AK7	*ULK4*	v_23_24	1.08(0.46)	1.27(0.85)	1.67(0.74-3.74)	0.22
AK7	*WNK1*	v_23_25	1.09(0.45)	1.11(0.63)	1.04(0.42-2.57)	0.94
AK7	*CDC7*	v_23_26	1.15(0.62)	1.19(0.67)	0.96(0.46-.002)	0.92
AK7	*DGKB*	v_23_28	1.25(1.04)	1.72(1.16)	1.43(0.99-2.07)	0.06
AK7	*PTK2*	v_23_29	1.05(0.42)	1.29(0.76)	1.89(0.82-4.37)	0.14
AK7	*TRIB1*	v_23_30	1.13(0.71)	2.15(3.43)	1.29(1.10-1.52)	0.002

AK7	*PRKCB*	v_23_33	1.09(0.59)	1.23(0.47)	1.52(0.71-3.29)	0.28
AK7	*MYLK*	v_23_34	1.06(0.54)	1.28(0.59)	1.59(0.76-3.34)	0.22
AK7	*MAP2K1*	v_23_36	1.13(0.66)	1.32(0.70)	1.57(0.81-3.03)	0.18
AK7	*SGK3*	v_23_37	1.12(0.60)	1.34(1.24)	1.44(0.82-2.55)	0.21
AK7	*MAGI2*	v_23_38	1.20(0.69)	1.45(0.66)	1.45(0.79-2.63)	0.23
AK7	*CERKL*	v_23_39	1.08(0.55)	1.17(0.54)	1.07(0.48-2.38)	0.88
AK7	*CDK4*	v_23_40	1.17(0.57)	1.35(1.01)	1.47(0.75-2.85)	0.26
ULK4	*WNK1*	v_24_25	1.08(0.38)	0.97(0.26)	0.51(0.12-2.17)	0.36
ULK4	*CDC7*	v_24_26	1.07(0.32)	1.01(0.40)	0.60(0.14-2.55)	0.49
ULK4	*DGKB*	v_24_28	1.24(0.98)	1.50(1.14)	1.31(0.87-1.96)	0.19
ULK4	*PTK2*	v_24_29	1.09(0.54)	1.23(0.75)	1.39(0.66-2.93)	0.39
ULK4	*TRIB1*	v_24_30	1.07(0.56)	1.40(1.02)	2.21(1.22-4.01)	0.01
ULK4	*PRKCB*	v_24_33	1.19(0.80)	1.21(0.58)	1.11(0.62-2.01)	0.73
ULK4	*MYLK*	v_24_34	1.13(0.65)	1.28(0.67)	1.38(0.68-2.81)	0.37
ULK4	*MAP2K1*	v_24_36	1.24(0.78)	1.29(0.83)	1.17(0.65-2.10)	0.60
ULK4	*SGK3*	v_24_37	1.16(0.72)	1.15(0.66)	0.99(0.51-1.91)	0.97
ULK4	*MAGI2*	v_24_38	1.22(0.72)	1.39(0.70)	1.30(0.72-2.34)	0.38
ULK4	*CERKL*	v_24_39	1.10(0.50)	1.16(0.52)	1.11(0.45-2.77)	0.82
ULK4	*CDK4*	v_24_40	1.19(0.62)	1.23(0.72)	1.09(0.55-2.17)	0.80
WNK1	*CDC7*	v_25_26	1.06(0.37)	1.10(0.46)	0.98(0.30-3.22)	0.97
WNK1	*DGKB*	v_25_28	1.24(0.95)	1.63(1.20)	1.38(0.92-2.09)	0.12
WNK1	*PTK2*	v_25_29	1.05(0.42)	1.28(0.74)	1.95(0.82-4.63)	0.13
WNK1	*TRIB1*	v_25_30	1.05(0.55)	1.57(1.29)	2.27(1.42-3.62)	0.001
WNK1	*PRKCB*	v_25_33	1.12(0.57)	1.26(0.53)	1.39(0.65-2.99)	0.40
WNK1	*MYLK*	v_25_34	1.07(0.55)	1.33(0.64)	1.79(0.87-3.70)	0.12
WNK1	*MAP2K1*	v_25_36	1.17(0.69)	1.31(0.68)	1.27(0.67-2.40)	0.46
WNK1	*SGK3*	v_25_37	1.10(0.56)	1.18(0.60)	1.17(0.53-2.62)	0.69
WNK1	*MAGI2*	v_25_38	1.16(0.59)	1.45(0.68)	1.71(0.85-3.42)	0.13
WNK1	*CERKL*	v_25_39	1.04(0.40)	1.17(0.42)	1.54(0.52-4.57)	0.44

WNK1	*CDK4*	v_25_40	1.13(0.52)	1.28(0.75)	1.32(0.65-2.68)	0.44
CDC7	*PRKCB*	v_26_33	1.17(0.72)	1.28(0.62)	1.26(0.70-2.25)	0.45
CDC7	*CERKL*	v_26_39	1.07(0.47)	1.29(0.71)	1.67(0.77-3.64)	0.20
CDC7	*CDK4*	v_26_40	1.13(0.50)	1.37(1.04)	1.39(0.84-2.32)	0.20
DGKB	*PTK2*	v_28_29	1.48(1.74)	1.11(0.76)	0.79(0.48-1.29)	0.34
DGKB	*TRIB1*	v_28_30	1.53(2.27)	1.29(1.66)	0.95(0.70-1.27)	0.72
DGKB	*MYLK*	v_28_34	1.43(1.76)	1.23(0.97)	0.90(0.60-1.36)	0.61
DGKB	*MAP2K1*	v_28_36	1.28(1.06)	1.09(0.73)	0.83(0.44-1.56)	0.57
DGKB	*SGK3*	v_28_37	1.46(1.53)	1.09(0.86)	0.74(0.42-1.31)	0.30
DGKB	*MAGI2*	v_28_38	1.47(1.60)	1.24(0.75)	0.89(0.57-1.39)	0.60
PTK2	*TRIB1*	v_29_30	1.08(0.57)	1.48(1.59)	1.91(1.22-2.99)	0.005
PTK2	*PRKCB*	v_29_33	1.18(1.05)	1.11(0.42)	0.99(0.56-1.73)	0.96
PTK2	*MYLK*	v_29_34	1.06(0.44)	1.13(0.44)	1.71(0.57-5.17)	0.34
PTK2	*MAP2K1*	v_29_36	1.24(0.78)	1.20(0.67)	1.02(0.56-1.85)	0.95
PTK2	*SGK3*	v_29_37	1.11(0.52)	1.02(0.42)	0.75(0.27-2.05)	0.57
PTK2	*MAGI2*	v_29_38	1.21(0.62)	1.25(0.43)	1.17(0.53-2.56)	0.70
PTK2	*CERKL*	v_29_39	1.11(0.53)	1.06(0.39)	0.86(0.31-2.36)	0.77
PTK2	*CDK4*	v_29_40	1.19(0.50)	1.26(0.83)	1.32(0.59-2.96)	0.49
TRIB1	*SGK3*	v_30_37	1.25(0.82)	1.05(0.72)	0.70(0.33-1.50)	0.36
TRIB1	*MAGI2*	v_30_38	1.34(0.91)	1.30(0.72)	0.98(0.55-1.76)	0.96
PGK1	*STK32A*	v_31_32	1.78(2.54)	1.31(1.19)	0.90(0.69-1.17)	0.42
PGK1	*MAP2K1*	v_31_36	1.22(0.87)	1.24(0.74)	1.02(0.60-1.73)	0.94
PGK1	*SGK3*	v_31_37	1.58(3.58)	1.12(0.61)	0.92(0.64-1.32)	0.65
PGK1	*MAGI2*	v_31_38	1.68(3.88)	1.30(0.47)	0.96(0.76-1.21)	0.72
PGK1	*CERKL*	v_31_39	1.37(2.27)	1.15(0.58)	0.93(0.63-1.35)	0.69
STK32A	*PRKCB*	v_32_33	1.34(0.89)	1.31(0.79)	1.07(0.61-1.89)	0.82
STK32A	*MAGI2*	v_32_38	1.64(1.35)	1.50(0.94)	0.96(0.64-1.43)	0.84
STK32A	*CERKL*	v_32_39	1.77(2.46)	1.33(0.99)	0.88(0.63-1.24)	0.48
STK32A	*CDK4*	v_32_40	1.79(1.85)	1.55(1.47)	0.94(0.70-1.27)	0.69

PRKCB	*MYLK*	v_33_34	1.17(0.84)	1.09(0.41)	0.82(0.38-1.73)	0.60
PRKCB	*MAP2K1*	v_33_36	1.14(0.61)	1.11(0.51)	0.95(0.45-2.04)	0.90
PRKCB	*SGK3*	v_33_37	1.20(0.77)	1.06(0.81)	0.71(0.31-1.65)	0.43
PRKCB	*MAGI2*	v_33_38	1.26(0.91)	1.20(0.43)	0.90(0.48-1.69)	0.74
PRKCB	*CERKL*	v_33_39	1.45(2.18)	1.03(0.45)	0.73(0.36-1.48)	0.38
PRKCB	*CDK4*	v_33_40	1.40(1.49)	1.18(0.90)	0.86(0.53-1.42)	0.56
MYLK	*PI4K2B*	v_34_35	1.18(0.75)	1.17(0.78)	0.93(0.50-1.75)	0.83
MAP2K1	*SGK3*	v_36_37	1.29(1.18)	1.05(0.63)	0.73(0.36-1.45)	0.36
MAP2K1	*CDK4*	v_36_40	1.50(1.90)	1.09(0.69)	0.76(0.42-1.36)	0.35
SGK3	*MAGI2*	v_37_38	1.15(0.53)	1.35(0.55)	1.65(0.78-3.47)	0.19
CERKL	*CDK4*	v_39_40	1.16(0.50)	1.11(0.57)	0.92(0.36-2.31)	0.85
			Pooled set			
MAP4K1	*STK39*	v_1_3	1.75(1.81)	2.02(2.56)	1.09(0.93-1.27)	0.27
MAP4K1	*DOLK*	v_1_12	1.19(0.99)	2.34(4.30)	1.19(1.08-1.31)	<0.001
MAP4K1	*NEK4*	v_1_14	1.69(1.70)	2.44(3.31)	1.18(1.03-1.34)	0.02
MAP4K1	*RIOK2*	v_1_27	1.52(1.39)	2.36(4.61)	1.16(1.05-1.29)	0.003
MAP4K1	*PTK2*	v_1_29	1.83(2.34)	2.53(3.39)	1.10(0.99-1.21)	0.07
CDKL4	*STK39*	v_2_3	1.15(0.36)	1.08(0.52)	0.74(0.29-1.91)	0.54
CDKL4	*PRKD3*	v_2_4	1.61(1.30)	1.76(1.38)	1.15(0.89-1.48)	0.28
CDKL4	*AGK*	v_2_5	1.23(0.95)	1.32(0.65)	1.13(0.85-1.50)	0.40
CDKL4	*PIK3CA*	v_2_6	2.75(3.50)	1.32(1.08)	0.83(0.68-1.02)	0.07
CDKL4	*PKN3*	v_2_7	2.12(3.05)	2.11(2.66)	1.03(0.93-1.15)	0.53
CDKL4	*MST1R*	v_2_9	1.33(1.80)	1.53(2.44)	1.03(0.90-1.19)	0.63
CDKL4	*NEK8*	v_2_10	1.97(3.45)	1.58(1.75)	0.99(0.88-1.10)	0.80
CDKL4	*SPHK2*	v_2_11	1.47(1.22)	1.92(3.04)	1.16(1.03-1.31)	0.02
CDKL4	*BMPR1A*	v_2_13	1.46(1.44)	2.02(3.74)	1.10(1.00-1.22)	0.06
CDKL4	*NEK4*	v_2_14	1.16(0.63)	1.53(2.34)	1.18(1.00-1.39)	0.05
CDKL4	*BMPR1B*	v_2_16	1.39(1.46)	2.02(4.06)	1.10(1.01-1.21)	0.03
CDKL4	*DYRK4*	v_2_17	1.15(0.71)	1.55(1.88)	1.38(1.07-1.77)	0.01

CDKL4	*LIMK2*	v_2_18	1.43(1.49)	2.23(4.78)	1.10(1.02-1.18)	0.01
CDKL4	*AK7*	v_2_23	1.25(1.04)	1.71(3.38)	1.13(1.01-1.26)	0.04
CDKL4	*WNK1*	v_2_25	1.21(0.89)	1.46(1.54)	1.27(0.99-1.64)	0.06
CDKL4	*CDC7*	v_2_26	1.42(1.54)	2.07(3.71)	1.15(1.02-1.29)	0.02
CDKL4	*PTK2*	v_2_29	1.15(0.70)	1.60(1.85)	1.32(1.09-1.59)	0.004
CDKL4	*TRIB1*	v_2_30	1.42(1.38)	2.25(3.86)	1.17(1.05-1.32)	0.01
CDKL4	*MYLK*	v_2_34	1.67(2.03)	2.27(4.25)	1.10(1.01-1.19)	0.02
CDKL4	*MAP2K1*	v_2_36	1.61(2.15)	2.22(4.10)	1.09(1.00-1.19)	0.04
CDKL4	*SGK3*	v_2_37	1.33(0.89)	1.64(1.37)	1.44(1.05-1.96)	0.02
CDKL4	*MAGI2*	v_2_38	1.38(1.11)	1.91(1.77)	1.30(1.08-1.55)	0.005
CDKL4	*CERKL*	v_2_39	1.31(1.15)	1.57(2.39)	1.17(0.97-1.40)	0.09
STK39	*PRKD3*	v_3_4	1.43(1.03)	1.66(1.02)	1.22(0.91-1.62)	0.18
STK39	*AGK*	v_3_5	1.15(0.86)	1.30(0.68)	1.24(0.93-1.67)	0.15
STK39	*PKN3*	v_3_7	1.84(2.53)	2.10(2.53)	1.06(0.95-1.18)	0.31
STK39	*MST1R*	v_3_9	1.32(1.71)	1.36(1.30)	1.01(0.83-1.22)	0.95
STK39	*NEK8*	v_3_10	1.86(2.76)	1.63(1.76)	1.00(0.88-1.15)	0.95
STK39	*SPHK2*	v_3_11	1.54(2.19)	1.69(1.77)	1.05(0.92-1.20)	0.48
STK39	*BMPR1A*	v_3_13	1.68(3.03)	1.75(1.67)	1.01(0.90-1.13)	0.86
STK39	*NEK4*	v_3_14	1.16(1.02)	1.38(1.20)	1.10(0.86-1.41)	0.44
STK39	*BMPR1B*	v_3_16	1.45(2.01)	1.76(1.98)	1.08(0.94-1.25)	0.26
STK39	*DYRK4*	v_3_17	1.11(0.97)	1.57(2.23)	1.22(1.01-1.47)	0.04
STK39	*LIMK2*	v_3_18	1.50(2.09)	1.81(2.05)	1.10(0.96-1.25)	0.17
STK39	*AK7*	v_3_23	1.28(1.53)	1.51(1.78)	1.11(0.92-1.33)	0.29
STK39	*WNK1*	v_3_25	1.23(1.37)	1.56(1.92)	1.12(0.95-1.32)	0.18
STK39	*CDC7*	v_3_26	1.61(2.55)	2.11(3.92)	1.06(0.96-1.17)	0.23
STK39	*PTK2*	v_3_29	1.12(0.97)	1.49(1.27)	1.25(0.98-1.60)	0.07
STK39	*TRIB1*	v_3_30	1.48(2.01)	2.45(4.88)	1.11(1.01-1.21)	0.03
STK39	*MYLK*	v_3_34	1.76(2.87)	2.03(2.55)	1.06(0.97-1.16)	0.19
STK39	*MAP2K1*	v_3_36	1.71(3.05)	2.04(2.69)	1.06(0.97-1.16)	0.20

STK39	*SGK3*	v_3_37	1.23(0.97)	1.62(1.49)	1.26(1.00-1.59)	0.05
STK39	*MAGI2*	v_3_38	1.36(1.66)	1.91(2.02)	1.12(0.99-1.27)	0.07
STK39	*CERKL*	v_3_39	1.40(1.61)	1.49(1.60)	1.09(0.88-1.34)	0.43
STK39	*CDK4*	v_3_40	1.56(2.37)	2.08(3.51)	1.09(0.98-1.21)	0.13
PRKD3	*AGK*	v_4_5	1.62(2.55)	1.32(1.50)	0.94(0.80-1.11)	0.45
PRKD3	*WNK1*	v_4_25	1.82(3.04)	1.69(2.79)	1.00(0.88-1.15)	0.94
PRKD3	*SGK3*	v_4_37	1.53(2.01)	1.47(1.79)	0.97(0.82-1.15)	0.75
AGK	*DYRK4*	v_5_17	1.56(2.72)	1.25(1.37)	0.91(0.75-1.10)	0.34
AGK	*LIMK2*	v_5_18	1.69(2.52)	1.77(2.95)	1.00(0.89-1.12)	0.95
AGK	*PTK2*	v_5_29	1.52(2.68)	1.42(1.54)	0.95(0.83-1.09)	0.49
AGK	*MYLK*	v_5_34	1.94(3.01)	1.82(2.68)	0.98(0.89-1.09)	0.77
AGK	*SGK3*	v_5_37	1.54(3.35)	1.35(0.97)	0.96(0.84-1.09)	0.49
AGK	*MAGI2*	v_5_38	1.70(3.23)	1.64(1.48)	0.97(0.88-1.08)	0.60
HK2	*SPHK2*	v_8_11	1.32(0.95)	1.41(0.98)	1.18(0.82-1.69)	0.37
HK2	*BMPR1A*	v_8_13	1.17(0.73)	1.29(0.55)	1.19(0.76-1.85)	0.45
HK2	*NEK4*	v_8_14	1.51(1.83)	1.82(3.16)	1.07(0.94-1.21)	0.31
HK2	*PRKCD*	v_8_15	1.10(0.55)	1.22(0.56)	1.24(0.69-2.22)	0.47
HK2	*BMPR1B*	v_8_16	1.28(0.95)	1.26(0.71)	1.04(0.72-1.51)	0.84
HK2	*DYRK4*	v_8_17	1.50(1.79)	1.59(2.01)	1.05(0.87-1.25)	0.62
HK2	*LIMK2*	v_8_18	1.15(0.79)	1.25(0.55)	1.12(0.78-1.59)	0.54
HK2	*BMP2K*	v_8_22	1.20(0.75)	1.29(0.64)	1.15(0.79-1.68)	0.47
HK2	*WNK1*	v_8_25	1.52(1.94)	1.68(2.35)	1.07(0.92-1.24)	0.38
HK2	*CDC7*	v_8_26	1.55(2.21)	1.77(4.53)	1.04(0.94-1.15)	0.42
HK2	*PTK2*	v_8_29	1.42(1.66)	1.69(2.34)	1.10(0.94-1.28)	0.25
HK2	*TRIB1*	v_8_30	1.52(2.08)	2.16(3.56)	1.08(0.98-1.20)	0.13
HK2	*MYLK*	v_8_34	1.32(1.16)	1.28(0.84)	1.03(0.77-1.39)	0.82
HK2	*PI4K2B*	v_8_35	1.45(1.17)	1.84(1.83)	1.16(0.94-1.44)	0.17
HK2	*SGK3*	v_8_37	1.73(2.25)	2.20(3.52)	1.08(0.97-1.20)	0.18
HK2	*MAGI2*	v_8_38	1.83(2.80)	2.27(3.99)	1.06(0.97-1.16)	0.21

MST1R	*SPHK2*	v_9_11	1.55(1.44)	1.66(1.37)	1.19(0.95-1.50)	0.13
MST1R	*BMPR1A*	v_9_13	1.43(1.31)	1.74(1.65)	1.23(0.99-1.54)	0.06
MST1R	*NEK4*	v_9_14	1.31(0.82)	1.39(1.31)	1.17(0.85-1.59)	0.33
MST1R	*BMPR1B*	v_9_16	1.56(2.40)	1.55(1.28)	1.03(0.91-1.17)	0.66
MST1R	*DYRK4*	v_9_17	1.32(0.99)	1.46(1.12)	1.20(0.88-1.64)	0.25
MST1R	*LIMK2*	v_9_18	1.55(2.32)	1.62(1.24)	1.04(0.92-1.18)	0.53
MST1R	*NME4*	v_9_19	1.60(1.52)	1.85(2.30)	1.21(1.01-1.46)	0.04
MST1R	*AK7*	v_9_23	1.13(0.53)	1.11(0.52)	1.14(0.61-2.11)	0.68
MST1R	*ULK4*	v_9_24	1.21(0.73)	1.31(1.01)	1.14(0.77-1.69)	0.51
MST1R	*WNK1*	v_9_25	1.33(0.83)	1.56(1.26)	1.50(1.08-2.09)	0.02
MST1R	*CDC7*	v_9_26	1.34(0.88)	1.32(1.24)	1.09(0.76-1.56)	0.64
MST1R	*DGKB*	v_9_28	1.52(1.93)	1.38(1.07)	1.01(0.82-1.26)	0.90
MST1R	*PTK2*	v_9_29	1.31(0.89)	1.48(1.23)	1.24(0.92-1.69)	0.16
MST1R	*TRIB1*	v_9_30	1.39(1.08)	1.81(1.90)	1.27(1.02-1.58)	0.04
MST1R	*MYLK*	v_9_34	1.90(3.43)	1.99(2.33)	1.03(0.96-1.11)	0.42
MST1R	*MAP2K1*	v_9_36	1.50(1.25)	1.67(1.64)	1.27(1.01-1.61)	0.04
MST1R	*SGK3*	v_9_37	1.59(1.34)	1.98(2.19)	1.29(1.04-1.59)	0.02
MST1R	*MAGI2*	v_9_38	1.64(1.49)	2.03(2.03)	1.20(1.02-1.42)	0.03
MST1R	*CERKL*	v_9_39	1.25(0.92)	1.09(0.48)	0.86(0.54-1.38)	0.54
MST1R	*CDK4*	v_9_40	1.34(1.03)	1.26(0.85)	1.00(0.72-1.39)	1.00
NEK8	*NEK4*	v_10_14	2.01(2.86)	1.65(2.18)	0.92(0.78-1.08)	0.30
NEK8	*BMPR1B*	v_10_16	1.88(3.14)	1.81(2.69)	0.98(0.87-1.11)	0.79
NEK8	*LIMK2*	v_10_18	1.83(3.04)	1.77(2.43)	0.98(0.86-1.11)	0.73
NEK8	*NME4*	v_10_19	1.62(2.63)	1.60(1.59)	0.98(0.86-1.12)	0.80
NEK8	*AK7*	v_10_23	1.73(2.39)	1.20(1.00)	0.83(0.65-1.06)	0.13
NEK8	*WNK1*	v_10_25	1.90(2.83)	1.96(3.44)	1.00(0.89-1.13)	1.00
NEK8	*CDC7*	v_10_26	1.62(2.00)	1.21(1.05)	0.82(0.64-1.06)	0.13
NEK8	*PTK2*	v_10_29	1.84(2.68)	1.87(2.74)	0.97(0.85-1.12)	0.71
NEK8	*PRKCB*	v_10_33	1.82(2.89)	1.32(1.15)	0.87(0.71-1.08)	0.21

NEK8	*MYLK*	v_10_34	1.86(2.39)	2.15(3.93)	1.04(0.92-1.16)	0.55
NEK8	*MAP2K1*	v_10_36	1.60(2.16)	1.36(0.90)	0.91(0.72-1.14)	0.39
NEK8	*CERKL*	v_10_39	2.11(3.25)	1.30(1.49)	0.84(0.69-1.02)	0.08
SPHK2	*BMPR1A*	v_11_13	1.14(0.71)	1.12(0.45)	0.84(0.51-1.40)	0.51
SPHK2	*NEK4*	v_11_14	1.64(2.38)	1.29(1.53)	0.91(0.73-1.14)	0.41
SPHK2	*BMPR1B*	v_11_16	1.48(2.08)	1.11(0.53)	0.85(0.62-1.17)	0.31
SPHK2	*DYRK4*	v_11_17	1.85(2.98)	1.32(1.36)	0.89(0.73-1.10)	0.28
SPHK2	*LIMK2*	v_11_18	1.28(1.40)	1.13(0.61)	0.90(0.63-1.28)	0.55
SPHK2	*LYN*	v_11_20	1.77(3.24)	1.24(0.94)	0.90(0.72-1.13)	0.37
SPHK2	*WNK1*	v_11_25	1.80(2.99)	1.32(1.26)	0.92(0.75-1.12)	0.40
SPHK2	*PTK2*	v_11_29	1.57(2.29)	1.30(1.28)	0.91(0.72-1.15)	0.43
SPHK2	*SGK3*	v_11_37	1.69(2.51)	1.55(1.78)	0.98(0.82-1.15)	0.77
DOLK	*BMPR1A*	v_12_13	1.70(1.76)	2.00(2.98)	1.14(0.96-1.35)	0.13
DOLK	*NEK4*	v_12_14	1.60(1.34)	1.47(1.40)	0.96(0.72-1.29)	0.80
DOLK	*BMPR1B*	v_12_16	1.77(2.65)	1.70(2.46)	1.03(0.91-1.17)	0.65
DOLK	*DYRK4*	v_12_17	1.51(1.11)	1.65(1.78)	1.14(0.86-1.50)	0.37
DOLK	*LIMK2*	v_12_18	1.83(2.52)	1.99(2.96)	1.05(0.94-1.17)	0.40
DOLK	*NME4*	v_12_19	1.98(2.17)	2.59(3.94)	1.17(1.03-1.33)	0.01
DOLK	*AK7*	v_12_23	1.48(1.36)	1.54(2.32)	1.06(0.87-1.28)	0.58
DOLK	*WNK1*	v_12_25	1.67(1.64)	1.84(2.95)	1.11(0.96-1.29)	0.17
DOLK	*RIOK2*	v_12_27	1.41(1.09)	1.05(0.73)	0.67(0.42-1.09)	0.11
DOLK	*PTK2*	v_12_29	1.63(1.66)	1.42(1.14)	0.93(0.71-1.22)	0.59
DOLK	*TRIB1*	v_12_30	1.79(2.04)	2.07(2.70)	1.03(0.92-1.17)	0.59
DOLK	*PGK1*	v_12_31	1.39(1.40)	1.32(1.01)	1.04(0.80-1.36)	0.77
DOLK	*STK32A*	v_12_32	1.64(2.04)	1.39(1.29)	0.95(0.78-1.15)	0.59
DOLK	*SGK3*	v_12_37	1.84(1.69)	2.02(2.53)	1.07(0.89-1.29)	0.47
DOLK	*MAGI2*	v_12_38	1.89(1.69)	2.06(2.18)	1.12(0.93-1.34)	0.23
DOLK	*CERKL*	v_12_39	1.74(2.09)	1.84(4.02)	1.04(0.92-1.17)	0.56
DOLK	*CDK4*	v_12_40	1.77(2.30)	1.73(3.53)	1.00(0.88-1.15)	0.97

BMPR1A	*NEK4*	v_13_14	1.75(2.61)	1.28(1.64)	0.92(0.75-1.13)	0.41
BMPR1A	*PRKCD*	v_13_15	1.51(3.21)	1.03(0.50)	0.76(0.42-1.40)	0.38
BMPR1A	*BMPR1B*	v_13_16	1.51(1.91)	1.01(0.34)	0.74(0.47-1.17)	0.20
BMPR1A	*DYRK4*	v_13_17	1.99(3.50)	1.28(1.25)	0.90(0.75-1.09)	0.29
BMPR1A	*LIMK2*	v_13_18	1.42(1.70)	1.08(0.51)	0.84(0.58-1.22)	0.36
BMPR1A	*WNK1*	v_13_25	1.89(3.24)	1.27(1.25)	0.92(0.75-1.12)	0.40
BMPR1A	*PTK2*	v_13_29	1.60(1.96)	1.25(1.30)	0.90(0.70-1.16)	0.42
BMPR1A	*SGK3*	v_13_37	1.79(2.25)	1.53(1.81)	0.97(0.80-1.17)	0.72
BMPR1A	*MAGI2*	v_13_38	2.10(3.34)	1.65(2.12)	0.97(0.85-1.11)	0.67
NEK4	*BMPR1B*	v_14_16	1.23(1.10)	1.24(0.68)	1.08(0.81-1.43)	0.61
NEK4	*DYRK4*	v_14_17	1.08(0.52)	1.16(0.55)	1.22(0.67-2.23)	0.52
NEK4	*LIMK2*	v_14_18	1.29(1.12)	1.34(0.78)	1.12(0.85-1.47)	0.42
NEK4	*NME4*	v_14_19	1.44(1.39)	1.60(1.77)	1.16(0.95-1.42)	0.13
NEK4	*AK7*	v_14_23	1.17(0.97)	1.08(0.83)	1.00(0.66-1.50)	0.99
NEK4	*WNK1*	v_14_25	1.09(0.55)	1.17(0.58)	1.54(0.88-2.67)	0.13
NEK4	*CDC7*	v_14_26	1.30(1.35)	1.29(1.37)	1.08(0.83-1.41)	0.58
NEK4	*DGKB*	v_14_28	1.62(2.30)	1.35(1.23)	0.97(0.81-1.16)	0.72
NEK4	*PTK2*	v_14_29	1.06(0.53)	1.12(0.44)	1.15(0.65-2.02)	0.63
NEK4	*TRIB1*	v_14_30	1.25(0.96)	1.56(1.83)	1.23(0.96-1.57)	0.11
NEK4	*PRKCB*	v_14_33	1.54(2.52)	1.20(1.20)	0.97(0.78-1.22)	0.82
NEK4	*MYLK*	v_14_34	1.52(1.64)	1.58(1.33)	1.10(0.92-1.30)	0.31
NEK4	*MAP2K1*	v_14_36	1.57(2.38)	1.42(1.06)	1.02(0.88-1.18)	0.78
NEK4	*SGK3*	v_14_37	1.25(0.78)	1.39(0.95)	1.36(0.89-2.09)	0.16
NEK4	*MAGI2*	v_14_38	1.25(0.72)	1.45(0.66)	1.54(1.02-2.33)	0.04
NEK4	*CERKL*	v_14_39	1.31(1.35)	1.14(1.20)	0.93(0.68-1.29)	0.67
NEK4	*CDK4*	v_14_40	1.33(1.44)	1.34(1.52)	1.06(0.84-1.35)	0.61
PRKCD	*BMPR1B*	v_15_16	1.43(2.05)	1.16(0.76)	0.94(0.74-1.20)	0.62
PRKCD	*LIMK2*	v_15_18	1.18(0.78)	1.11(0.47)	1.02(0.60-1.73)	0.94
PRKCD	*BMP2K*	v_15_22	1.30(1.06)	1.31(1.04)	1.20(0.85-1.69)	0.31

PRKCD	*WNK1*	v_15_25	1.62(1.97)	1.73(3.21)	1.05(0.92-1.21)	0.45
PRKCD	*PTK2*	v_15_29	1.51(1.78)	1.82(3.73)	1.06(0.95-1.20)	0.29
PRKCD	*MYLK*	v_15_34	1.38(1.16)	1.17(0.67)	0.95(0.67-1.34)	0.75
PRKCD	*PI4K2B*	v_15_35	1.51(1.81)	1.66(1.57)	1.03(0.88-1.21)	0.68
PRKCD	*SGK3*	v_15_37	1.78(2.31)	2.13(4.23)	1.05(0.95-1.17)	0.31
PRKCD	*MAGI2*	v_15_38	1.94(2.97)	2.01(3.37)	1.03(0.93-1.14)	0.53
BMPR1B	*LIMK2*	v_16_18	1.38(1.60)	1.37(1.68)	0.97(0.79-1.18)	0.75
BMPR1B	*BMP2K*	v_16_22	1.53(2.15)	1.30(1.03)	0.98(0.78-1.22)	0.83
BMPR1B	*CDC7*	v_16_26	1.53(2.04)	1.58(3.20)	1.00(0.87-1.15)	0.99
BMPR1B	*PTK2*	v_16_29	1.59(3.11)	1.44(1.82)	0.96(0.83-1.11)	0.58
BMPR1B	*MYLK*	v_16_34	1.51(1.71)	1.28(1.00)	0.92(0.72-1.17)	0.50
BMPR1B	*PI4K2B*	v_16_35	2.05(2.86)	1.90(2.02)	0.96(0.84-1.11)	0.59
DYRK4	*LIMK2*	v_17_18	1.48(1.98)	1.47(1.37)	1.04(0.88-1.22)	0.65
DYRK4	*NME4*	v_17_19	1.43(1.18)	1.48(1.24)	1.14(0.88-1.47)	0.33
DYRK4	*AK7*	v_17_23	1.23(1.01)	1.50(3.01)	1.08(0.94-1.23)	0.29
DYRK4	*WNK1*	v_17_25	1.17(0.75)	1.22(1.01)	1.19(0.82-1.73)	0.36
DYRK4	*CDC7*	v_17_26	1.38(1.43)	1.19(1.01)	0.94(0.70-1.27)	0.70
DYRK4	*DGKB*	v_17_28	1.76(2.60)	1.32(1.20)	0.93(0.77-1.13)	0.48
DYRK4	*PTK2*	v_17_29	1.10(0.58)	1.50(2.61)	1.12(0.97-1.28)	0.11
DYRK4	*TRIB1*	v_17_30	1.24(0.82)	1.57(1.94)	1.20(0.96-1.50)	0.11
DYRK4	*PRKCB*	v_17_33	1.66(3.25)	1.34(1.55)	1.00(0.85-1.17)	0.98
DYRK4	*MYLK*	v_17_34	1.79(2.95)	1.45(1.14)	0.99(0.86-1.14)	0.94
DYRK4	*MAP2K1*	v_17_36	1.66(2.30)	1.51(1.54)	1.01(0.88-1.17)	0.85
DYRK4	*SGK3*	v_17_37	1.29(0.88)	1.41(0.95)	1.19(0.84-1.67)	0.33
DYRK4	*MAGI2*	v_17_38	1.33(0.95)	1.72(2.16)	1.16(0.99-1.34)	0.06
DYRK4	*CERKL*	v_17_39	1.37(1.29)	1.33(1.97)	1.02(0.81-1.29)	0.86
DYRK4	*CDK4*	v_17_40	1.38(1.51)	1.37(1.66)	1.04(0.84-1.29)	0.71
LIMK2	*BMP2K*	v_18_22	1.33(1.15)	1.26(0.92)	0.98(0.72-1.32)	0.88
LIMK2	*PI4K2B*	v_18_35	1.76(2.50)	1.73(1.78)	0.98(0.84-1.15)	0.80

LYN	*CDC7*	v_20_26	1.78(3.29)	1.15(1.12)	0.89(0.70-1.13)	0.33
LYN	*DGKB*	v_20_28	1.80(2.87)	1.21(0.96)	0.88(0.68-1.14)	0.33
LYN	*PTK2*	v_20_29	1.27(1.25)	1.41(2.12)	1.04(0.84-1.27)	0.74
LYN	*TRIB1*	v_20_30	1.35(1.36)	1.36(1.40)	0.97(0.76-1.24)	0.81
LYN	*PRKCB*	v_20_33	1.99(3.61)	1.35(1.46)	0.92(0.76-1.11)	0.39
LYN	*MYLK*	v_20_34	1.78(2.18)	1.44(1.14)	0.95(0.78-1.16)	0.61
LYN	*MAP2K1*	v_20_36	1.90(2.81)	1.41(1.27)	0.94(0.78-1.13)	0.49
LYN	*SGK3*	v_20_37	1.26(0.75)	1.33(0.81)	1.10(0.72-1.70)	0.65
LYN	*MAGI2*	v_20_38	1.36(0.95)	1.61(1.74)	1.17(0.95-1.43)	0.15
LYN	*CDK4*	v_20_40	1.78(3.31)	1.42(1.73)	0.95(0.81-1.13)	0.59
NEK9	*PGK1*	v_21_31	2.03(3.07)	1.65(1.86)	0.94(0.80-1.09)	0.38
BMP2K	*PI4K2B*	v_22_35	1.71(2.38)	1.56(1.65)	0.92(0.77-1.10)	0.37
AK7	*ULK4*	v_23_24	1.46(3.08)	1.27(0.84)	0.95(0.80-1.14)	0.60
AK7	*WNK1*	v_23_25	1.33(0.95)	1.71(1.76)	1.19(0.97-1.45)	0.10
AK7	*CDC7*	v_23_26	1.33(1.14)	1.28(1.03)	1.00(0.72-1.39)	0.98
AK7	*DGKB*	v_23_28	1.63(2.11)	1.51(1.13)	0.99(0.82-1.20)	0.92
AK7	*PTK2*	v_23_29	1.28(0.91)	1.62(1.50)	1.19(0.95-1.50)	0.13
AK7	*TRIB1*	v_23_30	1.53(1.64)	2.06(2.69)	1.11(0.96-1.28)	0.15
AK7	*PRKCB*	v_23_33	1.51(2.94)	1.21(0.61)	0.96(0.75-1.22)	0.75
AK7	*MYLK*	v_23_34	1.89(3.20)	2.26(2.88)	1.04(0.96-1.12)	0.31
AK7	*MAP2K1*	v_23_36	1.41(1.12)	1.79(1.85)	1.29(1.05-1.59)	0.02
AK7	*SGK3*	v_23_37	1.59(1.55)	2.06(2.05)	1.14(0.98-1.34)	0.09
AK7	*MAGI2*	v_23_38	1.60(1.47)	2.10(1.82)	1.21(1.01-1.44)	0.04
AK7	*CERKL*	v_23_39	1.23(0.87)	1.12(0.53)	0.82(0.50-1.35)	0.43
AK7	*CDK4*	v_23_40	1.26(0.84)	1.21(0.84)	0.97(0.64-1.45)	0.87
ULK4	*WNK1*	v_24_25	1.42(1.31)	1.61(1.60)	1.13(0.92-1.38)	0.25
ULK4	*CDC7*	v_24_26	1.29(0.78)	1.07(0.62)	0.77(0.48-1.26)	0.30
ULK4	*DGKB*	v_24_28	1.58(1.44)	1.38(1.02)	0.96(0.74-1.25)	0.78
ULK4	*PTK2*	v_24_29	1.40(1.24)	1.66(1.67)	1.16(0.95-1.42)	0.16

ULK4	*TRIB1*	v_24_30	1.41(1.67)	1.62(1.34)	1.05(0.90-1.23)	0.50
ULK4	*PRKCB*	v_24_33	1.39(1.62)	1.22(0.71)	0.97(0.75-1.26)	0.83
ULK4	*MYLK*	v_24_34	1.94(3.10)	1.86(1.87)	1.02(0.92-1.13)	0.73
ULK4	*MAP2K1*	v_24_36	1.63(1.57)	1.70(1.56)	1.09(0.90-1.31)	0.39
ULK4	*SGK3*	v_24_37	1.80(2.17)	2.14(2.37)	1.07(0.95-1.21)	0.27
ULK4	*MAGI2*	v_24_38	1.77(1.93)	2.41(3.08)	1.11(0.99-1.24)	0.06
ULK4	*CERKL*	v_24_39	1.14(0.50)	1.09(0.48)	0.92(0.48-1.76)	0.80
ULK4	*CDK4*	v_24_40	1.27(0.78)	1.23(0.85)	1.04(0.69-1.57)	0.85
WNK1	*CDC7*	v_25_26	1.30(1.37)	1.18(1.16)	0.93(0.70-1.25)	0.64
WNK1	*DGKB*	v_25_28	1.73(3.43)	1.27(1.06)	0.93(0.77-1.13)	0.48
WNK1	*PTK2*	v_25_29	1.09(0.52)	1.22(0.83)	1.21(0.72-2.04)	0.47
WNK1	*TRIB1*	v_25_30	1.33(1.31)	1.31(1.05)	0.93(0.70-1.25)	0.65
WNK1	*PRKCB*	v_25_33	1.54(1.91)	1.40(2.00)	0.99(0.81-1.20)	0.89
WNK1	*MYLK*	v_25_34	1.66(2.80)	1.49(1.63)	1.00(0.88-1.14)	0.97
WNK1	*MAP2K1*	v_25_36	1.56(2.18)	1.43(1.53)	1.01(0.86-1.18)	0.94
WNK1	*SGK3*	v_25_37	1.24(0.76)	1.24(0.64)	0.97(0.61-1.54)	0.89
WNK1	*MAGI2*	v_25_38	1.23(0.65)	1.47(0.84)	1.53(1.03-2.28)	0.04
WNK1	*CERKL*	v_25_39	1.49(2.15)	1.18(1.19)	0.89(0.69-1.16)	0.40
WNK1	*CDK4*	v_25_40	1.57(3.19)	1.60(2.93)	1.01(0.91-1.12)	0.84
CDC7	*PRKCB*	v_26_33	1.72(2.73)	1.90(3.85)	1.01(0.91-1.11)	0.92
CDC7	*CERKL*	v_26_39	1.50(2.07)	1.47(1.47)	0.97(0.82-1.14)	0.70
CDC7	*CDK4*	v_26_40	1.32(1.34)	1.55(1.58)	1.06(0.88-1.27)	0.57
DGKB	*PTK2*	v_28_29	1.58(1.99)	1.64(2.06)	1.00(0.84-1.18)	0.97
DGKB	*TRIB1*	v_28_30	1.95(3.28)	1.68(2.30)	0.95(0.84-1.07)	0.42
DGKB	*MYLK*	v_28_34	1.88(2.46)	1.98(2.95)	1.03(0.91-1.17)	0.67
DGKB	*MAP2K1*	v_28_36	1.41(1.11)	1.37(0.90)	1.01(0.74-1.37)	0.97
DGKB	*SGK3*	v_28_37	1.70(1.96)	2.27(4.51)	1.08(0.98-1.19)	0.14
DGKB	*MAGI2*	v_28_38	1.78(3.17)	2.12(2.75)	1.05(0.96-1.15)	0.30
PTK2	*TRIB1*	v_29_30	1.27(1.09)	1.41(1.42)	1.10(0.85-1.43)	0.47

PTK2	*PRKCB*	v_29_33	1.46(1.64)	1.17(0.92)	0.93(0.69-1.24)	0.61
PTK2	*MYLK*	v_29_34	1.61(1.98)	2.03(4.63)	1.11(1.01-1.22)	0.03
PTK2	*MAP2K1*	v_29_36	1.62(2.00)	1.83(3.67)	1.10(0.98-1.25)	0.12
PTK2	*SGK3*	v_29_37	1.25(0.71)	1.30(0.82)	1.18(0.75-1.86)	0.48
PTK2	*MAGI2*	v_29_38	1.29(0.73)	1.47(0.95)	1.42(0.99-2.02)	0.05
PTK2	*CERKL*	v_29_39	1.43(1.58)	1.26(1.60)	0.96(0.75-1.24)	0.77
PTK2	*CDK4*	v_29_40	1.40(1.60)	1.30(1.31)	1.00(0.79-1.27)	1.00
TRIB1	*SGK3*	v_30_37	1.33(0.89)	1.36(1.09)	1.07(0.77-1.50)	0.69
TRIB1	*MAGI2*	v_30_38	1.53(1.77)	1.59(1.23)	1.04(0.88-1.24)	0.61
PGK1	*STK32A*	v_31_32	1.58(1.92)	1.33(1.12)	0.92(0.73-1.16)	0.48
PGK1	*MAP2K1*	v_31_36	2.15(3.08)	1.88(1.85)	1.01(0.90-1.13)	0.90
PGK1	*SGK3*	v_31_37	2.09(2.96)	1.86(1.96)	0.98(0.84-1.14)	0.77
PGK1	*MAGI2*	v_31_38	1.97(2.93)	2.07(2.45)	1.03(0.92-1.14)	0.64
PGK1	*CERKL*	v_31_39	1.94(3.04)	1.38(1.57)	0.92(0.74-1.13)	0.41
STK32A	*PRKCB*	v_32_33	2.10(3.25)	1.95(2.58)	1.01(0.9-1.120)	0.93
STK32A	*MAGI2*	v_32_38	2.20(2.11)	2.98(4.25)	1.10(1.00-1.22)	0.05
STK32A	*CERKL*	v_32_39	1.96(2.45)	1.98(3.10)	1.02(0.90-1.16)	0.77
STK32A	*CDK4*	v_32_40	2.14(3.18)	1.96(2.50)	0.99(0.88-1.12)	0.87
PRKCB	*MYLK*	v_33_34	1.68(2.17)	1.87(2.20)	1.07(0.94-1.21)	0.30
PRKCB	*MAP2K1*	v_33_36	1.35(0.93)	1.56(1.64)	1.34(1.02-1.76)	0.03
PRKCB	*SGK3*	v_33_37	1.49(1.25)	1.76(1.69)	1.17(0.93-1.48)	0.18
PRKCB	*MAGI2*	v_33_38	1.49(1.46)	1.91(1.84)	1.17(0.99-1.37)	0.07
PRKCB	*CERKL*	v_33_39	1.32(1.65)	1.06(0.61)	0.81(0.53-1.23)	0.32
PRKCB	*CDK4*	v_33_40	1.31(1.23)	1.12(0.73)	0.84(0.57-1.23)	0.36
MYLK	*PI4K2B*	v_34_35	1.59(1.96)	1.53(1.54)	0.92(0.75-1.13)	0.42
MAP2K1	*SGK3*	v_36_37	1.56(1.73)	2.39(4.98)	1.07(0.99-1.16)	0.10
MAP2K1	*CDK4*	v_36_40	1.51(2.10)	1.28(1.22)	0.91(0.72-1.14)	0.41
SGK3	*MAGI2*	v_37_38	1.44(1.56)	1.53(1.31)	1.07(0.89-1.28)	0.49
CERKL	*CDK4*	v_39_40	1.15(0.56)	1.17(0.70)	1.12(0.65-1.93)	0.68

Table S7. Association analysis of gene ratios with survival

Gene N (numerator)	Gene D (denominator)	Variables	Alive mean(SD)	Dead mean(SD)	HR (95%CI)	P value
			**Training set**			
MAP4K1	*STK39*	v_1_3	2.30(2.35)	2.47(2.98)	1.03(0.89-1.20)	0.65
MAP4K1	*DOLK*	v_1_12	1.09(0.70)	1.61(2.26)	1.07(0.90-1.28)	0.41
MAP4K1	*NEK4*	v_1_14	1.86(1.73)	2.63(3.09)	1.15(0.99-1.34)	0.06
MAP4K1	*RIOK2*	v_1_27	1.55(1.48)	1.63(1.57)	1.02(0.80-1.30)	0.88
MAP4K1	*PTK2*	v_1_29	2.06(2.73)	2.64(2.97)	1.05(0.94-1.17)	0.39
CDKL4	*STK39*	v_2_3	1.15(0.42)	1.26(0.59)	1.65(0.74-3.70)	0.22
CDKL4	*PRKD3*	v_2_4	1.96(1.49)	2.12(1.66)	1.11(0.87-1.42)	0.40
CDKL4	*AGK*	v_2_5	1.09(0.64)	1.56(0.83)	2.18(1.32-3.60)	0.002
CDKL4	*PIK3CA*	v_2_6	3.33(4.21)	2.82(3.45)	0.98(0.88-1.08)	0.65
CDKL4	*PKN3*	v_2_7	2.65(4.04)	2.73(3.44)	1.02(0.93-1.12)	0.70
CDKL4	*MST1R*	v_2_9	1.39(2.49)	1.67(2.82)	1.03(0.90-1.17)	0.69
CDKL4	*NEK8*	v_2_10	2.26(4.74)	2.43(2.69)	1.01(0.93-1.09)	0.87
CDKL4	*SPHK2*	v_2_11	1.49(1.27)	2.41(3.46)	1.13(1.01-1.26)	0.03
CDKL4	*BMPR1A*	v_2_13	1.41(1.34)	2.34(4.32)	1.09(0.99-1.19)	0.07
CDKL4	*NEK4*	v_2_14	1.02(0.33)	1.62(2.59)	1.15(1.00-1.33)	0.05
CDKL4	*BMPR1B*	v_2_16	1.47(1.88)	2.15(4.59)	1.07(0.97-1.16)	0.16
CDKL4	*DYRK4*	v_2_17	1.06(0.46)	1.23(0.84)	1.37(0.83-2.24)	0.22
CDKL4	*LIMK2*	v_2_18	1.56(1.92)	2.46(5.48)	1.06(0.98-1.14)	0.13
CDKL4	*AK7*	v_2_23	1.04(1.03)	2.16(3.83)	1.11(1.01-1.22)	0.03
CDKL4	*WNK1*	v_2_25	0.98(0.35)	1.45(0.80)	4.94(2.62-9.29)	<0.001
CDKL4	*CDC7*	v_2_26	1.11(1.04)	2.13(3.04)	1.17(1.03-1.33)	0.02
CDKL4	*PTK2*	v_2_29	0.99(0.44)	1.57(1.88)	1.26(1.05-1.50)	0.01
CDKL4	*TRIB1*	v_2_30	1.20(0.90)	1.60(1.03)	1.27(0.91-1.79)	0.16
CDKL4	*MYLK*	v_2_34	1.96(2.74)	2.72(4.74)	1.06(0.98-1.15)	0.14

CDKL4	*MAP2K1*	v_2_36	1.50(2.53)	2.93(4.75)	1.07(0.99-1.16)	0.07
CDKL4	*SGK3*	v_2_37	1.32(0.86)	1.76(1.12)	1.67(1.11-2.51)	0.01
CDKL4	*MAGI2*	v_2_38	1.17(0.61)	1.94(1.27)	1.76(1.30-2.38)	<0.001
CDKL4	*CERKL*	v_2_39	1.08(0.93)	2.22(2.95)	1.19(1.04-1.35)	0.01
STK39	*PRKD3*	v_3_4	1.70(1.06)	1.79(1.23)	1.09(0.78-1.51)	0.63
STK39	*AGK*	v_3_5	1.03(0.55)	1.41(0.93)	2.43(1.41-4.19)	0.001
STK39	*PKN3*	v_3_7	2.46(3.46)	2.15(2.89)	0.98(0.87-1.11)	0.75
STK39	*MST1R*	v_3_9	1.38(2.21)	1.34(1.41)	0.98(0.80-1.22)	0.88
STK39	*NEK8*	v_3_10	2.00(3.32)	2.46(3.15)	1.03(0.92-1.14)	0.61
STK39	*SPHK2*	v_3_11	1.73(2.89)	1.80(1.50)	1.02(0.89-1.17)	0.77
STK39	*BMPR1A*	v_3_13	1.82(3.77)	1.76(1.68)	1.00(0.89-1.13)	0.94
STK39	*NEK4*	v_3_14	1.04(0.65)	1.26(1.03)	1.21(0.82-1.77)	0.34
STK39	*BMPR1B*	v_3_16	1.61(2.52)	1.58(1.77)	1.02(0.86-1.20)	0.82
STK39	*DYRK4*	v_3_17	1.00(0.37)	1.02(0.47)	1.08(0.40-2.91)	0.88
STK39	*LIMK2*	v_3_18	1.75(2.76)	1.77(2.15)	1.03(0.89-1.19)	0.72
STK39	*AK7*	v_3_23	1.12(1.72)	1.69(1.75)	1.13(0.94-1.36)	0.19
STK39	*WNK1*	v_3_25	0.97(0.48)	1.38(1.01)	2.32(1.45-3.71)	<0.001
STK39	*CDC7*	v_3_26	1.33(2.26)	1.98(2.46)	1.09(0.95-1.25)	0.23
STK39	*PTK2*	v_3_29	0.96(0.51)	1.30(1.01)	1.50(0.98-2.29)	0.06
STK39	*TRIB1*	v_3_30	1.19(1.03)	1.48(1.13)	1.14(0.82-1.57)	0.44
STK39	*MYLK*	v_3_34	2.23(3.99)	2.22(2.37)	1.02(0.93-1.13)	0.67
STK39	*MAP2K1*	v_3_36	1.76(3.87)	2.48(3.16)	1.03(0.95-1.13)	0.44
STK39	*SGK3*	v_3_37	1.18(0.64)	1.47(0.90)	1.59(0.98-2.56)	0.06
STK39	*MAGI2*	v_3_38	1.10(0.57)	1.69(1.35)	1.45(1.10-1.90)	0.01
STK39	*CERKL*	v_3_39	1.24(1.65)	2.04(2.27)	1.16(0.97-1.39)	0.10
STK39	*CDK4*	v_3_40	1.41(2.65)	2.17(3.34)	1.06(0.95-1.19)	0.31
PRKD3	*AGK*	v_4_5	1.72(3.40)	1.95(2.42)	1.00(0.90-1.12)	0.97
PRKD3	*WNK1*	v_4_25	1.59(2.33)	2.27(4.47)	1.04(0.94-1.15)	0.43
PRKD3	*SGK3*	v_4_37	1.48(2.38)	1.49(1.52)	0.98(0.82-1.16)	0.80

AGK	*DYRK4*	v_5_17	1.65(2.44)	0.95(0.67)	0.58(0.28-1.21)	0.15
AGK	*LIMK2*	v_5_18	2.01(2.75)	1.72(3.43)	0.97(0.83-1.14)	0.74
AGK	*PTK2*	v_5_29	1.49(2.15)	1.38(1.71)	0.94(0.76-1.16)	0.57
AGK	*MYLK*	v_5_34	2.46(3.37)	1.86(2.91)	0.96(0.83-1.11)	0.58
AGK	*SGK3*	v_5_37	1.25(0.65)	1.25(0.72)	1.10(0.61-1.99)	0.76
AGK	*MAGI2*	v_5_38	1.41(1.12)	1.67(1.50)	1.07(0.83-1.39)	0.60
HK2	*SPHK2*	v_8_11	1.53(1.07)	1.64(1.39)	1.11(0.79-1.54)	0.55
HK2	*BMPR1A*	v_8_13	1.31(0.86)	1.33(0.80)	0.98(0.62-1.56)	0.94
HK2	*NEK4*	v_8_14	1.79(2.41)	2.33(3.80)	1.03(0.92-1.16)	0.57
HK2	*PRKCD*	v_8_15	1.22(0.66)	1.13(0.69)	0.83(0.46-1.49)	0.54
HK2	*BMPR1B*	v_8_16	1.47(1.12)	1.44(1.18)	0.99(0.70-1.38)	0.93
HK2	*DYRK4*	v_8_17	1.82(2.22)	1.99(2.79)	1.01(0.86-1.18)	0.92
HK2	*LIMK2*	v_8_18	1.25(0.72)	1.13(0.63)	0.82(0.46-1.45)	0.50
HK2	*BMP2K*	v_8_22	1.34(0.90)	1.29(0.71)	1.01(0.66-1.54)	0.97
HK2	*WNK1*	v_8_25	1.74(2.30)	2.28(3.26)	1.05(0.93-1.18)	0.46
HK2	*CDC7*	v_8_26	1.57(1.76)	2.81(6.21)	1.03(0.96-1.11)	0.37
HK2	*PTK2*	v_8_29	1.62(1.95)	2.22(3.15)	1.06(0.93-1.21)	0.41
HK2	*TRIB1*	v_8_30	1.83(2.49)	2.78(4.60)	1.04(0.95-1.14)	0.39
HK2	*MYLK*	v_8_34	1.59(1.53)	1.27(1.07)	0.93(0.69-1.27)	0.66
HK2	*PI4K2B*	v_8_35	1.96(1.62)	1.86(1.91)	1.00(0.80-1.26)	0.98
HK2	*SGK3*	v_8_37	2.17(2.83)	3.01(4.33)	1.05(0.95-1.15)	0.34
HK2	*MAGI2*	v_8_38	2.13(3.07)	3.36(5.60)	1.04(0.97-1.12)	0.27
MST1R	*SPHK2*	v_9_11	1.78(1.62)	2.54(2.01)	1.26(1.04-1.52)	0.02
MST1R	*BMPR1A*	v_9_13	1.65(1.64)	2.27(1.99)	1.20(0.99-1.45)	0.06
MST1R	*NEK4*	v_9_14	1.41(0.88)	1.84(1.65)	1.20(0.96-1.51)	0.12
MST1R	*BMPR1B*	v_9_16	2.00(3.38)	1.96(1.70)	1.02(0.91-1.14)	0.75
MST1R	*DYRK4*	v_9_17	1.50(1.08)	1.73(1.59)	1.13(0.87-1.49)	0.36
MST1R	*LIMK2*	v_9_18	1.95(3.20)	2.06(1.75)	1.03(0.92-1.15)	0.62
MST1R	*NME4*	v_9_19	1.76(1.78)	2.27(2.66)	1.16(0.99-1.37)	0.07

MST1R	*AK7*	v_9_23	1.04(0.54)	1.34(0.45)	2.30(1.15-4.58)	0.02
MST1R	*ULK4*	v_9_24	1.13(0.74)	1.57(1.06)	1.52(1.05-2.18)	0.02
MST1R	*WNK1*	v_9_25	1.41(0.91)	2.00(1.49)	1.50(1.13-1.98)	0.005
MST1R	*CDC7*	v_9_26	1.40(1.08)	1.59(1.23)	1.27(0.90-1.81)	0.18
MST1R	*DGKB*	v_9_28	1.44(0.98)	1.94(3.23)	1.11(0.98-1.26)	0.10
MST1R	*PTK2*	v_9_29	1.38(0.98)	1.89(1.56)	1.28(1.00-1.66)	0.05
MST1R	*TRIB1*	v_9_30	1.59(1.36)	1.87(1.39)	1.17(0.90-1.51)	0.25
MST1R	*MYLK*	v_9_34	2.59(4.81)	2.74(2.89)	1.02(0.95-1.10)	0.50
MST1R	*MAP2K1*	v_9_36	1.56(1.30)	2.29(2.05)	1.35(1.10-1.67)	0.01
MST1R	*SGK3*	v_9_37	1.93(1.55)	2.67(2.61)	1.23(1.03-1.46)	0.02
MST1R	*MAGI2*	v_9_38	1.78(1.41)	2.93(2.84)	1.18(1.03-1.36)	0.02
MST1R	*CERKL*	v_9_39	1.18(0.98)	1.51(1.07)	1.22(0.93-1.61)	0.15
MST1R	*CDK4*	v_9_40	1.34(1.33)	1.59(0.96)	1.13(0.87-1.46)	0.37
NEK8	*NEK4*	v_10_14	2.42(3.04)	2.37(3.80)	0.98(0.87-1.11)	0.75
NEK8	*BMPR1B*	v_10_16	2.41(3.74)	2.28(4.31)	1.00(0.90-1.10)	0.93
NEK8	*LIMK2*	v_10_18	2.55(4.14)	1.83(2.84)	0.95(0.82-1.09)	0.45
NEK8	*NME4*	v_10_19	1.81(3.41)	1.27(0.97)	0.91(0.73-1.15)	0.43
NEK8	*AK7*	v_10_23	2.10(3.16)	1.48(1.47)	0.90(0.75-1.08)	0.27
NEK8	*WNK1*	v_10_25	2.16(2.97)	2.52(4.42)	1.01(0.91-1.12)	0.80
NEK8	*CDC7*	v_10_26	1.59(2.00)	1.32(1.23)	0.92(0.72-1.16)	0.46
NEK8	*PTK2*	v_10_29	2.27(3.17)	2.37(3.95)	1.00(0.89-1.12)	0.94
NEK8	*PRKCB*	v_10_33	2.32(3.99)	1.64(1.48)	0.93(0.79-1.09)	0.35
NEK8	*MYLK*	v_10_34	2.49(3.05)	2.53(4.64)	1.02(0.91-1.13)	0.75
NEK8	*MAP2K1*	v_10_36	2.02(3.01)	1.42(0.85)	0.87(0.69-1.11)	0.26
NEK8	*CERKL*	v_10_39	2.67(4.12)	1.78(2.36)	0.92(0.81-1.06)	0.27
SPHK2	*BMPR1A*	v_11_13	1.14(0.70)	1.18(0.66)	1.00(0.57-1.75)	0.99
SPHK2	*NEK4*	v_11_14	1.93(2.93)	1.95(2.90)	0.99(0.87-1.14)	0.94
SPHK2	*BMPR1B*	v_11_16	1.84(2.83)	1.36(1.44)	0.92(0.76-1.13)	0.43
SPHK2	*DYRK4*	v_11_17	2.34(3.64)	2.08(3.36)	0.97(0.86-1.10)	0.66

SPHK2	*LIMK2*	v_11_18	1.49(1.90)	1.17(0.97)	0.89(0.64-1.23)	0.47
SPHK2	*LYN*	v_11_20	1.93(3.55)	2.09(4.04)	1.02(0.92-1.13)	0.72
SPHK2	*WNK1*	v_11_25	2.06(3.24)	2.26(3.96)	1.01(0.91-1.12)	0.85
SPHK2	*PTK2*	v_11_29	1.72(2.22)	2.03(3.52)	1.02(0.89-1.16)	0.80
SPHK2	*SGK3*	v_11_37	1.92(2.56)	2.43(3.84)	1.03(0.93-1.15)	0.57
DOLK	*BMPR1A*	v_12_13	1.92(1.74)	2.96(3.72)	1.21(1.05-1.38)	0.01
DOLK	*NEK4*	v_12_14	1.80(1.25)	2.14(1.86)	1.22(0.93-1.61)	0.15
DOLK	*BMPR1B*	v_12_16	2.21(3.40)	2.66(3.51)	1.05(0.97-1.15)	0.23
DOLK	*DYRK4*	v_12_17	1.81(1.24)	2.07(2.09)	1.15(0.89-1.49)	0.27
DOLK	*LIMK2*	v_12_18	2.38(3.34)	2.87(3.66)	1.05(0.96-1.15)	0.27
DOLK	*NME4*	v_12_19	2.40(2.57)	3.79(4.67)	1.14(1.03-1.26)	0.01
DOLK	*AK7*	v_12_23	1.54(1.51)	2.25(2.88)	1.08(0.95-1.23)	0.23
DOLK	*WNK1*	v_12_25	1.77(1.34)	2.81(3.47)	1.25(1.10-1.42)	<0.001
DOLK	*RIOK2*	v_12_27	1.48(1.21)	1.39(1.44)	0.99(0.73-1.36)	0.96
DOLK	*PTK2*	v_12_29	1.81(1.56)	2.25(2.50)	1.16(0.95-1.42)	0.15
DOLK	*TRIB1*	v_12_30	2.21(2.66)	2.75(3.08)	1.05(0.94-1.17)	0.43
DOLK	*PGK1*	v_12_31	1.38(1.42)	1.90(2.06)	1.13(0.95-1.33)	0.17
DOLK	*STK32A*	v_12_32	1.23(0.88)	1.58(1.39)	1.30(0.95-1.78)	0.10
DOLK	*SGK3*	v_12_37	2.43(2.23)	2.77(2.74)	1.07(0.91-1.26)	0.40
DOLK	*MAGI2*	v_12_38	2.15(1.69)	3.30(2.89)	1.20(1.04-1.40)	0.02
DOLK	*CERKL*	v_12_39	1.75(1.93)	3.14(5.11)	1.06(0.98-1.14)	0.14
DOLK	*CDK4*	v_12_40	1.93(2.54)	2.75(4.72)	1.03(0.95-1.12)	0.48
BMPR1A	*NEK4*	v_13_14	2.15(3.37)	1.99(2.91)	1.00(0.87-1.13)	0.94
BMPR1A	*PRKCD*	v_13_15	2.03(4.67)	1.03(0.65)	0.81(0.48-1.36)	0.42
BMPR1A	*BMPR1B*	v_13_16	1.88(2.57)	1.28(1.35)	0.89(0.70-1.14)	0.37
BMPR1A	*DYRK4*	v_13_17	2.71(4.80)	1.82(2.45)	0.95(0.84-1.08)	0.42
BMPR1A	*LIMK2*	v_13_18	1.55(1.95)	1.27(1.84)	0.95(0.74-1.23)	0.71
BMPR1A	*WNK1*	v_13_25	2.38(4.22)	2.08(3.01)	0.99(0.89-1.10)	0.83
BMPR1A	*PTK2*	v_13_29	1.80(2.21)	1.91(2.65)	1.02(0.87-1.20)	0.81

BMPR1A	*SGK3*	v_13_37	2.08(2.48)	2.44(3.25)	1.04(0.92-1.19)	0.51
BMPR1A	*MAGI2*	v_13_38	2.44(3.87)	2.85(4.40)	1.03(0.94-1.12)	0.57
NEK4	*BMPR1B*	v_14_16	1.41(1.47)	1.27(0.92)	1.00(0.74-1.34)	0.99
NEK4	*DYRK4*	v_14_17	1.11(0.54)	1.07(0.55)	0.83(0.41-1.68)	0.60
NEK4	*LIMK2*	v_14_18	1.46(1.41)	1.40(1.06)	1.03(0.77-1.38)	0.85
NEK4	*NME4*	v_14_19	1.45(1.58)	1.70(1.81)	1.09(0.89-1.32)	0.42
NEK4	*AK7*	v_14_23	1.00(0.84)	1.53(1.61)	1.22(0.94-1.57)	0.13
NEK4	*WNK1*	v_14_25	1.05(0.53)	1.30(0.78)	2.13(1.18-3.86)	0.01
NEK4	*CDC7*	v_14_26	1.21(1.17)	1.80(2.37)	1.14(0.95-1.38)	0.17
NEK4	*DGKB*	v_14_28	1.61(2.59)	1.90(2.76)	1.03(0.91-1.17)	0.65
NEK4	*PTK2*	v_14_29	1.00(0.40)	1.10(0.51)	1.60(0.70-3.66)	0.27
NEK4	*TRIB1*	v_14_30	1.28(1.00)	1.47(1.25)	1.06(0.78-1.44)	0.71
NEK4	*PRKCB*	v_14_33	1.16(1.32)	2.64(4.51)	1.10(1.01-1.20)	0.03
NEK4	*MYLK*	v_14_34	1.88(2.18)	1.92(1.70)	1.06(0.90-1.25)	0.49
NEK4	*MAP2K1*	v_14_36	1.53(2.50)	2.32(3.20)	1.06(0.96-1.17)	0.29
NEK4	*SGK3*	v_14_37	1.35(0.85)	1.59(1.13)	1.34(0.90-2.00)	0.15
NEK4	*MAGI2*	v_14_38	1.19(0.63)	1.60(0.95)	1.62(1.06-2.47)	0.03
NEK4	*CERKL*	v_14_39	1.15(1.12)	1.89(2.38)	1.15(0.96-1.37)	0.13
NEK4	*CDK4*	v_14_40	1.14(1.22)	1.96(2.64)	1.13(0.96-1.32)	0.15
PRKCD	*BMPR1B*	v_15_16	1.71(2.89)	1.52(1.30)	0.97(0.82-1.15)	0.72
PRKCD	*LIMK2*	v_15_18	1.16(0.60)	1.26(1.11)	1.19(0.76-1.87)	0.45
PRKCD	*BMP2K*	v_15_22	1.27(1.00)	1.81(1.71)	1.35(1.03-1.78)	0.03
PRKCD	*WNK1*	v_15_25	1.92(2.52)	2.59(3.95)	1.03(0.93-1.15)	0.51
PRKCD	*PTK2*	v_15_29	1.79(2.32)	2.49(4.46)	1.03(0.93-1.13)	0.57
PRKCD	*MYLK*	v_15_34	1.53(1.41)	1.52(1.24)	1.06(0.82-1.38)	0.64
PRKCD	*PI4K2B*	v_15_35	1.89(2.36)	1.99(2.11)	1.03(0.89-1.19)	0.71
PRKCD	*SGK3*	v_15_37	2.23(3.05)	3.14(4.98)	1.03(0.95-1.11)	0.48
PRKCD	*MAGI2*	v_15_38	2.29(3.77)	3.19(4.56)	1.04(0.96-1.12)	0.38
BMPR1B	*LIMK2*	v_16_18	1.68(2.25)	1.48(1.95)	0.93(0.76-1.13)	0.44

BMPR1B	*BMP2K*	v_16_22	1.51(1.75)	2.23(3.50)	1.06(0.95-1.18)	0.29
BMPR1B	*CDC7*	v_16_26	1.76(2.80)	2.13(3.74)	1.01(0.90-1.12)	0.93
BMPR1B	*PTK2*	v_16_29	2.10(4.51)	1.68(2.10)	0.95(0.83-1.08)	0.45
BMPR1B	*MYLK*	v_16_34	1.86(2.35)	1.56(1.31)	0.95(0.78-1.16)	0.63
BMPR1B	*PI4K2B*	v_16_35	2.85(3.66)	2.64(2.99)	0.98(0.87-1.10)	0.71
DYRK4	*LIMK2*	v_17_18	1.83(2.72)	1.82(1.86)	1.01(0.88-1.17)	0.87
DYRK4	*NME4*	v_17_19	1.48(1.42)	1.83(1.57)	1.11(0.89-1.39)	0.34
DYRK4	*AK7*	v_17_23	1.15(1.14)	2.06(3.57)	1.06(0.95-1.17)	0.31
DYRK4	*WNK1*	v_17_25	1.09(0.66)	1.58(1.42)	1.32(1.01-1.73)	0.04
DYRK4	*CDC7*	v_17_26	1.32(1.66)	1.76(1.77)	1.12(0.91-1.37)	0.28
DYRK4	*DGKB*	v_17_28	1.77(2.80)	2.11(3.30)	1.03(0.92-1.15)	0.62
DYRK4	*PTK2*	v_17_29	1.03(0.46)	1.76(3.02)	1.06(0.94-1.20)	0.31
DYRK4	*TRIB1*	v_17_30	1.21(0.94)	1.67(1.58)	1.12(0.89-1.41)	0.32
DYRK4	*PRKCB*	v_17_33	1.23(1.36)	3.12(6.00)	1.07(1.01-1.14)	0.02
DYRK4	*MYLK*	v_17_34	2.31(4.12)	2.02(1.79)	1.00(0.90-1.12)	0.96
DYRK4	*MAP2K1*	v_17_36	1.69(3.00)	2.32(2.20)	1.04(0.94-1.16)	0.44
DYRK4	*SGK3*	v_17_37	1.30(0.89)	1.69(1.05)	1.47(1.01-2.15)	0.05
DYRK4	*MAGI2*	v_17_38	1.20(0.70)	2.21(2.65)	1.12(0.98-1.27)	0.08
DYRK4	*CERKL*	v_17_39	1.26(1.24)	2.12(2.74)	1.11(0.96-1.28)	0.16
DYRK4	*CDK4*	v_17_40	1.29(1.87)	1.90(2.22)	1.09(0.93-1.28)	0.28
LIMK2	*BMP2K*	v_18_22	1.33(1.21)	1.70(1.52)	1.16(0.92-1.47)	0.21
LIMK2	*PI4K2B*	v_18_35	2.32(3.53)	1.88(1.81)	0.98(0.84-1.13)	0.75
LYN	*CDC7*	v_20_26	2.32(4.67)	1.65(1.86)	0.95(0.83-1.08)	0.40
LYN	*DGKB*	v_20_28	1.89(3.30)	1.94(3.35)	1.00(0.89-1.12)	0.94
LYN	*PTK2*	v_20_29	1.47(1.71)	1.47(2.48)	0.94(0.77-1.16)	0.57
LYN	*TRIB1*	v_20_30	1.53(1.81)	1.51(1.55)	0.95(0.74-1.21)	0.66
LYN	*PRKCB*	v_20_33	1.94(2.84)	2.95(5.94)	1.04(0.96-1.12)	0.38
LYN	*MYLK*	v_20_34	2.34(2.93)	1.67(1.51)	0.93(0.78-1.11)	0.42
LYN	*MAP2K1*	v_20_36	2.19(3.72)	2.02(2.27)	0.97(0.85-1.10)	0.60

LYN	*SGK3*	v_20_37	1.31(0.74)	1.42(0.84)	1.13(0.68-1.88)	0.64
LYN	*MAGI2*	v_20_38	1.35(0.80)	1.86(2.22)	1.08(0.89-1.30)	0.43
LYN	*CDK4*	v_20_40	2.15(4.71)	1.87(2.29)	0.97(0.87-1.10)	0.65
NEK9	*PGK1*	v_21_31	2.51(3.52)	2.51(3.90)	0.98(0.88-1.09)	0.69
BMP2K	*PI4K2B*	v_22_35	2.42(3.29)	1.50(1.84)	0.88(0.73-1.06)	0.18
AK7	*ULK4*	v_23_24	1.86(4.49)	1.27(0.80)	0.94(0.76-1.16)	0.55
AK7	*WNK1*	v_23_25	1.79(1.74)	1.63(1.19)	0.97(0.77-1.22)	0.78
AK7	*CDC7*	v_23_26	1.51(1.56)	1.34(1.06)	0.94(0.71-1.26)	0.69
AK7	*DGKB*	v_23_28	1.86(2.06)	1.63(2.87)	0.96(0.79-1.17)	0.71
AK7	*PTK2*	v_23_29	1.62(1.47)	1.53(1.24)	0.96(0.74-1.25)	0.75
AK7	*TRIB1*	v_23_30	2.07(2.29)	1.60(1.32)	0.91(0.72-1.13)	0.39
AK7	*PRKCB*	v_23_33	1.44(1.78)	2.11(5.16)	1.04(0.96-1.14)	0.34
AK7	*MYLK*	v_23_34	2.98(4.78)	2.33(2.51)	0.98(0.88-1.09)	0.72
AK7	*MAP2K1*	v_23_36	1.77(1.70)	1.85(1.73)	1.04(0.83-1.29)	0.74
AK7	*SGK3*	v_23_37	2.22(2.16)	2.10(2.02)	1.00(0.83-1.20)	0.99
AK7	*MAGI2*	v_23_38	2.00(1.89)	2.37(2.14)	1.06(0.90-1.26)	0.47
AK7	*CERKL*	v_23_39	1.29(0.93)	1.27(1.01)	0.99(0.65-1.50)	0.96
AK7	*CDK4*	v_23_40	1.30(1.03)	1.23(0.72)	0.92(0.61-1.39)	0.70
ULK4	*WNK1*	v_24_25	1.89(1.89)	1.76(1.65)	0.99(0.80-1.21)	0.89
ULK4	*CDC7*	v_24_26	1.50(1.04)	1.18(0.73)	0.80(0.53-1.21)	0.29
ULK4	*DGKB*	v_24_28	1.89(1.67)	1.39(1.27)	0.86(0.64-1.14)	0.29
ULK4	*PTK2*	v_24_29	1.74(1.63)	1.79(1.93)	1.02(0.83-1.26)	0.83
ULK4	*TRIB1*	v_24_30	1.84(2.35)	1.57(1.36)	0.95(0.77-1.19)	0.67
ULK4	*PRKCB*	v_24_33	1.44(1.87)	1.53(1.79)	1.01(0.85-1.22)	0.87
ULK4	*MYLK*	v_24_34	2.90(4.39)	1.99(1.92)	0.96(0.84-1.10)	0.59
ULK4	*MAP2K1*	v_24_36	1.98(1.99)	2.01(1.92)	1.03(0.85-1.23)	0.79
ULK4	*SGK3*	v_24_37	2.48(2.83)	2.62(2.85)	1.02(0.91-1.16)	0.70
ULK4	*MAGI2*	v_24_38	2.30(2.52)	2.90(3.62)	1.04(0.93-1.17)	0.45
ULK4	*CERKL*	v_24_39	1.15(0.52)	1.10(0.45)	0.90(0.42-1.92)	0.79

ULK4	*CDK4*	v_24_40	1.26(0.84)	1.38(1.03)	1.17(0.78-1.76)	0.46
WNK1	*CDC7*	v_25_26	1.45(1.82)	1.42(1.65)	0.96(0.77-1.20)	0.73
WNK1	*DGKB*	v_25_28	2.09(4.87)	1.38(1.45)	0.93(0.77-1.13)	0.48
WNK1	*PTK2*	v_25_29	1.15(0.62)	1.11(0.78)	0.83(0.45-1.54)	0.55
WNK1	*TRIB1*	v_25_30	1.49(1.48)	1.33(1.56)	0.90(0.67-1.19)	0.45
WNK1	*PRKCB*	v_25_33	1.56(2.15)	2.19(3.14)	1.06(0.92-1.20)	0.42
WNK1	*MYLK*	v_25_34	2.27(3.99)	1.62(1.90)	0.96(0.82-1.12)	0.60
WNK1	*MAP2K1*	v_25_36	1.67(2.81)	2.04(2.45)	1.03(0.91-1.15)	0.68
WNK1	*SGK3*	v_25_37	1.36(0.84)	1.33(0.82)	0.97(0.60-1.57)	0.90
WNK1	*MAGI2*	v_25_38	1.20(0.59)	1.56(1.01)	1.41(0.93-2.14)	0.11
WNK1	*CERKL*	v_25_39	1.74(2.93)	1.61(1.96)	0.96(0.82-1.13)	0.66
WNK1	*CDK4*	v_25_40	1.82(4.54)	2.13(3.62)	1.01(0.93-1.10)	0.84
CDC7	*PRKCB*	v_26_33	2.47(4.67)	1.91(2.82)	0.96(0.86-1.07)	0.45
CDC7	*CERKL*	v_26_39	2.01(3.04)	1.45(1.34)	0.89(0.73-1.09)	0.28
CDC7	*CDK4*	v_26_40	1.50(1.87)	1.58(1.69)	0.98(0.80-1.19)	0.83
DGKB	*PTK2*	v_28_29	1.59(1.82)	2.13(3.02)	1.07(0.93-1.24)	0.35
DGKB	*TRIB1*	v_28_30	2.43(4.18)	1.94(2.51)	0.97(0.85-1.09)	0.58
DGKB	*MYLK*	v_28_34	2.28(2.79)	2.51(3.78)	1.05(0.93-1.18)	0.43
DGKB	*MAP2K1*	v_28_36	1.47(1.19)	1.69(0.93)	1.13(0.82-1.55)	0.45
DGKB	*SGK3*	v_28_37	1.81(1.93)	3.11(5.45)	1.08(1.00-1.17)	0.05
DGKB	*MAGI2*	v_28_38	1.63(2.02)	3.39(5.82)	1.06(1.00-1.13)	0.06
PTK2	*TRIB1*	v_29_30	1.34(1.31)	1.52(1.41)	1.07(0.83-1.39)	0.59
PTK2	*PRKCB*	v_29_33	1.31(1.47)	2.01(2.28)	1.13(0.96-1.33)	0.16
PTK2	*MYLK*	v_29_34	2.09(2.71)	2.59(5.40)	1.07(0.98-1.18)	0.14
PTK2	*MAP2K1*	v_29_36	1.63(2.15)	2.82(4.75)	1.10(1.00-1.21)	0.04
PTK2	*SGK3*	v_29_37	1.32(0.77)	1.56(1.04)	1.34(0.89-2.03)	0.16
PTK2	*MAGI2*	v_29_38	1.27(0.69)	1.73(1.22)	1.42(1.04-1.96)	0.03
PTK2	*CERKL*	v_29_39	1.49(1.97)	1.90(2.29)	1.07(0.91-1.26)	0.39
PTK2	*CDK4*	v_29_40	1.36(1.97)	1.79(2.06)	1.06(0.90-1.24)	0.48

TRIB1	*SGK3*	v_30_37	1.50(1.03)	1.43(1.11)	1.02(0.69-1.50)	0.93
TRIB1	*MAGI2*	v_30_38	1.83(2.43)	1.59(1.35)	0.96(0.77-1.19)	0.69
PGK1	*STK32A*	v_31_32	1.34(1.07)	1.50(1.32)	1.10(0.78-1.54)	0.60
PGK1	*MAP2K1*	v_31_36	2.34(3.53)	3.63(3.64)	1.06(0.99-1.14)	0.10
PGK1	*SGK3*	v_31_37	2.44(2.19)	2.69(2.58)	1.05(0.89-1.24)	0.56
PGK1	*MAGI2*	v_31_38	2.11(1.74)	2.79(2.96)	1.07(0.93-1.23)	0.34
PGK1	*CERKL*	v_31_39	1.96(2.71)	2.66(3.99)	1.06(0.96-1.18)	0.25
STK32A	*PRKCB*	v_32_33	2.48(4.03)	3.06(4.08)	1.02(0.95-1.10)	0.60
STK32A	*MAGI2*	v_32_38	2.79(2.97)	3.67(4.45)	1.06(0.97-1.17)	0.19
STK32A	*CERKL*	v_32_39	2.10(2.62)	2.50(3.53)	1.04(0.93-1.17)	0.48
STK32A	*CDK4*	v_32_40	2.49(4.25)	2.32(2.78)	0.99(0.89-1.10)	0.87
PRKCB	*MYLK*	v_33_34	2.33(2.97)	2.09(2.51)	1.02(0.89-1.16)	0.80
PRKCB	*MAP2K1*	v_33_36	1.53(1.15)	1.84(1.88)	1.22(0.94-1.59)	0.13
PRKCB	*SGK3*	v_33_37	1.82(1.48)	2.06(1.96)	1.11(0.89-1.39)	0.36
PRKCB	*MAGI2*	v_33_38	1.65(1.17)	2.39(2.83)	1.12(0.96-1.30)	0.14
PRKCB	*CERKL*	v_33_39	1.21(1.07)	1.12(0.82)	0.91(0.59-1.40)	0.67
PRKCB	*CDK4*	v_33_40	1.18(0.89)	1.19(0.87)	1.01(0.66-1.55)	0.95
MYLK	*PI4K2B*	v_34_35	2.10(2.53)	1.60(2.13)	0.91(0.75-1.11)	0.36
MAP2K1	*SGK3*	v_36_37	1.82(1.92)	3.03(5.90)	1.06(0.98-1.14)	0.14
MAP2K1	*CDK4*	v_36_40	1.49(2.18)	1.55(2.03)	1.00(0.84-1.19)	0.98
SGK3	*MAGI2*	v_37_38	1.57(2.00)	1.87(1.92)	1.04(0.89-1.22)	0.61
CERKL	*CDK4*	v_39_40	1.11(0.57)	1.28(0.79)	1.54(0.82-2.88)	0.17
			Replication set			
MAP4K1	*STK39*	v_1_3	1.30(0.87)	0.99(0.70)	0.69(0.30-1.55)	0.36
MAP4K1	*DOLK*	v_1_12	1.34(1.24)	3.16(6.16)	1.25(1.07-1.47)	0.01
MAP4K1	*NEK4*	v_1_14	1.43(1.51)	2.22(3.56)	1.18(0.97-1.43)	0.10
MAP4K1	*RIOK2*	v_1_27	1.49(1.30)	3.45(7.09)	1.21(1.06-1.37)	0.004
MAP4K1	*PTK2*	v_1_29	1.45(1.55)	2.62(4.34)	1.17(1.01-1.35)	0.03
CDKL4	*STK39*	v_2_3	1.10(0.28)	0.93(0.22)	0.15(0.02-1.01)	0.05

CDKL4	*PRKD3*	v_2_4	1.20(0.85)	1.30(0.72)	0.96(0.52-1.75)	0.88
CDKL4	*AGK*	v_2_5	1.22(1.11)	1.34(0.55)	0.99(0.66-1.49)	0.97
CDKL4	*PIK3CA*	v_2_6	1.56(1.35)	1.17(0.84)	0.69(0.40-1.19)	0.18
CDKL4	*PKN3*	v_2_7	1.42(1.13)	1.64(1.16)	1.08(0.72-1.63)	0.70
CDKL4	*MST1R*	v_2_9	1.16(0.71)	1.57(1.10)	1.89(1.03-3.49)	0.04
CDKL4	*NEK8*	v_2_10	1.19(0.85)	1.81(1.02)	1.75(1.04-2.94)	0.04
CDKL4	*SPHK2*	v_2_11	1.20(0.84)	1.74(1.70)	1.59(1.05-2.42)	0.03
CDKL4	*BMPR1A*	v_2_13	1.30(1.44)	1.92(1.37)	1.23(0.92-1.64)	0.15
CDKL4	*NEK4*	v_2_14	1.21(0.86)	1.50(1.07)	1.37(0.82-2.29)	0.23
CDKL4	*BMPR1B*	v_2_16	1.16(0.90)	2.09(1.72)	1.72(1.19-2.49)	0.004
CDKL4	*DYRK4*	v_2_17	1.23(0.92)	2.05(2.73)	1.55(1.15-2.10)	0.004
CDKL4	*LIMK2*	v_2_18	1.20(0.81)	1.93(1.80)	1.78(1.20-2.64)	0.004
CDKL4	*AK7*	v_2_23	1.21(0.79)	1.54(1.62)	1.50(0.92-2.46)	0.11
CDKL4	*WNK1*	v_2_25	1.25(1.12)	1.97(2.29)	1.42(1.06-1.92)	0.02
CDKL4	*CDC7*	v_2_26	1.35(1.64)	2.94(4.50)	1.24(1.06-1.45)	0.01
CDKL4	*PTK2*	v_2_29	1.20(0.86)	1.82(1.46)	1.63(1.10-2.41)	0.01
CDKL4	*TRIB1*	v_2_30	1.41(1.73)	3.89(5.72)	1.32(1.11-1.57)	0.002
CDKL4	*MYLK*	v_2_34	1.16(0.72)	2.02(2.24)	1.98(1.25-3.13)	0.004
CDKL4	*MAP2K1*	v_2_36	1.20(0.81)	2.42(2.71)	1.74(1.25-2.44)	0.001
CDKL4	*SGK3*	v_2_37	1.15(0.80)	1.96(1.69)	1.66(1.17-2.37)	0.005
CDKL4	*MAGI2*	v_2_38	1.36(1.32)	2.40(2.36)	1.41(1.11-1.80)	0.01
CDKL4	*CERKL*	v_2_39	1.12(0.66)	1.67(1.27)	1.97(1.18-3.29)	0.01
STK39	*PRKD3*	v_3_4	1.14(0.82)	1.48(0.88)	1.20(0.73-1.95)	0.47
STK39	*AGK*	v_3_5	1.13(0.96)	1.49(0.60)	1.14(0.79-1.65)	0.49
STK39	*PKN3*	v_3_7	1.31(1.04)	1.88(1.56)	1.20(0.87-1.66)	0.26
STK39	*MST1R*	v_3_9	1.17(1.04)	1.73(1.36)	1.39(0.95-2.04)	0.09
STK39	*NEK8*	v_3_10	1.20(1.18)	2.02(1.23)	1.38(0.99-1.93)	0.06
STK39	*SPHK2*	v_3_11	1.23(1.27)	1.96(2.18)	1.30(0.98-1.71)	0.07
STK39	*BMPR1A*	v_3_13	1.44(2.30)	2.11(1.59)	1.09(0.90-1.32)	0.37

STK39	*NEK4*	v_3_14	1.24(1.30)	1.66(1.28)	1.18(0.86-1.63)	0.31
STK39	*BMPR1B*	v_3_16	1.21(1.42)	2.35(2.19)	1.32(1.03-1.68)	0.03
STK39	*DYRK4*	v_3_17	1.25(1.31)	2.35(3.43)	1.35(1.09-1.67)	0.01
STK39	*LIMK2*	v_3_18	1.18(1.03)	2.12(2.14)	1.59(1.17-2.16)	0.003
STK39	*AK7*	v_3_23	1.22(1.20)	1.78(2.04)	1.29(0.95-1.76)	0.10
STK39	*WNK1*	v_3_25	1.32(1.79)	2.24(2.84)	1.21(0.99-1.49)	0.06
STK39	*CDC7*	v_3_26	1.51(2.59)	3.42(5.68)	1.13(1.01-1.27)	0.03
STK39	*PTK2*	v_3_29	1.20(1.20)	1.96(1.61)	1.39(1.03-1.88)	0.03
STK39	*TRIB1*	v_3_30	1.55(2.64)	4.55(7.30)	1.18(1.06-1.30)	0.002
STK39	*MYLK*	v_3_34	1.15(1.00)	2.25(2.85)	1.55(1.17-2.06)	0.002
STK39	*MAP2K1*	v_3_36	1.19(1.10)	2.76(3.41)	1.50(1.17-1.92)	0.001
STK39	*SGK3*	v_3_37	1.16(1.19)	2.19(2.01)	1.44(1.10-1.90)	0.01
STK39	*MAGI2*	v_3_38	1.44(2.09)	2.70(2.97)	1.20(1.02-1.41)	0.03
STK39	*CERKL*	v_3_39	1.13(0.94)	1.86(1.53)	1.56(1.07-2.28)	0.02
STK39	*CDK4*	v_3_40	1.39(1.90)	2.80(3.84)	1.24(1.05-1.46)	0.01
PRKD3	*AGK*	v_4_5	1.24(0.86)	1.19(0.49)	1.12(0.52-2.40)	0.78
PRKD3	*WNK1*	v_4_25	1.54(2.08)	2.37(3.92)	1.26(1.04-1.52)	0.02
PRKD3	*SGK3*	v_4_37	1.39(1.57)	2.10(2.33)	1.46(1.09-1.95)	0.01
AGK	*DYRK4*	v_5_17	1.56(3.08)	1.64(2.06)	0.99(0.85-1.15)	0.91
AGK	*LIMK2*	v_5_18	1.47(2.33)	1.55(1.34)	0.99(0.81-1.21)	0.91
AGK	*PTK2*	v_5_29	1.58(3.22)	1.42(1.03)	0.96(0.80-1.16)	0.70
AGK	*MYLK*	v_5_34	1.50(2.74)	1.59(1.68)	0.99(0.84-1.17)	0.94
AGK	*SGK3*	v_5_37	1.81(4.75)	1.59(1.22)	0.97(0.85-1.11)	0.69
AGK	*MAGI2*	v_5_38	1.88(4.44)	1.90(1.77)	0.99(0.88-1.11)	0.81
HK2	*SPHK2*	v_8_11	1.13(0.49)	0.96(0.28)	0.49(0.15-1.64)	0.25
HK2	*BMPR1A*	v_8_13	1.06(0.46)	1.13(0.32)	1.30(0.42-4.06)	0.65
HK2	*NEK4*	v_8_14	1.19(0.62)	0.95(0.44)	0.56(0.19-1.59)	0.28
HK2	*PRKCD*	v_8_15	1.08(0.42)	1.02(0.26)	0.68(0.18-2.61)	0.57
HK2	*BMPR1B*	v_8_16	1.04(0.44)	1.15(0.24)	1.22(0.41-3.66)	0.73

HK2	*DYRK4*	v_8_17	1.16(0.56)	0.94(0.37)	0.54(0.16-1.83)	0.32
HK2	*LIMK2*	v_8_18	1.18(0.88)	1.01(0.35)	0.75(0.32-1.74)	0.51
HK2	*BMP2K*	v_8_22	1.09(0.55)	1.19(0.56)	1.19(0.55-2.59)	0.65
HK2	*WNK1*	v_8_25	1.17(0.71)	0.99(0.38)	0.66(0.22-1.98)	0.46
HK2	*CDC7*	v_8_26	1.09(0.57)	1.28(0.62)	1.36(0.59-3.09)	0.47
HK2	*PTK2*	v_8_29	1.12(0.53)	1.01(0.38)	0.66(0.20-2.16)	0.49
HK2	*TRIB1*	v_8_30	1.03(0.53)	1.58(0.98)	2.44(1.39-4.27)	0.002
HK2	*MYLK*	v_8_34	1.15(0.57)	1.00(0.25)	0.66(0.22-1.95)	0.45
HK2	*PI4K2B*	v_8_35	1.04(0.51)	1.45(0.82)	2.05(1.05-3.98)	0.03
HK2	*SGK3*	v_8_37	1.15(0.73)	1.11(0.45)	0.87(0.41-1.82)	0.70
HK2	*MAGI2*	v_8_38	1.22(0.54)	1.21(0.38)	0.97(0.38-2.45)	0.95
MST1R	*SPHK2*	v_9_11	1.02(0.26)	1.10(0.31)	2.13(0.26-17.24)	0.48
MST1R	*BMPR1A*	v_9_13	1.03(0.56)	1.30(0.41)	1.54(0.74-3.18)	0.25
MST1R	*NEK4*	v_9_14	1.08(0.43)	1.03(0.26)	0.93(0.24-3.67)	0.92
MST1R	*BMPR1B*	v_9_16	0.98(0.34)	1.33(0.36)	5.15(1.44-18.44)	0.01
MST1R	*DYRK4*	v_9_17	1.09(0.46)	1.08(0.48)	1.29(0.37-4.47)	0.69
MST1R	*LIMK2*	v_9_18	1.07(0.54)	1.15(0.38)	1.19(0.52-2.73)	0.68
MST1R	*NME4*	v_9_19	1.18(0.73)	1.91(1.80)	1.94(1.25-3.01)	0.003
MST1R	*AK7*	v_9_23	1.15(0.56)	0.97(0.44)	0.55(0.16-1.91)	0.35
MST1R	*ULK4*	v_9_24	1.10(0.65)	1.42(0.84)	1.61(0.82-3.15)	0.17
MST1R	*WNK1*	v_9_25	1.09(0.53)	1.16(0.48)	1.29(0.47-3.52)	0.62
MST1R	*CDC7*	v_9_26	1.08(0.65)	1.49(0.96)	1.77(0.93-3.36)	0.08
MST1R	*DGKB*	v_9_28	1.16(1.12)	1.89(1.20)	1.53(1.09-2.13)	0.01
MST1R	*PTK2*	v_9_29	1.09(0.53)	1.14(0.37)	1.09(0.40-2.97)	0.87
MST1R	*TRIB1*	v_9_30	1.06(0.70)	2.14(2.33)	1.54(1.21-1.96)	<0.001
MST1R	*MYLK*	v_9_34	1.07(0.47)	1.18(0.45)	1.50(0.56-3.98)	0.42
MST1R	*MAP2K1*	v_9_36	1.15(0.88)	1.45(0.65)	1.33(0.84-2.11)	0.23
MST1R	*SGK3*	v_9_37	1.06(0.49)	1.31(0.73)	1.93(0.83-4.49)	0.13
MST1R	*MAGI2*	v_9_38	1.16(0.54)	1.38(0.37)	2.11(0.76-5.80)	0.15

MST1R	*CERKL*	v_9_39	1.10(0.56)	1.11(0.40)	0.96(0.36-2.52)	0.93
MST1R	*CDK4*	v_9_40	1.13(0.52)	1.39(0.83)	2.11(0.92-4.87)	0.08
NEK8	*NEK4*	v_10_14	1.50(1.77)	0.90(0.46)	0.60(0.27-1.37)	0.23
NEK8	*BMPR1B*	v_10_16	1.31(1.24)	1.23(0.75)	0.89(0.54-1.47)	0.65
NEK8	*LIMK2*	v_10_18	1.33(1.33)	1.10(0.79)	0.83(0.47-1.49)	0.54
NEK8	*NME4*	v_10_19	1.52(1.78)	1.93(2.26)	1.11(0.90-1.37)	0.31
NEK8	*AK7*	v_10_23	1.39(1.24)	0.82(0.45)	0.38(0.12-1.17)	0.09
NEK8	*WNK1*	v_10_25	1.60(2.37)	1.10(0.78)	0.81(0.47-1.40)	0.44
NEK8	*CDC7*	v_10_26	1.58(2.02)	1.36(1.21)	0.92(0.64-1.32)	0.67
NEK8	*PTK2*	v_10_29	1.38(1.29)	1.11(0.80)	0.80(0.45-1.42)	0.44
NEK8	*PRKCB*	v_10_33	1.26(1.17)	1.14(0.71)	0.89(0.51-1.55)	0.68
NEK8	*MYLK*	v_10_34	1.35(1.28)	1.08(0.68)	0.79(0.42-1.47)	0.46
NEK8	*MAP2K1*	v_10_36	1.24(0.91)	1.23(0.74)	0.96(0.55-1.65)	0.87
NEK8	*CERKL*	v_10_39	1.49(1.91)	0.99(0.59)	0.67(0.32-1.37)	0.27
SPHK2	*BMPR1A*	v_11_13	1.09(0.67)	1.24(0.37)	1.23(0.59-2.58)	0.58
SPHK2	*NEK4*	v_11_14	1.13(0.62)	0.99(0.29)	0.72(0.23-2.30)	0.58
SPHK2	*BMPR1B*	v_11_16	0.99(0.33)	1.25(0.28)	4.43(1.00-19.66)	0.05
SPHK2	*DYRK4*	v_11_17	1.12(0.48)	1.02(0.41)	0.82(0.24-2.87)	0.76
SPHK2	*LIMK2*	v_11_18	1.06(0.44)	1.13(0.63)	1.26(0.51-3.11)	0.61
SPHK2	*LYN*	v_11_20	1.19(1.12)	1.34(0.67)	1.13(0.75-1.70)	0.56
SPHK2	*WNK1*	v_11_25	1.14(0.76)	1.09(0.41)	0.94(0.39-2.27)	0.89
SPHK2	*PTK2*	v_11_29	1.11(0.55)	1.12(0.48)	0.98(0.38-2.57)	0.97
SPHK2	*SGK3*	v_11_37	1.09(0.56)	1.23(0.61)	1.30(0.58-2.91)	0.52
DOLK	*BMPR1A*	v_12_13	1.20(1.15)	1.11(0.36)	0.91(0.46-1.77)	0.77
DOLK	*NEK4*	v_12_14	1.24(1.14)	0.88(0.29)	0.36(0.07-1.92)	0.23
DOLK	*BMPR1B*	v_12_16	1.02(0.29)	1.11(0.26)	1.77(0.32-9.85)	0.51
DOLK	*DYRK4*	v_12_17	1.21(0.78)	0.90(0.33)	0.45(0.12-1.72)	0.24
DOLK	*LIMK2*	v_12_18	1.11(0.48)	0.99(0.50)	0.72(0.23-2.29)	0.58
DOLK	*NME4*	v_12_19	1.18(0.63)	1.55(1.12)	2.05(1.08-3.88)	0.03

DOLK	*AK7*	v_12_23	1.24(0.75)	0.80(0.33)	0.17(0.03-0.99)	0.05
DOLK	*WNK1*	v_12_25	1.29(1.65)	0.97(0.38)	0.66(0.19-2.31)	0.52
DOLK	*RIOK2*	v_12_27	1.22(0.54)	0.97(0.50)	0.43(0.14-1.38)	0.16
DOLK	*PTK2*	v_12_29	1.16(0.62)	0.98(0.38)	0.59(0.18-1.86)	0.37
DOLK	*TRIB1*	v_12_30	1.13(0.83)	1.65(1.47)	1.61(1.09-2.38)	0.02
DOLK	*PGK1*	v_12_31	1.19(0.59)	0.99(0.40)	0.60(0.21-1.69)	0.33
DOLK	*STK32A*	v_12_32	2.02(2.74)	1.09(0.84)	0.84(0.58-1.20)	0.33
DOLK	*SGK3*	v_12_37	1.12(0.55)	1.09(0.53)	0.95(0.35-2.61)	0.93
DOLK	*MAGI2*	v_12_38	1.21(0.60)	1.15(0.29)	1.01(0.36-2.84)	0.98
DOLK	*CERKL*	v_12_39	1.28(1.35)	0.93(0.30)	0.49(0.13-1.78)	0.27
DOLK	*CDK4*	v_12_40	1.25(0.97)	1.11(0.54)	0.90(0.42-1.97)	0.80
BMPR1A	*NEK4*	v_13_14	1.17(0.56)	0.84(0.25)	0.22(0.04-1.11)	0.07
BMPR1A	*PRKCD*	v_13_15	1.10(0.42)	0.94(0.24)	0.39(0.08-1.83)	0.23
BMPR1A	*BMPR1B*	v_13_16	1.05(0.33)	1.05(0.23)	0.89(0.18-4.47)	0.88
BMPR1A	*DYRK4*	v_13_17	1.19(0.56)	0.91(0.58)	0.50(0.15-1.71)	0.27
BMPR1A	*LIMK2*	v_13_18	1.26(0.79)	0.98(0.55)	0.68(0.30-1.53)	0.35
BMPR1A	*WNK1*	v_13_25	1.14(0.49)	0.96(0.50)	0.54(0.14-2.06)	0.36
BMPR1A	*PTK2*	v_13_29	1.19(0.64)	0.92(0.33)	0.45(0.13-1.51)	0.20
BMPR1A	*SGK3*	v_13_37	1.20(0.71)	0.99(0.31)	0.65(0.26-1.60)	0.35
BMPR1A	*MAGI2*	v_13_38	1.30(0.66)	1.15(0.50)	0.78(0.34-1.78)	0.56
NEK4	*BMPR1B*	v_14_16	1.00(0.44)	1.35(0.42)	1.77(0.78-4.02)	0.17
NEK4	*DYRK4*	v_14_17	1.11(0.49)	1.11(0.61)	1.02(0.36-2.92)	0.96
NEK4	*LIMK2*	v_14_18	1.13(0.60)	1.23(0.73)	1.13(0.53-2.41)	0.75
NEK4	*NME4*	v_14_19	1.22(0.86)	2.04(2.17)	1.56(1.14-2.15)	0.01
NEK4	*AK7*	v_14_23	1.12(0.47)	0.94(0.34)	0.33(0.07-1.60)	0.17
NEK4	*WNK1*	v_14_25	1.05(0.44)	1.16(0.52)	1.14(0.44-2.93)	0.79
NEK4	*CDC7*	v_14_26	1.04(0.56)	1.50(1.04)	2.01(1.00-4.04)	0.05
NEK4	*DGKB*	v_14_28	1.15(0.89)	2.01(1.45)	1.94(1.29-2.92)	0.001
NEK4	*PTK2*	v_14_29	1.10(0.59)	1.19(0.53)	1.00(0.46-2.19)	1.00

NEK4	*TRIB1*	v_14_30	1.03(0.66)	2.29(2.56)	1.49(1.20-1.85)	<0.001
NEK4	*PRKCB*	v_14_33	1.12(0.62)	1.28(0.52)	1.24(0.58-2.64)	0.58
NEK4	*MYLK*	v_14_34	1.10(0.60)	1.23(0.55)	1.14(0.56-2.34)	0.71
NEK4	*MAP2K1*	v_14_36	1.12(0.71)	1.47(0.70)	1.60(0.91-2.80)	0.10
NEK4	*SGK3*	v_14_37	1.06(0.53)	1.32(0.77)	1.61(0.70-3.69)	0.26
NEK4	*MAGI2*	v_14_38	1.20(0.61)	1.44(0.58)	1.53(0.67-3.47)	0.31
NEK4	*CERKL*	v_14_39	1.07(0.51)	1.11(0.37)	0.85(0.33-2.17)	0.74
NEK4	*CDK4*	v_14_40	1.14(0.56)	1.38(0.86)	1.79(0.82-3.92)	0.14
PRKCD	*BMPR1B*	v_15_16	1.00(0.27)	1.15(0.20)	3.99(0.55-29.10)	0.17
PRKCD	*LIMK2*	v_15_18	1.14(0.55)	1.07(0.59)	0.86(0.34-2.17)	0.74
PRKCD	*BMP2K*	v_15_22	1.07(0.46)	1.26(0.80)	2.02(0.78-5.27)	0.15
PRKCD	*WNK1*	v_15_25	1.08(0.41)	0.99(0.33)	0.64(0.11-3.75)	0.62
PRKCD	*PTK2*	v_15_29	1.09(0.40)	1.03(0.41)	0.77(0.20-2.88)	0.69
PRKCD	*MYLK*	v_15_34	1.11(0.49)	1.03(0.34)	0.88(0.27-2.92)	0.84
PRKCD	*PI4K2B*	v_15_35	1.02(0.50)	1.46(0.79)	2.84(1.31-6.16)	0.01
PRKCD	*SGK3*	v_15_37	1.09(0.52)	1.08(0.33)	0.95(0.34-2.63)	0.92
PRKCD	*MAGI2*	v_15_38	1.18(0.47)	1.24(0.42)	1.28(0.45-3.66)	0.64
BMPR1B	*LIMK2*	v_16_18	1.16(0.52)	0.94(0.56)	0.59(0.19-1.79)	0.35
BMPR1B	*BMP2K*	v_16_22	1.11(0.46)	1.11(0.71)	1.32(0.45-3.90)	0.62
BMPR1B	*CDC7*	v_16_26	1.13(0.78)	1.14(0.61)	1.05(0.50-2.21)	0.90
BMPR1B	*PTK2*	v_16_29	1.16(0.53)	0.90(0.35)	0.40(0.10-1.54)	0.18
BMPR1B	*MYLK*	v_16_34	1.17(0.55)	0.90(0.25)	0.43(0.11-1.73)	0.23
BMPR1B	*PI4K2B*	v_16_35	1.08(0.59)	1.30(0.72)	1.74(0.80-3.79)	0.16
DYRK4	*LIMK2*	v_17_18	1.04(0.39)	1.19(0.51)	1.83(0.57-5.89)	0.31
DYRK4	*NME4*	v_17_19	1.14(0.66)	1.65(0.79)	2.22(1.18-4.18)	0.01
DYRK4	*AK7*	v_17_23	1.13(0.55)	0.96(0.39)	0.44(0.10-1.97)	0.28
DYRK4	*WNK1*	v_17_25	1.08(0.50)	1.11(0.32)	0.95(0.31-2.89)	0.93
DYRK4	*CDC7*	v_17_26	1.09(0.73)	1.37(0.50)	1.18(0.66-2.09)	0.58
DYRK4	*DGKB*	v_17_28	1.18(1.01)	1.92(1.24)	1.45(1.02-2.05)	0.04

DYRK4	*PTK2*	v_17_29	1.05(0.53)	1.25(0.79)	1.30(0.66-2.59)	0.45
DYRK4	*TRIB1*	v_17_30	1.05(0.69)	2.06(2.16)	1.52(1.18-1.95)	0.001
DYRK4	*PRKCB*	v_17_33	1.13(0.77)	1.30(0.69)	1.11(0.64-1.90)	0.71
DYRK4	*MYLK*	v_17_34	1.08(0.52)	1.26(0.71)	1.47(0.67-3.26)	0.34
DYRK4	*MAP2K1*	v_17_36	1.19(0.93)	1.56(1.01)	1.26(0.82-1.93)	0.29
DYRK4	*SGK3*	v_17_37	1.13(0.77)	1.40(0.91)	1.24(0.71-2.14)	0.45
DYRK4	*MAGI2*	v_17_38	1.21(0.71)	1.48(0.85)	1.32(0.74-2.34)	0.34
DYRK4	*CERKL*	v_17_39	1.09(0.57)	1.16(0.53)	0.99(0.41-2.36)	0.98
DYRK4	*CDK4*	v_17_40	1.17(0.64)	1.35(0.80)	1.33(0.68-2.62)	0.41
LIMK2	*BMP2K*	v_18_22	1.11(0.70)	1.23(0.60)	1.30(0.61-2.77)	0.50
LIMK2	*PI4K2B*	v_18_35	1.19(0.94)	1.58(0.96)	1.23(0.77-1.95)	0.38
LYN	*CDC7*	v_20_26	1.08(0.45)	1.11(0.46)	0.98(0.29-3.27)	0.97
LYN	*DGKB*	v_20_28	1.25(1.01)	1.57(1.08)	1.37(0.86-2.16)	0.18
LYN	*PTK2*	v_20_29	1.16(0.56)	0.99(0.53)	0.61(0.20-1.83)	0.37
LYN	*TRIB1*	v_20_30	1.05(0.49)	1.56(1.41)	1.81(1.20-2.73)	0.005
LYN	*PRKCB*	v_20_33	1.30(0.87)	1.06(0.66)	0.76(0.35-1.63)	0.48
LYN	*MYLK*	v_20_34	1.29(0.84)	1.02(0.68)	0.72(0.31-1.66)	0.44
LYN	*MAP2K1*	v_20_36	1.37(1.04)	1.33(1.07)	1.04(0.62-1.76)	0.87
LYN	*SGK3*	v_20_37	1.22(0.73)	1.16(0.82)	0.95(0.43-2.09)	0.90
LYN	*MAGI2*	v_20_38	1.33(0.76)	1.21(0.75)	0.90(0.43-1.88)	0.77
LYN	*CDK4*	v_20_40	1.29(0.71)	1.16(0.85)	0.95(0.42-2.12)	0.89
NEK9	*PGK1*	v_21_31	1.27(0.92)	1.22(0.92)	0.93(0.56-1.56)	0.79
BMP2K	*PI4K2B*	v_22_35	1.13(0.75)	1.40(0.95)	1.20(0.67-2.16)	0.53
AK7	*ULK4*	v_23_24	1.02(0.48)	1.52(0.80)	2.99(1.40-6.38)	0.005
AK7	*WNK1*	v_23_25	1.03(0.47)	1.30(0.59)	1.95(0.83-4.60)	0.13
AK7	*CDC7*	v_23_26	1.06(0.62)	1.54(0.51)	2.14(1.04-4.41)	0.04
AK7	*DGKB*	v_23_28	1.16(0.99)	2.16(1.09)	2.07(1.35-3.17)	<0.001
AK7	*PTK2*	v_23_29	1.03(0.42)	1.42(0.77)	2.57(1.10-6.02)	0.03
AK7	*TRIB1*	v_23_30	1.05(0.71)	2.68(3.72)	1.28(1.11-1.48)	<0.001

AK7	*PRKCB*	v_23_33	1.05(0.55)	1.43(0.51)	2.20(1.06-4.57)	0.04
AK7	*MYLK*	v_23_34	1.04(0.50)	1.41(0.68)	2.19(1.02-4.68)	0.04
AK7	*MAP2K1*	v_23_36	1.05(0.55)	1.67(0.82)	2.89(1.52-5.47)	0.001
AK7	*SGK3*	v_23_37	1.05(0.56)	1.64(1.33)	1.83(1.18-2.84)	0.01
AK7	*MAGI2*	v_23_38	1.17(0.66)	1.64(0.68)	2.06(1.10-3.84)	0.02
AK7	*CERKL*	v_23_39	1.05(0.51)	1.30(0.61)	1.64(0.73-3.69)	0.23
AK7	*CDK4*	v_23_40	1.12(0.58)	1.56(1.04)	2.26(1.22-4.16)	0.01
ULK4	*WNK1*	v_24_25	1.09(0.37)	0.91(0.23)	0.27(0.05-1.66)	0.16
ULK4	*CDC7*	v_24_26	1.02(0.31)	1.16(0.43)	2.15(0.53-8.73)	0.28
ULK4	*DGKB*	v_24_28	1.20(0.93)	1.71(1.27)	1.59(1.04-2.41)	0.03
ULK4	*PTK2*	v_24_29	1.14(0.55)	1.10(0.79)	0.98(0.38-2.55)	0.97
ULK4	*TRIB1*	v_24_30	1.04(0.55)	1.58(1.08)	2.78(1.58-4.90)	<0.001
ULK4	*PRKCB*	v_24_33	1.22(0.77)	1.13(0.66)	0.93(0.45-1.91)	0.84
ULK4	*MYLK*	v_24_34	1.20(0.63)	1.10(0.73)	0.94(0.39-2.27)	0.90
ULK4	*MAP2K1*	v_24_36	1.22(0.72)	1.37(1.01)	1.44(0.74-2.79)	0.28
ULK4	*SGK3*	v_24_37	1.15(0.68)	1.19(0.78)	1.14(0.54-2.42)	0.74
ULK4	*MAGI2*	v_24_38	1.26(0.69)	1.29(0.82)	1.15(0.56-2.33)	0.71
ULK4	*CERKL*	v_24_39	1.15(0.49)	0.99(0.53)	0.59(0.18-1.87)	0.37
ULK4	*CDK4*	v_24_40	1.22(0.63)	1.16(0.74)	1.04(0.45-2.41)	0.93
WNK1	*CDC7*	v_25_26	1.00(0.36)	1.30(0.44)	3.14(0.87-11.35)	0.08
WNK1	*DGKB*	v_25_28	1.20(0.91)	1.87(1.26)	1.80(1.14-2.85)	0.01
WNK1	*PTK2*	v_25_29	1.09(0.45)	1.20(0.78)	1.50(0.55-4.14)	0.43
WNK1	*TRIB1*	v_25_30	1.02(0.55)	1.81(1.34)	2.42(1.57-3.72)	<0.001
WNK1	*PRKCB*	v_25_33	1.14(0.55)	1.23(0.60)	1.25(0.52-2.97)	0.62
WNK1	*MYLK*	v_25_34	1.13(0.56)	1.19(0.68)	1.32(0.56-3.11)	0.52
WNK1	*MAP2K1*	v_25_36	1.15(0.64)	1.42(0.79)	1.66(0.82-3.36)	0.16
WNK1	*SGK3*	v_25_37	1.08(0.53)	1.26(0.69)	1.54(0.65-3.66)	0.33
WNK1	*MAGI2*	v_25_38	1.20(0.58)	1.40(0.75)	1.58(0.72-3.49)	0.26
WNK1	*CERKL*	v_25_39	1.08(0.41)	1.06(0.40)	0.83(0.21-3.25)	0.79

WNK1	*CDK4*	v_25_40	1.16(0.57)	1.24(0.68)	1.34(0.58-3.11)	0.49
CDC7	*PRKCB*	v_26_33	1.27(0.75)	0.97(0.37)	0.63(0.25-1.58)	0.32
CDC7	*CERKL*	v_26_39	1.20(0.57)	0.90(0.44)	0.42(0.13-1.38)	0.15
CDC7	*CDK4*	v_26_40	1.24(0.68)	1.05(0.77)	0.82(0.32-2.09)	0.68
DGKB	*PTK2*	v_28_29	1.54(1.68)	0.83(0.59)	0.41(0.15-1.18)	0.10
DGKB	*TRIB1*	v_28_30	1.48(2.18)	1.39(1.87)	0.99(0.74-1.32)	0.95
DGKB	*MYLK*	v_28_34	1.55(1.73)	0.78(0.50)	0.34(0.11-1.05)	0.06
DGKB	*MAP2K1*	v_28_36	1.32(1.06)	0.90(0.45)	0.46(0.15-1.38)	0.17
DGKB	*SGK3*	v_28_37	1.49(1.50)	0.90(0.68)	0.47(0.19-1.16)	0.10
DGKB	*MAGI2*	v_28_38	1.54(1.55)	0.91(0.49)	0.46(0.17-1.21)	0.11
PTK2	*TRIB1*	v_29_30	1.01(0.56)	1.83(1.66)	2.23(1.48-3.37)	<0.001
PTK2	*PRKCB*	v_29_33	1.16(1.00)	1.17(0.48)	0.99(0.58-1.69)	0.97
PTK2	*MYLK*	v_29_34	1.08(0.43)	1.10(0.49)	1.46(0.40-5.38)	0.57
PTK2	*MAP2K1*	v_29_36	1.16(0.72)	1.44(0.82)	1.43(0.79-2.58)	0.24
PTK2	*SGK3*	v_29_37	1.06(0.50)	1.17(0.48)	1.23(0.53-2.86)	0.63
PTK2	*MAGI2*	v_29_38	1.20(0.60)	1.30(0.49)	1.24(0.56-2.77)	0.59
PTK2	*CERKL*	v_29_39	1.10(0.51)	1.06(0.43)	0.85(0.26-2.74)	0.78
PTK2	*CDK4*	v_29_40	1.17(0.52)	1.35(0.85)	1.77(0.77-4.09)	0.18
TRIB1	*SGK3*	v_30_37	1.29(0.85)	0.83(0.39)	0.27(0.07-1.00)	0.05
TRIB1	*MAGI2*	v_30_38	1.45(0.92)	0.94(0.38)	0.40(0.15-1.04)	0.06
PGK1	*STK32A*	v_31_32	1.82(2.52)	1.06(0.41)	0.81(0.53-1.22)	0.31
PGK1	*MAP2K1*	v_31_36	1.17(0.84)	1.41(0.81)	1.17(0.69-1.98)	0.57
PGK1	*SGK3*	v_31_37	1.52(3.44)	1.21(0.64)	0.93(0.71-1.23)	0.63
PGK1	*MAGI2*	v_31_38	1.66(3.72)	1.27(0.44)	0.93(0.71-1.23)	0.63
PGK1	*CERKL*	v_31_39	1.38(2.19)	1.07(0.55)	0.84(0.46-1.55)	0.58
STK32A	*PRKCB*	v_32_33	1.37(0.93)	1.21(0.57)	0.83(0.43-1.59)	0.57
STK32A	*MAGI2*	v_32_38	1.67(1.36)	1.37(0.72)	0.87(0.54-1.41)	0.57
STK32A	*CERKL*	v_32_39	1.80(2.40)	1.14(0.72)	0.75(0.44-1.25)	0.27
STK32A	*CDK4*	v_32_40	1.81(1.86)	1.41(1.26)	0.89(0.61-1.30)	0.55

PRKCB	*MYLK*	v_33_34	1.19(0.82)	0.99(0.33)	0.64(0.23-1.82)	0.41
PRKCB	*MAP2K1*	v_33_36	1.10(0.58)	1.25(0.58)	1.35(0.67-2.72)	0.40
PRKCB	*SGK3*	v_33_37	1.16(0.75)	1.18(0.90)	1.03(0.50-2.13)	0.93
PRKCB	*MAGI2*	v_33_38	1.26(0.90)	1.16(0.26)	0.92(0.43-2.01)	0.84
PRKCB	*CERKL*	v_33_39	1.44(2.09)	0.97(0.42)	0.64(0.25-1.65)	0.36
PRKCB	*CDK4*	v_33_40	1.39(1.48)	1.16(0.73)	0.90(0.52-1.57)	0.71
MYLK	*PI4K2B*	v_34_35	1.10(0.75)	1.47(0.72)	1.46(0.82-2.62)	0.20
MAP2K1	*SGK3*	v_36_37	1.28(1.15)	1.01(0.56)	0.66(0.28-1.52)	0.32
MAP2K1	*CDK4*	v_36_40	1.51(1.84)	0.94(0.51)	0.55(0.21-1.39)	0.20
SGK3	*MAGI2*	v_37_38	1.20(0.55)	1.23(0.53)	1.14(0.48-2.73)	0.77
CERKL	*CDK4*	v_39_40	1.14(0.51)	1.18(0.57)	1.43(0.52-3.90)	0.49
			Pooled analysis			
MAP4K1	*STK39*	v_1_3	1.77(1.80)	1.94(2.51)	1.02(0.88-1.19)	0.78
MAP4K1	*DOLK*	v_1_12	1.22(1.02)	2.15(4.06)	1.15(1.05-1.25)	0.002
MAP4K1	*NEK4*	v_1_14	1.64(1.63)	2.49(3.23)	1.17(1.04-1.31)	0.01
MAP4K1	*RIOK2*	v_1_27	1.52(1.38)	2.27(4.37)	1.13(1.04-1.23)	0.01
MAP4K1	*PTK2*	v_1_29	1.75(2.21)	2.63(3.45)	1.09(1.00-1.19)	0.05
CDKL4	*STK39*	v_2_3	1.12(0.35)	1.14(0.51)	1.05(0.49-2.28)	0.90
CDKL4	*PRKD3*	v_2_4	1.57(1.26)	1.84(1.45)	1.08(0.87-1.35)	0.49
CDKL4	*AGK*	v_2_5	1.16(0.91)	1.48(0.74)	1.23(0.99-1.54)	0.07
CDKL4	*PIK3CA*	v_2_6	2.42(3.19)	2.24(2.92)	0.98(0.88-1.08)	0.64
CDKL4	*PKN3*	v_2_7	2.02(2.98)	2.35(2.89)	1.02(0.94-1.11)	0.65
CDKL4	*MST1R*	v_2_9	1.27(1.80)	1.63(2.35)	1.05(0.94-1.17)	0.40
CDKL4	*NEK8*	v_2_10	1.71(3.38)	2.22(2.25)	1.02(0.95-1.09)	0.57
CDKL4	*SPHK2*	v_2_11	1.34(1.07)	2.18(2.96)	1.14(1.04-1.25)	0.01
CDKL4	*BMPR1A*	v_2_13	1.36(1.39)	2.20(3.56)	1.09(1.01-1.18)	0.03
CDKL4	*NEK4*	v_2_14	1.12(0.66)	1.58(2.17)	1.15(1.01-1.31)	0.04
CDKL4	*BMPR1B*	v_2_16	1.31(1.46)	2.13(3.81)	1.08(1.00-1.16)	0.04
CDKL4	*DYRK4*	v_2_17	1.15(0.74)	1.52(1.76)	1.35(1.09-1.67)	0.01

CDKL4	*LIMK2*	v_2_18	1.38(1.46)	2.27(4.52)	1.06(1.00-1.14)	0.06
CDKL4	*AK7*	v_2_23	1.12(0.91)	1.94(3.22)	1.11(1.02-1.21)	0.02
CDKL4	*WNK1*	v_2_25	1.12(0.85)	1.63(1.49)	1.48(1.21-1.82)	<0.001
CDKL4	*CDC7*	v_2_26	1.24(1.38)	2.41(3.59)	1.18(1.08-1.29)	<0.001
CDKL4	*PTK2*	v_2_29	1.10(0.70)	1.65(1.73)	1.27(1.10-1.48)	0.002
CDKL4	*TRIB1*	v_2_30	1.31(1.40)	2.42(3.62)	1.19(1.09-1.30)	<0.001
CDKL4	*MYLK*	v_2_34	1.55(2.01)	2.48(4.03)	1.07(1.00-1.15)	0.04
CDKL4	*MAP2K1*	v_2_36	1.35(1.85)	2.75(4.12)	1.09(1.03-1.16)	0.01
CDKL4	*SGK3*	v_2_37	1.23(0.83)	1.83(1.33)	1.64(1.25-2.14)	<0.001
CDKL4	*MAGI2*	v_2_38	1.27(1.04)	2.10(1.71)	1.38(1.18-1.62)	<0.001
CDKL4	*CERKL*	v_2_39	1.10(0.80)	2.03(2.49)	1.21(1.07-1.35)	0.001
STK39	*PRKD3*	v_3_4	1.41(0.98)	1.68(1.12)	1.14(0.87-1.51)	0.34
STK39	*AGK*	v_3_5	1.08(0.79)	1.44(0.82)	1.35(1.06-1.73)	0.01
STK39	*PKN3*	v_3_7	1.86(2.55)	2.05(2.48)	1.01(0.91-1.13)	0.80
STK39	*MST1R*	v_3_9	1.27(1.69)	1.48(1.39)	1.05(0.90-1.24)	0.51
STK39	*NEK8*	v_3_10	1.58(2.46)	2.30(2.62)	1.07(0.97-1.17)	0.18
STK39	*SPHK2*	v_3_11	1.47(2.20)	1.86(1.75)	1.06(0.95-1.18)	0.28
STK39	*BMPR1A*	v_3_13	1.62(3.07)	1.89(1.64)	1.03(0.94-1.12)	0.54
STK39	*NEK4*	v_3_14	1.14(1.05)	1.40(1.13)	1.20(0.95-1.52)	0.13
STK39	*BMPR1B*	v_3_16	1.40(2.01)	1.85(1.94)	1.09(0.97-1.23)	0.16
STK39	*DYRK4*	v_3_17	1.13(0.99)	1.50(2.14)	1.23(1.04-1.46)	0.02
STK39	*LIMK2*	v_3_18	1.45(2.05)	1.90(2.13)	1.08(0.96-1.21)	0.21
STK39	*AK7*	v_3_23	1.17(1.46)	1.73(1.84)	1.16(1.00-1.35)	0.04
STK39	*WNK1*	v_3_25	1.16(1.34)	1.68(1.89)	1.22(1.06-1.41)	0.01
STK39	*CDC7*	v_3_26	1.42(2.43)	2.49(3.91)	1.11(1.03-1.21)	0.01
STK39	*PTK2*	v_3_29	1.09(0.94)	1.53(1.28)	1.37(1.09-1.71)	0.01
STK39	*TRIB1*	v_3_30	1.38(2.04)	2.57(4.60)	1.13(1.05-1.21)	0.001
STK39	*MYLK*	v_3_34	1.66(2.88)	2.23(2.52)	1.05(0.97-1.14)	0.19
STK39	*MAP2K1*	v_3_36	1.46(2.78)	2.58(3.21)	1.07(1.00-1.15)	0.04

STK39	*SGK3*	v_3_37	1.17(0.96)	1.73(1.42)	1.41(1.14-1.75)	0.002
STK39	*MAGI2*	v_3_38	1.27(1.57)	2.05(2.10)	1.18(1.05-1.32)	0.005
STK39	*CERKL*	v_3_39	1.18(1.32)	1.97(2.02)	1.22(1.05-1.43)	0.01
STK39	*CDK4*	v_3_40	1.40(2.28)	2.40(3.49)	1.10(1.01-1.20)	0.03
PRKD3	*AGK*	v_4_5	1.47(2.45)	1.69(2.00)	1.00(0.90-1.12)	0.93
PRKD3	*WNK1*	v_4_25	1.56(2.19)	2.30(4.24)	1.06(0.98-1.14)	0.16
PRKD3	*SGK3*	v_4_37	1.43(1.99)	1.70(1.84)	1.04(0.91-1.19)	0.54
AGK	*DYRK4*	v_5_17	1.61(2.77)	1.19(1.35)	0.90(0.72-1.12)	0.36
AGK	*LIMK2*	v_5_18	1.73(2.55)	1.66(2.85)	0.99(0.87-1.11)	0.81
AGK	*PTK2*	v_5_29	1.54(2.74)	1.39(1.50)	0.97(0.84-1.12)	0.65
AGK	*MYLK*	v_5_34	1.97(3.08)	1.76(2.53)	0.98(0.87-1.09)	0.69
AGK	*SGK3*	v_5_37	1.54(3.43)	1.37(0.93)	0.98(0.87-1.11)	0.79
AGK	*MAGI2*	v_5_38	1.66(3.27)	1.75(1.58)	1.00(0.91-1.10)	0.93
HK2	*SPHK2*	v_8_11	1.32(0.85)	1.40(1.17)	1.03(0.75-1.41)	0.87
HK2	*BMPR1A*	v_8_13	1.18(0.69)	1.26(0.67)	1.06(0.70-1.60)	0.79
HK2	*NEK4*	v_8_14	1.48(1.75)	1.85(3.13)	1.02(0.91-1.15)	0.72
HK2	*PRKCD*	v_8_15	1.15(0.55)	1.09(0.58)	0.77(0.45-1.31)	0.34
HK2	*BMPR1B*	v_8_16	1.25(0.86)	1.34(0.97)	1.02(0.75-1.39)	0.90
HK2	*DYRK4*	v_8_17	1.48(1.62)	1.62(2.30)	0.99(0.85-1.16)	0.94
HK2	*LIMK2*	v_8_18	1.22(0.80)	1.09(0.55)	0.79(0.49-1.25)	0.31
HK2	*BMP2K*	v_8_22	1.21(0.75)	1.25(0.66)	1.06(0.73-1.53)	0.77
HK2	*WNK1*	v_8_25	1.44(1.70)	1.83(2.69)	1.04(0.92-1.17)	0.58
HK2	*CDC7*	v_8_26	1.32(1.30)	2.28(5.04)	1.05(0.98-1.12)	0.19
HK2	*PTK2*	v_8_29	1.36(1.43)	1.80(2.60)	1.05(0.92-1.20)	0.45
HK2	*TRIB1*	v_8_30	1.41(1.80)	2.35(3.76)	1.06(0.97-1.16)	0.20
HK2	*MYLK*	v_8_34	1.36(1.16)	1.17(0.88)	0.89(0.65-1.20)	0.43
HK2	*PI4K2B*	v_8_35	1.48(1.27)	1.72(1.62)	1.04(0.85-1.28)	0.69
HK2	*SGK3*	v_8_37	1.64(2.09)	2.34(3.60)	1.04(0.95-1.14)	0.43
HK2	*MAGI2*	v_8_38	1.66(2.21)	2.61(4.62)	1.04(0.97-1.12)	0.30

MST1R	*SPHK2*	v_9_11	1.39(1.20)	2.03(1.77)	1.25(1.04-1.50)	0.02
MST1R	*BMPR1A*	v_9_13	1.33(1.24)	1.93(1.68)	1.24(1.04-1.49)	0.02
MST1R	*NEK4*	v_9_14	1.24(0.70)	1.56(1.39)	1.17(0.93-1.48)	0.18
MST1R	*BMPR1B*	v_9_16	1.47(2.41)	1.74(1.41)	1.03(0.93-1.14)	0.58
MST1R	*DYRK4*	v_9_17	1.29(0.84)	1.51(1.34)	1.13(0.86-1.47)	0.38
MST1R	*LIMK2*	v_9_18	1.50(2.29)	1.74(1.49)	1.03(0.92-1.15)	0.64
MST1R	*NME4*	v_9_19	1.46(1.37)	2.14(2.38)	1.22(1.06-1.41)	0.01
MST1R	*AK7*	v_9_23	1.09(0.55)	1.21(0.47)	1.47(0.85-2.56)	0.17
MST1R	*ULK4*	v_9_24	1.11(0.69)	1.52(0.98)	1.53(1.12-2.09)	0.01
MST1R	*WNK1*	v_9_25	1.25(0.75)	1.71(1.29)	1.45(1.11-1.90)	0.01
MST1R	*CDC7*	v_9_26	1.23(0.90)	1.55(1.13)	1.38(1.03-1.84)	0.03
MST1R	*DGKB*	v_9_28	1.29(1.06)	1.92(2.68)	1.11(1.01-1.23)	0.04
MST1R	*PTK2*	v_9_29	1.23(0.79)	1.63(1.32)	1.27(0.99-1.62)	0.06
MST1R	*TRIB1*	v_9_30	1.31(1.10)	1.96(1.76)	1.28(1.06-1.54)	0.01
MST1R	*MYLK*	v_9_34	1.81(3.43)	2.20(2.45)	1.02(0.96-1.10)	0.49
MST1R	*MAP2K1*	v_9_36	1.35(1.12)	1.99(1.73)	1.37(1.13-1.65)	0.001
MST1R	*SGK3*	v_9_37	1.48(1.21)	2.19(2.23)	1.22(1.03-1.45)	0.02
MST1R	*MAGI2*	v_9_38	1.46(1.10)	2.39(2.40)	1.18(1.04-1.35)	0.01
MST1R	*CERKL*	v_9_39	1.14(0.79)	1.37(0.91)	1.18(0.90-1.55)	0.23
MST1R	*CDK4*	v_9_40	1.24(1.00)	1.52(0.91)	1.17(0.92-1.48)	0.20
NEK8	*NEK4*	v_10_14	1.94(2.49)	1.86(3.14)	0.96(0.84-1.08)	0.47
NEK8	*BMPR1B*	v_10_16	1.84(2.79)	1.92(3.52)	0.99(0.89-1.09)	0.82
NEK8	*LIMK2*	v_10_18	1.92(3.08)	1.57(2.35)	0.94(0.82-1.08)	0.36
NEK8	*NME4*	v_10_19	1.66(2.68)	1.50(1.55)	0.97(0.85-1.12)	0.71
NEK8	*AK7*	v_10_23	1.73(2.39)	1.25(1.25)	0.86(0.70-1.06)	0.15
NEK8	*WNK1*	v_10_25	1.87(2.68)	2.02(3.64)	1.00(0.90-1.11)	0.96
NEK8	*CDC7*	v_10_26	1.59(2.00)	1.33(1.21)	0.92(0.75-1.12)	0.39
NEK8	*PTK2*	v_10_29	1.82(2.42)	1.93(3.25)	0.98(0.88-1.10)	0.75
NEK8	*PRKCB*	v_10_33	1.78(2.93)	1.47(1.28)	0.92(0.79-1.08)	0.32

NEK8	*MYLK*	v_10_34	1.90(2.37)	2.02(3.80)	1.00(0.90-1.12)	1.00
NEK8	*MAP2K1*	v_10_36	1.62(2.22)	1.36(0.81)	0.90(0.73-1.11)	0.32
NEK8	*CERKL*	v_10_39	2.06(3.22)	1.51(1.96)	0.90(0.78-1.04)	0.15
SPHK2	*BMPR1A*	v_11_13	1.11(0.68)	1.20(0.57)	1.09(0.72-1.67)	0.68
SPHK2	*NEK4*	v_11_14	1.52(2.11)	1.61(2.38)	0.98(0.85-1.12)	0.72
SPHK2	*BMPR1B*	v_11_16	1.40(2.02)	1.32(1.17)	0.95(0.79-1.13)	0.55
SPHK2	*DYRK4*	v_11_17	1.71(2.62)	1.71(2.76)	0.96(0.85-1.09)	0.54
SPHK2	*LIMK2*	v_11_18	1.27(1.37)	1.16(0.86)	0.90(0.67-1.21)	0.49
SPHK2	*LYN*	v_11_20	1.54(2.61)	1.83(3.28)	1.01(0.92-1.11)	0.90
SPHK2	*WNK1*	v_11_25	1.59(2.35)	1.85(3.23)	1.00(0.90-1.11)	1.00
SPHK2	*PTK2*	v_11_29	1.40(1.61)	1.72(2.87)	1.01(0.89-1.15)	0.88
SPHK2	*SGK3*	v_11_37	1.49(1.87)	2.01(3.15)	1.03(0.92-1.14)	0.64
DOLK	*BMPR1A*	v_12_13	1.55(1.51)	2.31(3.12)	1.17(1.02-1.33)	0.02
DOLK	*NEK4*	v_12_14	1.51(1.22)	1.70(1.62)	1.05(0.83-1.32)	0.68
DOLK	*BMPR1B*	v_12_16	1.60(2.44)	2.12(2.92)	1.05(0.96-1.14)	0.27
DOLK	*DYRK4*	v_12_17	1.50(1.07)	1.66(1.78)	1.06(0.82-1.36)	0.68
DOLK	*LIMK2*	v_12_18	1.72(2.42)	2.22(3.08)	1.04(0.95-1.14)	0.41
DOLK	*NME4*	v_12_19	1.77(1.93)	3.00(3.95)	1.15(1.04-1.27)	0.01
DOLK	*AK7*	v_12_23	1.39(1.19)	1.75(2.42)	1.07(0.92-1.24)	0.37
DOLK	*WNK1*	v_12_25	1.52(1.52)	2.17(2.93)	1.14(1.01-1.28)	0.03
DOLK	*RIOK2*	v_12_27	1.34(0.93)	1.24(1.20)	0.92(0.67-1.27)	0.62
DOLK	*PTK2*	v_12_29	1.47(1.21)	1.81(2.11)	1.10(0.90-1.34)	0.35
DOLK	*TRIB1*	v_12_30	1.65(2.00)	2.36(2.66)	1.06(0.96-1.17)	0.28
DOLK	*PGK1*	v_12_31	1.28(1.07)	1.58(1.73)	1.11(0.93-1.32)	0.24
DOLK	*STK32A*	v_12_32	1.64(2.09)	1.41(1.24)	0.97(0.80-1.17)	0.75
DOLK	*SGK3*	v_12_37	1.75(1.72)	2.18(2.36)	1.04(0.89-1.22)	0.59
DOLK	*MAGI2*	v_12_38	1.66(1.34)	2.55(2.55)	1.16(1.00-1.33)	0.05
DOLK	*CERKL*	v_12_39	1.51(1.67)	2.37(4.23)	1.06(0.98-1.15)	0.17
DOLK	*CDK4*	v_12_40	1.58(1.92)	2.18(3.88)	1.03(0.95-1.13)	0.47

BMPR1A	*NEK4*	v_13_14	1.64(2.40)	1.59(2.40)	0.96(0.84-1.10)	0.59
BMPR1A	*PRKCD*	v_13_15	1.55(3.28)	1.00(0.54)	0.67(0.37-1.22)	0.19
BMPR1A	*BMPR1B*	v_13_16	1.45(1.84)	1.20(1.10)	0.88(0.69-1.13)	0.32
BMPR1A	*DYRK4*	v_13_17	1.93(3.43)	1.50(2.04)	0.93(0.82-1.06)	0.29
BMPR1A	*LIMK2*	v_13_18	1.40(1.47)	1.17(1.52)	0.89(0.67-1.18)	0.41
BMPR1A	*WNK1*	v_13_25	1.74(3.01)	1.69(2.49)	0.97(0.87-1.09)	0.64
BMPR1A	*PTK2*	v_13_29	1.48(1.63)	1.56(2.19)	0.98(0.83-1.16)	0.84
BMPR1A	*SGK3*	v_13_37	1.63(1.85)	1.93(2.70)	1.01(0.89-1.16)	0.84
BMPR1A	*MAGI2*	v_13_38	1.86(2.78)	2.26(3.63)	1.01(0.92-1.10)	0.84
NEK4	*BMPR1B*	v_14_16	1.20(1.08)	1.30(0.78)	1.08(0.85-1.37)	0.53
NEK4	*DYRK4*	v_14_17	1.11(0.51)	1.08(0.56)	0.91(0.51-1.64)	0.76
NEK4	*LIMK2*	v_14_18	1.29(1.07)	1.34(0.95)	1.05(0.81-1.36)	0.72
NEK4	*NME4*	v_14_19	1.33(1.25)	1.82(1.92)	1.19(1.01-1.39)	0.03
NEK4	*AK7*	v_14_23	1.06(0.67)	1.32(1.34)	1.21(0.93-1.58)	0.16
NEK4	*WNK1*	v_14_25	1.05(0.48)	1.25(0.69)	1.87(1.15-3.02)	0.01
NEK4	*CDC7*	v_14_26	1.13(0.90)	1.69(2.00)	1.22(1.03-1.43)	0.02
NEK4	*DGKB*	v_14_28	1.38(1.91)	1.94(2.37)	1.07(0.97-1.18)	0.20
NEK4	*PTK2*	v_14_29	1.05(0.50)	1.14(0.52)	1.35(0.79-2.31)	0.28
NEK4	*TRIB1*	v_14_30	1.15(0.84)	1.76(1.84)	1.29(1.08-1.56)	0.01
NEK4	*PRKCB*	v_14_33	1.14(1.02)	2.16(3.69)	1.09(1.01-1.18)	0.03
NEK4	*MYLK*	v_14_34	1.48(1.61)	1.68(1.44)	1.07(0.92-1.25)	0.40
NEK4	*MAP2K1*	v_14_36	1.32(1.81)	2.03(2.63)	1.08(0.98-1.18)	0.10
NEK4	*SGK3*	v_14_37	1.20(0.71)	1.50(1.02)	1.41(0.98-2.03)	0.06
NEK4	*MAGI2*	v_14_38	1.20(0.62)	1.55(0.84)	1.58(1.11-2.26)	0.01
NEK4	*CERKL*	v_14_39	1.11(0.85)	1.62(1.96)	1.16(0.97-1.38)	0.10
NEK4	*CDK4*	v_14_40	1.14(0.93)	1.76(2.19)	1.16(1.00-1.35)	0.05
PRKCD	*BMPR1B*	v_15_16	1.34(2.04)	1.39(1.06)	0.99(0.85-1.15)	0.89
PRKCD	*LIMK2*	v_15_18	1.15(0.57)	1.19(0.96)	1.15(0.75-1.76)	0.53
PRKCD	*BMP2K*	v_15_22	1.17(0.78)	1.62(1.47)	1.44(1.12-1.86)	0.005

PRKCD	*WNK1*	v_15_25	1.49(1.82)	2.03(3.26)	1.04(0.94-1.16)	0.44
PRKCD	*PTK2*	v_15_29	1.43(1.67)	1.98(3.65)	1.04(0.94-1.15)	0.45
PRKCD	*MYLK*	v_15_34	1.32(1.06)	1.35(1.04)	1.04(0.80-1.34)	0.78
PRKCD	*PI4K2B*	v_15_35	1.44(1.73)	1.80(1.77)	1.05(0.92-1.20)	0.45
PRKCD	*SGK3*	v_15_37	1.64(2.22)	2.42(4.12)	1.04(0.95-1.12)	0.41
PRKCD	*MAGI2*	v_15_38	1.72(2.69)	2.51(3.78)	1.03(0.96-1.12)	0.39
BMPR1B	*LIMK2*	v_16_18	1.41(1.62)	1.29(1.62)	0.91(0.74-1.12)	0.38
BMPR1B	*BMP2K*	v_16_22	1.30(1.27)	1.84(2.89)	1.07(0.96-1.20)	0.20
BMPR1B	*CDC7*	v_16_26	1.44(2.04)	1.79(3.05)	1.01(0.91-1.12)	0.85
BMPR1B	*PTK2*	v_16_29	1.61(3.18)	1.41(1.74)	0.94(0.81-1.09)	0.41
BMPR1B	*MYLK*	v_16_34	1.50(1.71)	1.33(1.11)	0.91(0.74-1.13)	0.40
BMPR1B	*PI4K2B*	v_16_35	1.94(2.72)	2.18(2.52)	0.98(0.88-1.09)	0.74
DYRK4	*LIMK2*	v_17_18	1.42(1.95)	1.60(1.55)	1.02(0.89-1.17)	0.76
DYRK4	*NME4*	v_17_19	1.30(1.10)	1.77(1.34)	1.23(1.01-1.51)	0.04
DYRK4	*AK7*	v_17_23	1.14(0.88)	1.67(2.92)	1.07(0.96-1.19)	0.21
DYRK4	*WNK1*	v_17_25	1.09(0.58)	1.42(1.18)	1.35(1.03-1.76)	0.03
DYRK4	*CDC7*	v_17_26	1.20(1.26)	1.62(1.46)	1.17(0.98-1.40)	0.08
DYRK4	*DGKB*	v_17_28	1.46(2.09)	2.04(2.74)	1.05(0.96-1.16)	0.27
DYRK4	*PTK2*	v_17_29	1.04(0.50)	1.58(2.48)	1.10(0.98-1.23)	0.12
DYRK4	*TRIB1*	v_17_30	1.12(0.82)	1.81(1.79)	1.27(1.08-1.51)	0.01
DYRK4	*PRKCB*	v_17_33	1.18(1.09)	2.49(4.91)	1.07(1.01-1.13)	0.03
DYRK4	*MYLK*	v_17_34	1.68(2.94)	1.75(1.54)	1.01(0.91-1.12)	0.86
DYRK4	*MAP2K1*	v_17_36	1.43(2.19)	2.05(1.89)	1.07(0.97-1.18)	0.18
DYRK4	*SGK3*	v_17_37	1.21(0.83)	1.59(1.00)	1.38(1.02-1.87)	0.04
DYRK4	*MAGI2*	v_17_38	1.20(0.70)	1.96(2.21)	1.15(1.02-1.30)	0.02
DYRK4	*CERKL*	v_17_39	1.17(0.95)	1.78(2.26)	1.13(0.98-1.31)	0.09
DYRK4	*CDK4*	v_17_40	1.23(1.38)	1.71(1.86)	1.11(0.96-1.29)	0.16
LIMK2	*BMP2K*	v_18_22	1.21(0.98)	1.54(1.29)	1.18(0.94-1.46)	0.15
LIMK2	*PI4K2B*	v_18_35	1.74(2.60)	1.78(1.56)	0.99(0.87-1.12)	0.82

LYN	*CDC7*	v_20_26	1.68(3.31)	1.46(1.54)	0.96(0.85-1.08)	0.50
LYN	*DGKB*	v_20_28	1.56(2.42)	1.81(2.76)	1.01(0.92-1.12)	0.80
LYN	*PTK2*	v_20_29	1.31(1.26)	1.30(2.02)	0.95(0.77-1.18)	0.65
LYN	*TRIB1*	v_20_30	1.28(1.32)	1.53(1.48)	1.03(0.85-1.24)	0.80
LYN	*PRKCB*	v_20_33	1.61(2.08)	2.29(4.87)	1.03(0.96-1.11)	0.44
LYN	*MYLK*	v_20_34	1.80(2.18)	1.44(1.31)	0.91(0.76-1.09)	0.32
LYN	*MAP2K1*	v_20_36	1.77(2.71)	1.78(1.95)	0.99(0.88-1.11)	0.82
LYN	*SGK3*	v_20_37	1.26(0.73)	1.33(0.83)	1.02(0.68-1.53)	0.93
LYN	*MAGI2*	v_20_38	1.34(0.77)	1.63(1.86)	1.09(0.90-1.32)	0.39
LYN	*CDK4*	v_20_40	1.71(3.33)	1.62(1.93)	0.97(0.87-1.10)	0.67
NEK9	*PGK1*	v_21_31	1.87(2.60)	2.06(3.23)	0.98(0.88-1.09)	0.69
BMP2K	*PI4K2B*	v_22_35	1.75(2.43)	1.47(1.58)	0.90(0.76-1.06)	0.21
AK7	*ULK4*	v_23_24	1.43(3.16)	1.36(0.80)	0.98(0.87-1.11)	0.75
AK7	*WNK1*	v_23_25	1.40(1.31)	1.52(1.03)	1.02(0.82-1.27)	0.85
AK7	*CDC7*	v_23_26	1.28(1.19)	1.41(0.90)	1.09(0.86-1.38)	0.46
AK7	*DGKB*	v_23_28	1.50(1.63)	1.82(2.40)	1.06(0.93-1.20)	0.41
AK7	*PTK2*	v_23_29	1.32(1.11)	1.49(1.09)	1.05(0.83-1.33)	0.67
AK7	*TRIB1*	v_23_30	1.54(1.73)	1.99(2.47)	1.07(0.93-1.22)	0.34
AK7	*PRKCB*	v_23_33	1.23(1.31)	1.87(4.16)	1.05(0.97-1.13)	0.25
AK7	*MYLK*	v_23_34	1.98(3.47)	2.01(2.09)	1.00(0.91-1.09)	0.96
AK7	*MAP2K1*	v_23_36	1.40(1.30)	1.78(1.47)	1.18(0.97-1.42)	0.09
AK7	*SGK3*	v_23_37	1.61(1.65)	1.94(1.80)	1.05(0.89-1.24)	0.54
AK7	*MAGI2*	v_23_38	1.57(1.45)	2.12(1.80)	1.12(0.95-1.32)	0.17
AK7	*CERKL*	v_23_39	1.16(0.75)	1.28(0.88)	1.06(0.76-1.49)	0.74
AK7	*CDK4*	v_23_40	1.21(0.83)	1.34(0.85)	1.13(0.82-1.57)	0.45
ULK4	*WNK1*	v_24_25	1.48(1.39)	1.46(1.39)	0.96(0.77-1.20)	0.73
ULK4	*CDC7*	v_24_26	1.25(0.79)	1.17(0.63)	0.88(0.59-1.30)	0.52
ULK4	*DGKB*	v_24_28	1.53(1.38)	1.50(1.26)	0.97(0.78-1.22)	0.82
ULK4	*PTK2*	v_24_29	1.43(1.23)	1.55(1.65)	1.02(0.83-1.25)	0.86

ULK4	*TRIB1*	v_24_30	1.42(1.71)	1.58(1.25)	1.01(0.86-1.19)	0.86
ULK4	*PRKCB*	v_24_33	1.33(1.41)	1.39(1.50)	1.01(0.84-1.21)	0.93
ULK4	*MYLK*	v_24_34	2.02(3.19)	1.68(1.65)	0.96(0.84-1.10)	0.53
ULK4	*MAP2K1*	v_24_36	1.59(1.52)	1.79(1.67)	1.05(0.89-1.24)	0.58
ULK4	*SGK3*	v_24_37	1.79(2.13)	2.12(2.43)	1.02(0.90-1.15)	0.79
ULK4	*MAGI2*	v_24_38	1.77(1.89)	2.34(3.04)	1.04(0.93-1.16)	0.46
ULK4	*CERKL*	v_24_39	1.15(0.51)	1.06(0.47)	0.72(0.38-1.37)	0.32
ULK4	*CDK4*	v_24_40	1.24(0.74)	1.31(0.94)	1.07(0.75-1.54)	0.69
WNK1	*CDC7*	v_25_26	1.22(1.31)	1.38(1.34)	1.03(0.85-1.26)	0.74
WNK1	*DGKB*	v_25_28	1.63(3.46)	1.55(1.39)	0.99(0.89-1.09)	0.79
WNK1	*PTK2*	v_25_29	1.12(0.54)	1.14(0.77)	1.00(0.61-1.65)	1.00
WNK1	*TRIB1*	v_25_30	1.24(1.12)	1.50(1.49)	1.07(0.86-1.32)	0.56
WNK1	*PRKCB*	v_25_33	1.34(1.56)	1.86(2.59)	1.07(0.94-1.21)	0.31
WNK1	*MYLK*	v_25_34	1.68(2.85)	1.47(1.59)	0.97(0.84-1.12)	0.67
WNK1	*MAP2K1*	v_25_36	1.40(2.01)	1.82(2.04)	1.05(0.94-1.16)	0.38
WNK1	*SGK3*	v_25_37	1.21(0.71)	1.30(0.77)	1.05(0.69-1.58)	0.83
WNK1	*MAGI2*	v_25_38	1.20(0.58)	1.51(0.92)	1.44(1.00-2.06)	0.05
WNK1	*CERKL*	v_25_39	1.40(2.08)	1.42(1.61)	0.97(0.83-1.14)	0.71
WNK1	*CDK4*	v_25_40	1.48(3.19)	1.82(2.96)	1.01(0.94-1.10)	0.73
CDC7	*PRKCB*	v_26_33	1.85(3.34)	1.58(2.32)	0.95(0.85-1.07)	0.43
CDC7	*CERKL*	v_26_39	1.59(2.18)	1.26(1.14)	0.87(0.69-1.08)	0.21
CDC7	*CDK4*	v_26_40	1.37(1.39)	1.39(1.45)	0.97(0.79-1.19)	0.75
DGKB	*PTK2*	v_28_29	1.56(1.74)	1.67(2.53)	1.00(0.86-1.16)	0.98
DGKB	*TRIB1*	v_28_30	1.94(3.31)	1.74(2.29)	0.96(0.86-1.07)	0.45
DGKB	*MYLK*	v_28_34	1.90(2.32)	1.91(3.15)	1.00(0.88-1.12)	0.95
DGKB	*MAP2K1*	v_28_36	1.40(1.12)	1.41(0.88)	1.00(0.75-1.33)	0.99
DGKB	*SGK3*	v_28_37	1.64(1.72)	2.34(4.52)	1.05(0.97-1.15)	0.24
DGKB	*MAGI2*	v_28_38	1.58(1.79)	2.53(4.83)	1.05(0.98-1.12)	0.19
PTK2	*TRIB1*	v_29_30	1.17(1.01)	1.63(1.49)	1.19(0.99-1.44)	0.06

PTK2	*PRKCB*	v_29_33	1.23(1.25)	1.72(1.90)	1.12(0.96-1.31)	0.14
PTK2	*MYLK*	v_29_34	1.57(1.97)	2.07(4.40)	1.07(0.98-1.17)	0.16
PTK2	*MAP2K1*	v_29_36	1.39(1.60)	2.34(3.89)	1.12(1.02-1.22)	0.01
PTK2	*SGK3*	v_29_37	1.19(0.65)	1.43(0.90)	1.30(0.89-1.91)	0.18
PTK2	*MAGI2*	v_29_38	1.23(0.64)	1.58(1.04)	1.38(1.02-1.87)	0.04
PTK2	*CERKL*	v_29_39	1.29(1.42)	1.61(1.89)	1.06(0.90-1.24)	0.47
PTK2	*CDK4*	v_29_40	1.26(1.42)	1.64(1.73)	1.08(0.93-1.25)	0.32
TRIB1	*SGK3*	v_30_37	1.39(0.94)	1.22(0.96)	0.83(0.58-1.18)	0.29
TRIB1	*MAGI2*	v_30_38	1.63(1.81)	1.36(1.14)	0.88(0.67-1.16)	0.36
PGK1	*STK32A*	v_31_32	1.59(1.96)	1.34(1.10)	0.93(0.75-1.17)	0.55
PGK1	*MAP2K1*	v_31_36	1.74(2.58)	2.85(3.14)	1.07(0.99-1.15)	0.07
PGK1	*SGK3*	v_31_37	1.97(2.93)	2.17(2.22)	1.01(0.89-1.13)	0.93
PGK1	*MAGI2*	v_31_38	1.88(2.93)	2.26(2.50)	1.02(0.93-1.13)	0.63
PGK1	*CERKL*	v_31_39	1.66(2.46)	2.10(3.30)	1.04(0.94-1.16)	0.42
STK32A	*PRKCB*	v_32_33	1.91(2.92)	2.41(3.41)	1.02(0.94-1.10)	0.62
STK32A	*MAGI2*	v_32_38	2.21(2.34)	2.87(3.76)	1.04(0.95-1.15)	0.36
STK32A	*CERKL*	v_32_39	1.94(2.50)	2.03(2.94)	1.01(0.90-1.13)	0.88
STK32A	*CDK4*	v_32_40	2.14(3.24)	2.00(2.38)	0.98(0.88-1.09)	0.73
PRKCB	*MYLK*	v_33_34	1.75(2.22)	1.71(2.09)	1.00(0.87-1.15)	0.98
PRKCB	*MAP2K1*	v_33_36	1.31(0.92)	1.63(1.57)	1.25(0.99-1.58)	0.06
PRKCB	*SGK3*	v_33_37	1.48(1.21)	1.75(1.71)	1.07(0.87-1.32)	0.53
PRKCB	*MAGI2*	v_33_38	1.45(1.05)	1.96(2.35)	1.10(0.95-1.27)	0.21
PRKCB	*CERKL*	v_33_39	1.33(1.67)	1.07(0.70)	0.83(0.57-1.21)	0.33
PRKCB	*CDK4*	v_33_40	1.29(1.23)	1.18(0.81)	0.93(0.68-1.27)	0.65
MYLK	*PI4K2B*	v_34_35	1.58(1.90)	1.56(1.76)	0.94(0.79-1.12)	0.48
MAP2K1	*SGK3*	v_36_37	1.54(1.58)	2.33(4.84)	1.04(0.96-1.12)	0.37
MAP2K1	*CDK4*	v_36_40	1.50(2.00)	1.34(1.68)	0.94(0.78-1.13)	0.51
SGK3	*MAGI2*	v_37_38	1.38(1.45)	1.65(1.60)	1.05(0.90-1.24)	0.51
CERKL	*CDK4*	v_39_40	1.12(0.54)	1.25(0.72)	1.35(0.84-2.20)	0.22

Table S8. Gene ratios associated with recurrence and survival

Variables	No recurrence mean (SD)	Recurrence mean (SD)	HR (95%CI)	P value
**Training set**				
PTK2/MAGI2	1.35(0.81)	1.70(1.25)	1.57(1.07-2.31)	**0.02**
CDKL4/PTK2	1.08(0.53)	1.59(2.25)	1.34(1.07-1.69)	**0.01**
**Replication set**				
PTK2/MAGI2	1.21(0.62)	1.25(0.43)	1.17(0.53-2.56)	0.70
CDKL4/PTK2	1.23(0.85)	1.61(1.41)	1.45(0.97-2.19)	0.07
**Pooled analysis**				
PTK2/MAGI2	1.29(0.73)	1.47(0.95)	1.42(0.99-2.02)	**0.05**
CDKL4/PTK2	1.15(0.70)	1.60(1.85)	1.32(1.09-1.59)	**0.004**
**Variables**	**Alive mean (SD)**	**Dead mean (SD)**	**HR (95%CI)**	**P value**
**Training set**				
PRKCD/BMP2K	1.27(1.00)	1.81(1.71)	1.35(1.03-1.78)	**0.03**
MST1R/WNK1	1.41(0.91)	2.00(1.49)	1.50(1.13-1.98)	**0.005**
STK39/AGK	1.03(0.55)	1.41(0.93)	2.43(1.41-4.19)	**0.001**
DYRK4/SGK3	1.30(0.89)	1.69(1.05)	1.47(1.01-2.15)	**0.05**
CDKL4/CERKL	1.08(0.93)	2.22(2.95)	1.19(1.04-1.35)	**0.01**
**Replication set**				
PRKCD/BMP2K	1.07(0.46)	1.26(0.80)	2.02(0.78-5.27)	0.15
MST1R/WNK1	1.09(0.53)	1.16(0.48)	1.29(0.47-3.52)	0.62
STK39/AGK	1.13(0.96)	1.49(0.60)	1.14(0.79-1.65)	0.49
DYRK4/SGK3	1.13(0.77)	1.40(0.91)	1.24(0.71-2.14)	0.45
CDKL4/CERKL	1.12(0.66)	1.67(1.27)	1.97(1.18-3.29)	**0.01**
**Pooled analysis**				
PRKCD/BMP2K	1.17(0.78)	1.62(1.47)	1.44(1.12-1.86)	**0.005**

MST1R/WNK1	1.25(0.75)	1.71(1.29)	1.45(1.11-1.90)	**0.01**
STK39/AGK	1.08(0.79)	1.44(0.82)	1.35(1.06-1.73)	**0.01**
DYRK4/SGK3	1.21(0.83)	1.59(1.00)	1.38(1.02-1.87)	**0.04**
CDKL4/CERKL	1.10(0.80)	2.03(2.49)	1.21(1.07-1.35)	**0.001**
